# Genome-wide identification and expression profiling of two-component system (TCS) genes in *Brassica oleracea* in response to shade stress

**DOI:** 10.3389/fgene.2023.1142544

**Published:** 2023-05-30

**Authors:** Muhammad Sadaqat, Basit Umer, Kotb A. Attia, Amr F. Abdelkhalik, Farrukh Azeem, Muhammad Rizwan Javed, Kinza Fatima, Roshan Zameer, Majid Nadeem, Muhammad Hammad Tanveer, Sangmi Sun, Sezai Ercisli, Muhammad Amjad Nawaz

**Affiliations:** ^1^ Department of Bioinformatics and Biotechnology, Government College University Faisalabad (GCUF), Faisalabad, Pakistan; ^2^ Department of Biochemistry, College of Science, King Saud University, Riyadh, Saudi Arabia; ^3^ Biotechnology School, Nile University, Giza, Egypt; ^4^ Rice Biotechnology Lab, Rice Research and Training Center, Field Crops Research Institute, ARC, Kafrelshikh, Egypt; ^5^ Wheat Research Institute, Ayub Agriculture Research Institute, Faisalabad, Pakistan; ^6^ Department of Biotechnology, Chonnam National University, Yesosu Campus, Yesosu Si, Republic of Korea; ^7^ Department of Horticulture, Faculty of Agriculture, Ataturk University, Erzurum, Türkiye; ^8^ HGF Agro, Ata Teknokent, Erzurum, Türkiye; ^9^ Advanced Engineering School (Agrobiotek), Tomsk State University, Tomsk, Russia; ^10^ Center for Research in the Field of Materials and Technologies, Tomsk State University, Tomsk, Russia

**Keywords:** cabbage, two-component system, cytokinin, cell signaling, abiotic stress, genome-wide, expression analysis, phylogeny

## Abstract

The Two-component system (TCS) consists of Histidine kinases (HKs), Phosphotransfers (HPs), and response regulator (RR) proteins. It has an important role in signal transduction to respond to a wide variety of abiotic stresses and hence in plant development. *Brassica oleracea* (cabbage) is a leafy vegetable, which is used for food and medicinal purposes. Although this system was identified in several plants, it had not been identified in *Brassica oleracea* yet. This genome-wide study identified 80 *BoTCS* genes consisting of 21 HKs, 8 HPs, 39 RRs, and 12 PRRs. This classification was done based on conserved domains and motif structure. Phylogenetic relationships of *BoTCS* genes with *Arabidopsis thaliana*, *Oryza sativa*, *Glycine max,* and *Cicer arietinum* showed conservation in TCS genes. Gene structure analysis revealed that each subfamily had conserved introns and exons. Both tandem and segmental duplication led to the expansion of this gene family. Almost all of the HPs and RRs were expanded through segmental duplication. Chromosomal analysis showed that *BoTCS* genes were dispersed across all nine chromosomes. The promoter regions of these genes were found to contain a variety of cis-regulatory elements. The 3D structure prediction of proteins also confirmed the conservation of structure within subfamilies. MicroRNAs (miRNAs) involved in the regulation of *BoTCSs* were also predicted and their regulatory roles were also evaluated. Moreover, BoTCSs were docked with abscisic acid to evaluate their binding. RNA-seq-based expression analysis and validation by qRT-PCR showed significant variation of expression for *BoPHYs, BoERS1.1, BoERS2.1, BoERS2.2, BoRR10.2,* and *BoRR7.1* suggesting their importance in stress response. These genes showing unique expression can be further used in manipulating the plant’s genome to make the plant more resistant the environmental stresses which will ultimately help in the increase of plant’s yield. More specifically, these genes have altered expression in shade stress which clearly indicates their importance in biological functions. These findings are important for future functional characterization of TCS genes in generating stress-responsive cultivars.

## 1 Introduction

Two-component system (TCS) is a major signal transduction system that is involved in several biological processes by regulating the signaling of histidine-aspartate. Thus, it responses to environmental stimuli such as cell growth and division, oxygen sensing, osmotic sensing, host recognition, and chemotaxis in both prokaryotes and eukaryotes. In bacteria, the two-component system relies on the phosphorylation of aspartic acid and histidine residues. Moreover, this system has also been identified in slime molds and fungi (Schaller et al., 2008; Mochida et al., 2010). Protein phosphorylation is one of the most common methods for intracellular signaling. Protein kinases use ATP as the phosphate donor to phosphorylate their substrates. They are divided into five classes based on the receptor amino acid: histidine kinases (HK), serine-threonine kinases (STK), cysteine kinases (CK), tyrosine kinases (TK), and aspartyl kinases (AK) (Hunter, 1991).

Plants use two-component signaling elements in a variety of signaling pathways, the best known of which is cytokinin signaling (Tsai et al., 2012; Ahmad et al., 2020). A His-to-Asp phosphorelay is involved in the signaling of the plant hormone cytokinin. The HKs associated sensory extracellular extracytoplasmic hormone-binding domain (CHASE), as well as the receiver and cytoplasmic HK, make up the classic cytokinin receptor (Halawa et al., 2021). The functionality of HKs is based on TCS. This system consists of a His-kinase that recognizes the environmental stimuli and autophosphorylates its conserved histidine (His) residue, which is subsequently transferred to a conserved aspartic acid (Asp) residue in another group of signal transducers known as response regulators (RRs). Phosphorylation of RRs provides the ability to control the downstream signaling, allowing the external stimulus to be converted into an internal signal (Wallmeroth et al., 2019). In bacteria, fungi (slime molds, yeast), and plants a His-to-Asp phosphorelay is formed which is more complex and uses a hybrid kinase including both a receiver domain (Rec) and an HK domain in a single protein. Another domain called His-containing phosphotransfer (HP) domain is also present in both prokaryotes and in eukaryotes which is a signaling component. The phosphate is transported from HK to RR through a multistep His-to-Asp phosphorelay (Asakura et al., 2003).

In plants, three distinct families of HKs are present: cytokinin receptors, phytochromes, and ethylene receptors which play an important role in phytochrome signaling. Although TCS elements have all the necessary conserved residues, phytochromes, and ethylene receptors are divergent signaling elements that do not have conserved residues for phosphotransfer activity in their HK motif (Zameer et al., 2021). In Arabidopsis*,* there are three kinases (*AHK1*, *AHK5,* and *CKl1*) that do not belong to the cytokinin receptors. Cytokinin receptors’ basic structure consists of an input domain, a Rec domain, a transmitter domain (having a conserved histidine/H residue that provides the site of autophosphorylation), and many trans-membrane domains. Another domain called cyclase/CHASE domain is also present in three cytokinin receptors (AHK2/3/4) which is a possible cytokinin recognition site. A C_2_H_4_/ethylene binding domain is shared by five ethylene receptors (ETR1/2, ERS1/2, and EIN4). Five phytochromes (PHYA, B, C, D, and E) share a chromophore binding (PHY) domain and two PAS (Per/Arndt/Sim) folds. In Arabidopsis*,* a conserved motif “XHQXKGSSXS” is present in AHPs that regulates the transfer of phosphate group HK’s receiver to the RR’s Rec domain. AHP6 lacks the conserved His residue, therefore, is called a pseudo-His-containing phosphotransfer (HP) protein (Z. Liu et al., 2014).

Response regulators (RRs) are classified into four groups based on domain structure and phylogenetic analysis: type-A, type-B, type-C, and pseudo-response regulators (PRRs). Type-A RRs are the key transcriptional targets for cytokinin signaling that contain a Rec domain. Type-B RRs function as the positive transcriptional regulator of cytokinin signaling. These RRs contain a Rec as well as Myb-like DNA-binding domain at C-terminus. Type-C RRs also contain the Rec domain and lack the Myb domain. Therefore, these are phylogenetically closer to Type-A RRs (Gahlaut et al., 2014). A multistep phosphorelay comprising cytokinin receptors, type-B RRs, and phosphotransfer proteins mediates the initial steps of cytokinin signaling. Type-B RRs regulate gene expression, including transcriptional activation of Type-A RRs by relaying the cytokinin signal from the membrane to the nucleus. Type-A RRs are negative regulators of the initial signal transduction pathway mediating downstream responses to cytokinin (Sharan et al., 2017). Finally, PRRs have a receiver domain in which the D residue is replaced by E, as well as the C-terminus CCT domain has a function associated with protein-protein interactions (Tan et al., 2019).

Abscisic acid (ABA) and stress responses are significantly mediated by the cytokinin-responsive TCS. Different stresses impose a negative impact on plant development and crop yield. Under osmotic stress, ABA is generated and plays a crucial role in plant stress response as well as in tolerance. During the growth and germination of the seed, it mediates gene expression. Furthermore, it is also found that the ABA and other signaling components interact during seed development and stress response. Controlling the expression of ABA signaling factors may help to cope better with stresses (Tran et al., 2010; Nakashima & Yamaguchi-Shinozaki, 2013). Various abiotic stresses like drought and excessive salinity have a negative impact on plant growth and yield. To survive in this variety of conditions, plants have evolved various complex signaling networks at the system, cellular, and molecular level. In plant cells, the TCS genes mediate phosphorylation, which is a major method of signal transduction regarding stress (Z. Liu et al., 2014). TCS genes have previously been identified in *Arabidopsis* (Cheng & Kieber, 2014), rice (Sharan et al., 2017), banana (Dhar et al., 2019), melon (*Cucumis melo L.*) (P. Liu et al., 2020), cucumber (He et al., 2016a), *C. arietinum* (Ahmad et al., 2020), tomato (He et al., 2016b), soybean (Le et al., 2011) and wheat (Gahlaut et al., 2014). Different TCS genes have different expression patterns. TCS responses to environmental factors vary greatly depending on individual TCS members, conditions, and species. In *C. arietinum*, *Arabidopsis*, soybean, and cucumber, members of the TCS gene family are found to be involved in the regulation of abiotic stresses including dehydration, drought, cold, and salt stress [19, 21, 22]. Similarly, TCS genes in rice are involved in ABA-induced increased expression of antioxidant enzymes and gene activity [12, 46].

*Brassica oleracea L.* var. *Capitata f. Rubra* (Red cabbage) (2 *n* = 18) is a Mediterranean and southwestern European native herbaceous leafy vegetable that currently grows worldwide. It is a member of the Brassicaceae family, with an annual global production of 58 million tons of fresh heads from 3.1 million ha in over 130 countries. Since *Brassica* crops are mostly grown in an arid or semiarid environment, they are subjected to a variety of abiotic stresses. In crops, two strategies can be employed to cope with abiotic stresses. These include biotechnological approaches and crop management. Agronomic methods like irrigation with high-quality water, and changing sowing times can all contribute to reducing the effects of abiotic stresses. For vegetable cultivation, this technique is neither sustainable nor cost-effective. Therefore, a long-term solution for stress-resistant vegetable cultivation is required. Plants contain various stress-related genes and their expression helps to regulate stress tolerance in plants. These stress-related genes are required to produce stress-tolerant crop varieties. This genetic mechanism can be utilized to produce high yields during stressful conditions. The active genes have been exploited to generate stress-tolerant cops (Arapitsas et al., 2008; Wei et al., 2021). Similarly, TCS genes have been found in a variety of plants including *Brassica oleracea*, and they may help to improve food production by resisting a variety of environmental factors. Since, *B. oleracea* is economically important food, getting higher yield by making plant more resistant to stress would be helpful. Therefore, the goal is to identify certain genes important in stress resistance and manipulating them to make plant more resistant to environmental stresses. For this purpose, the functional diversity and expression profiles of *BoTCS* genes were evaluated in this work. In addition, the expression profiling of these genes under shade stress will help to study the stress effect on plant physiology. Our study aimed to discover TCS genes that are responsive to changes in shade, which could be useful in the breeding of *B. oleracea*. As TCS genes have various roles in biological processes, it is crucial to conduct a comprehensive investigation of these genes in *B. oleracea*. By identifying these shade-responsive TCS genes, we hope to contribute to the understanding of the molecular mechanisms underlying the shade response in *B. oleracea* and provide valuable information for future breeding efforts. Our research underscores the importance of studying specific gene families in different crops to uncover their potential roles in important biological processes*.*

## 2 Materials and methods

### 2.1 Identification of *Brassica oleracea TCS* genes and their subcellular localization

For the identification of TCS proteins in *B. oleracea,* amino acid sequences from Arabidopsis TCS proteins were retrieved from the Ensembl plants database (http://plants.ensembl.org/index.htm) and were used as queries in BLASTP searches against NCBI (https://www.ncbi.nlm.nih.gov/) (Jenuth, 2000) database of *B. oleracea*. The default parameters of the BLASTP tool were used for this search. All the retrieved non-redundant protein sequences were subjected to domain analysis using online servers: Pfam (http://pfam.xfam.org/) (Bateman et al., 2004), CDD (https://www.ncbi.nlm.nih.gov/Structure/cdd/wrpsb.cgi) and SMART (http://smart.embl-heidelberg.de/) (Schultz et al., 2000). Protein sequences containing specific domains including histidine-kinases receptor domain, response regulator domain, and other specific domains were kept as candidate TCS proteins, while those without conserved domains were excluded. Details such as exon number, chromosome number, and location were obtained from NCBI.

Physiochemical characteristics of all BoTCS proteins were determined using the ExPASy ProtParam online tool (https://web.expasy.org/protparam/) (Gasteiger et al., 2005), including molecular weight (in Da Da), grand average of hydropathicity (GRAVY), isoelectric point (pI, point at which molecule is electrically neutral), instability index (II) and aliphatic index (AI). The subcellular localizations were predicted with the online CELLO v.2.5 tool (http://cello.life.nctu.edu.tw/) (Yu et al., 2004).

### 2.2 Phylogenetic, gene structure, and conserved motif analysis

To determine the evolutionary relationship of TCS proteins from *B. oleracea* and diverse plant species, protein sequences of *B. oleracea, Arabidopsis thaliana*, *C. arietinum*, *Glycine max,* and *Oryza sativa* were used*.* Multiple sequence alignment of these proteins was generated using the ClustalW tool and a Maximum-likelihood (ML) tree was constructed by using IQ-Tree (Trifinopoulos et al., 2016) with a bootstrap value of 1,000 and was edited online using iTOL: Interactive Tree of Life (https://itol.embl.de/) (Letunic & Bork, 2021).

The Gene Structure Display Server 2.0 (GSDS) (http://gsds.gao-lab.org/) (Hu et al., 2015) was used to predict gene exon/intron structure using coding (CDS) and genomic sequences of *B. oleracea* and *A. thaliana* as input data. The CDS and genomic sequences were retrieved through NCBI. The MEME (Multiple EM for Motif Elicitation) tools (https://meme-suite.org/meme/) (Bailey et al., 2015) were used to determine the conserved motifs in the Bo*TCS* sequence. The maximum motif number was set to 20, all other parameters were left at their default values and the meme.xml file was obtained. This file was used to analyze conserved motifs using TBtools.

### 2.3 Identification of cis-regulatory elements and chromosomal mapping, gene duplication events, and syntenic analysis of *BoTCS* genes

The 2 kb upstream sequences from the translation start site of *BoTCS* genes were retrieved from the NCBI-nucleotide database (https://www.ncbi.nlm.nih.gov/nucleotide/) (Orlek et al., 2017). To examine the cis-elements in these promoter sequences, the PlantCARE online server (http://bioinformatics.psb.ugent.be/webtools/plantcare/html/) (Rombauts et al., 1999) was used and TBtools simple bioSequence viewer was used to visualize results. A genetic relational map of *B. oleracea* chromosomes was created by using TBtools advanced Circos. The position of each *B. oleracea TCS* gene on its chromosomes was predicted using the NCBI Gene database (https://www.ncbi.nlm.nih.gov/gene) (Sanchez, 2013; Brown et al., 2015). Similarly, TBtools was used to perform syntenic analysis (Chen et al., 2018). The gene duplication events were analyzed by using DnaSP v.6 tool (Rozas et al., 2017). Further, their synonymous as well as non-synonymous substitution rates were also calculated. To analyze their evolutionary events, the divergence time was calculated. The following formula was used to calculate the time of duplication: T = Ks/2x (x = 6.56 * 10^−9^) (He et al., 2016a; Zia et al., 2022).

### 2.4 miRNA target prediction

The Plant microRNA Encyclopedia database (PmiREN; https://pmiren.com) (Guo et al., 2020) was utilized to acquire mature miRNA sequences of *B. oleracea*. For putative miRNA target prediction CDS sequences of the potential target, BoTCSs were utilized and were submitted at the psRNATarget server (https://www.zhaolab.org/psRNATarget/home) (Dai et al., 2018) along with the respective mature miRNA sequences of *B. oleracea* with default considerations. The regulatory association between target BoTCSs and predicted miRNAs was visualized using Cytoscape software (Kohl et al., 2011).

### 2.5 Three-dimensional structure of BoTCSs and molecular docking analysis

The three-dimensional (3D) structure of the protein is essential for a proper understanding of its functions. Therefore, the I-TASSER web server was used to predict the 3D structures of BoTCS proteins (https://zhanggroup.org/I-TASSER/) (Yang et al., 2015). The UCSF Chimera tool was used to visualize the 3D structures (Pettersen et al., 2004). In addition, models were verified using the SAVES v6.0 web server (https://saves.mbi.ucla.edu/) (Elshemey et al., 2010). The 3D structure of ABA was retrieved using PubChem (https://pubchem.ncbi.nlm.nih.gov/) ID (5,280,896). Further, to check the binding pattern of these proteins with specific ligand, molecular docking analysis was performed which showed the interactions as well as interacting residues among the proteins and ligand. The molecular docking analysis of BoTCSs and ABA was done using PyRx virtual screening software (https://pyrx.sourceforge.io/downloads). Interaction study of protein-ligand complexes was also done using UCSF Chimera (Pettersen et al., 2004) and Discovery Studio after the successful docking of ABA ligand against all 80 BoTCSs.

### 2.6 Differential expression profiling of *BoTCS* genes under different stress conditions

RNA-seq data for light/shade [BioProject: SRR14774565] and tissue-specific responses [BioProject: SRR13759392] was retrieved from the NCBI-SRA database (https://www.ncbi.nlm.nih.gov/sra) to investigate the differential expression pattern of TCS genes in *B. oleracea*. This data was used to compare the expression pattern of *BoTCS* genes in shade and light at specific times (1h and 6 h after stress application). For tissue-specific responses, SRA data for leaf, bud, stem, root, silique, flower, and the leafy head was used. The genome and GFF files were downloaded from the Phytozome database (https://phytozome-next.jgi.doe.gov/). Bowtie2 tool (Colling et al., 2013) was used to build genomic sequence indexes for *B. oleracea* and further the genome was mapped using pair-end clean reads. The alignment file was then used in the next step of abundance estimation which provided the differential expression of genes. The expression level of genes was estimated using the cufflinks tool, which provided FPKM values. FPKM values were used to generate a heatmap (Azeem et al., 2022; Zameer et al., 2022). Heatmap was generated using TBtools (Chen et al., 2018).

### 2.7 Plant growth, treatment, and quantitative real-time PCR validation

Seedlings of cabbage (*B. oleracea* var. *capitata*) were grown at 23°C ± 2°C for 25 days under natural light. For shade treatment-seedlings were kept in shade for specific periods. First, the duration for keeping seedlings in shade was 1 h and then the second time the duration was increased to 6 h. For RNA extraction leaves/roots of plants were collected and sampled as a biological replicate. These samples were immediately frozen in liquid nitrogen and kept at −80°C until needed.

TRIzol reagent was used to extract RNA from leaf samples as directed by the manufacturer and quantified using a NanoDrop spectrophotometer (Shahriari & Tahmasebi, 2018). One microgram of total RNA was reverse transcribed with dsDNase using the Maxima H Minus First-strand cDNA synthesis kit. 10X dsDNase buffer, total RNA, dsDNase, and nuclease-free H_2_O were added to an RNase-free tube on ice to obtain a total volume of 10 mL. The mixture was gently mixed and spun before being incubated at 37°C for 2 min in a hot water bath and then stored on ice. To synthesize the strands, chemicals (5X RT buffer, 10 mM dNTP mix, oligoT primer, and maxima H minus reverse transcriptase enzyme) were then added and gently mixed before being centrifuged briefly. The mixture was then incubated for 30 min at 50°C and 5 min at 85°C. Finally, the mixture was kept at a temperature of around −80°C until it was used. iTaq universal SYBR Green Super Mix and Real-time PCR detection system (CFX96 Touch™ Real-TimePCR Detection System were used to carry out qRT-PCR. Primers were designed using the online NCBI Primer-BLAST Program (https://www.ncbi.nlm.nih.gov/tools/primer-blast/) (Ye et al., 2012) and their specificity was verified by using an online tool Oligo Calculator (http://mcb.berkeley.edu/labs/krantz/tools/oligocalc.html) (Kibbe, 2007). The overall workflow of this study is presented in Supplementary Figure S1.

### 2.8 Data analysis

RNA-seq analysis provided FPKM values which were further analyzed to find differentially expressed genes. In tissue-specific expression analysis, genes with zero FPKM values were considered as having no change in expression and higher values were supposed to have an increasing expression. While in shade stress, genes having FPKM values greater than one were considered as upregulated. Whereas, genes having FPKM values less than one were considered as downregulated ones. qRT-PCR results were analyzed by applying Student’s t-test (*p* < 0.05 = > *, *p* < 0.01 = >**) to compare control and shade stress and calculated the significance level.

## 3 Results

### 3.1 Identification of TCS proteins in *Brassica oleracea*

A total of 80 TCS genes; 21 HKs, 8 HPs, 39 RRs, and 12 PRRs, were identified in *B. oleracea* after checking for specific domains and removal of redundancy. The identified TCS members were named according to the proposed nomenclature in previous studies on this gene family on other plants. The tree shows the evolutionary relationships among the members of this gene family.

#### 3.1.1 HK protein family in *Brassica oleracea*

The genome of *B. oleracea* has 21 members of the HK family, which is comparable to the other species including Soybean (21), *M. acuminata,* and *M. balbisiana* (25) (Supplementary Table S2). The HK family is further classified into three subgroups CHK/HK, PHY, and ETR. Out of 21 HK genes identified in *B. oleracea*, 9 members were classified as CHK/HK. Both PHY and ETR subgroups contained six members each. All of the HKs possess a conserved HK domain that contains five conserved signature motifs, namely, H, N, G1, F, and G2.

**FIGURE 1:**
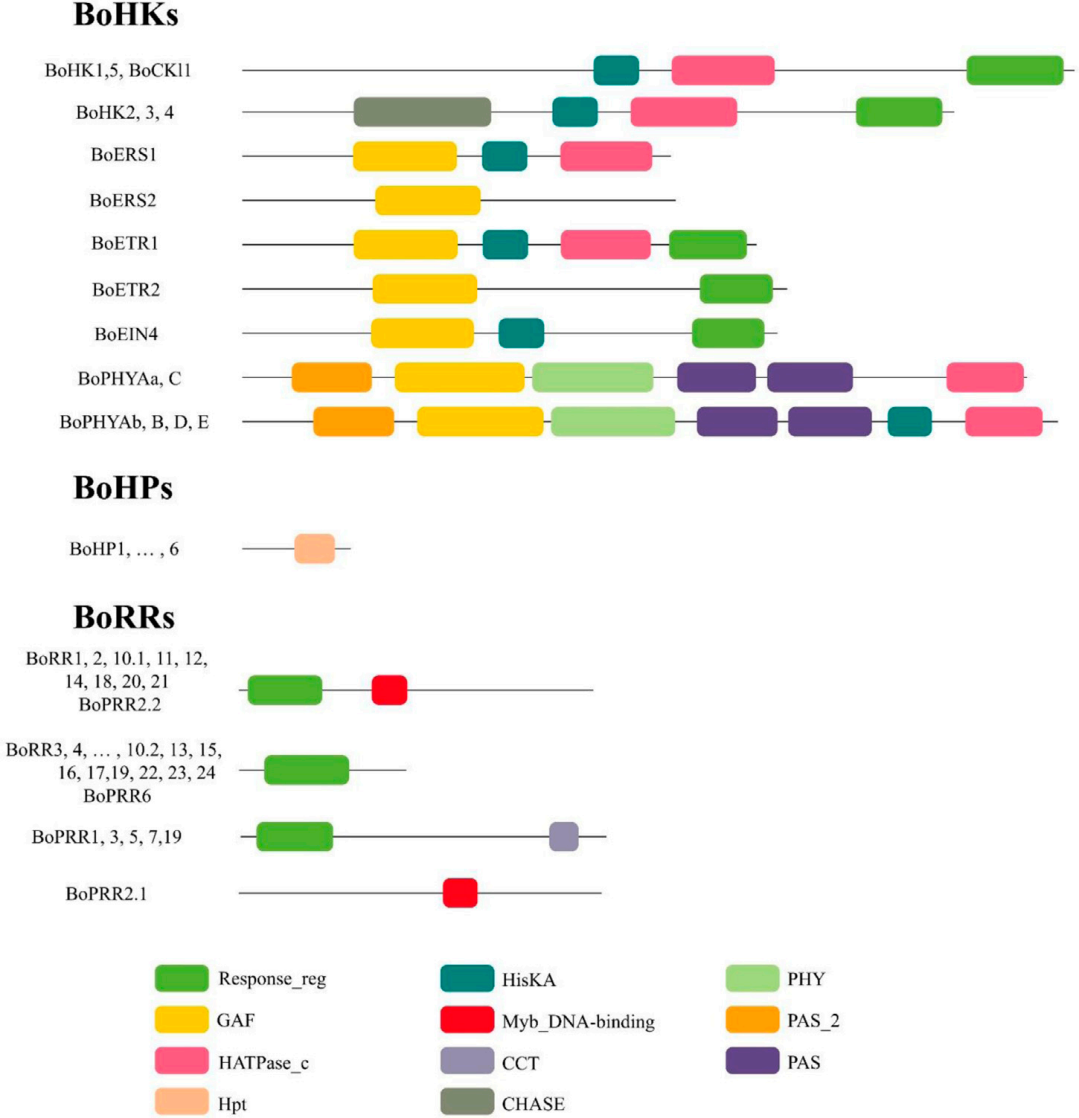
Symbolic domain architecture of the Two-Component System signaling elements in *B. oleracea*. Each sequence contains a specific set and number of domains which are represented by a specific-colored shape.

**FIGURE 2:**
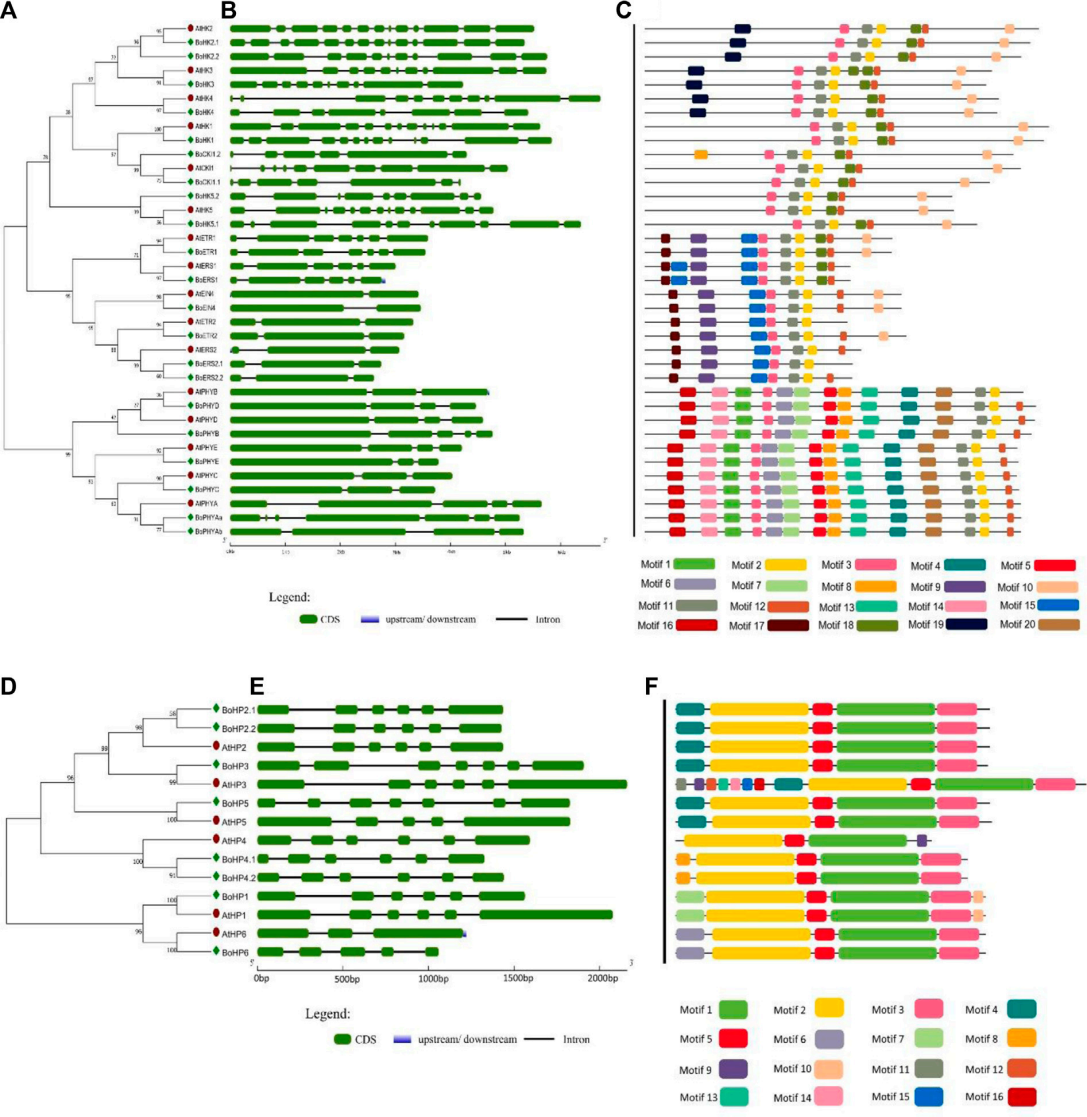
Gene structure and conserved motifs of Histidine Kinases and Phosphotransferases in *B. oleracea*. **(A)** and **(D)** The phylogenetic trees of BoHKs and BoHPs were constructed using MEGA 7 through the neighbor-joining method (bootstrap value of 1000). **(B)** and **(E)** The gene structure of each gene is represented where green boxes indicated the exonic regions while black lines indicate the intronic parts. Further, conserved motifs in each sequence are shown **(C)** and **(F)** where specific color indicates a specific motif.

Histidine Kinases subgroup members either possess a simple histidine kinase domain or a CHASE domain associated with the HK domain, and the CHASE domain is crucial for proteins to recognize and bind cytokinin (He et al., 2016b). *Brassica oleracea* has four CHKs (BoHK2.1/2.2/3/4) and 5 HK (BoHK1/5.1/5.2, BoCKI1.1, and BoCKI1.2) proteins contain HK domain followed by HATPase_c domain (Figure 1; Supplementary Table S3). The CHKs have a CHASE domain followed by HK HATPase_c and Rec domains. Additional Transmembrane (TM) domains present in the CHK proteins have been shown to play an important role in membrane-associated signal transduction in plants (Dhar et al., 2019). A similar pattern for motif conservation was observed in these HKs. HKs contained six conserved motifs and CHKs also contained the same motifs with one additional motif (Figure 2C). Gene structure showed that these proteins contain six to thirteen introns. This conservation of gene structure and motif patterns suggests that they are structurally similar and potentially involved in similar biological functions such as signal transduction.

Ethylene receptors have a common domain structure containing GAF, HK, HATPase_c, and Rec domains. In *B. oleracea* six ethylene receptors were identified. BoETRs and BoERSs are representative of the ethylene receptor family with or without the Rec domain and are involved in ethylene response. The ETR1 proteins (BoETR1 and BoETR2) had all the standard domains present in ethylene receptors. The ERS proteins (BoERS2.1 and BoERS2.2) show quite a difference in the presence of domains. The general domain organization includes the GAF domain and lacks the Rec domain (Figure 1; Supplementary Table S3). Motif analysis showed that these proteins have at least five motifs conserved in them. Gene structure revealed that *BoETRs* contain two to five introns.

Phytochrome/photoreceptors are characterized by the presence of a chromophore binding group and two PAS domains that enable plants to respond to light stimuli and regulate the processes of growth and development. There are six members of the *PHY* subgroup in *B. oleracea*. Domain analysis showed that phytochromes contain a GAF domain, an N-terminal PHY domain (involved in light absorption), two PAS domains, and one His Kinase domain involved in signal transduction. The domain PHY, GAF, and PAS play important roles in responding to red light and far-red light signals during plant growth and development in *Arabidopsis* (Dhar et al., 2019). Like other HKs this subgroup also contained highly conserved motifs as well as gene structure. Gene structure showed that these contain two to five introns (Figure 2B).

#### 3.1.2 HP protein family in *Brassica oleracea*

Histidine-containing phosphotransmitters (HPs/Hpts) are the signal mediators in multistep His-to-Asp phosphorelay and originated in plants (Hwang et al., 2020). Eight *BoHP* genes were identified: seven authentic and one pseudo-Hpt. All BoTCS are small proteins with 145–156 amino acids. Each member contains a conserved phosphorylation motif (XHQXKGSSXS) with a conserved Histidine phosphorylation site (H residue). All the BoHPs contained this residue except BoHP6 in which H residue is replaced by N residue. Similarly, in AHP6 and APHP1, which are *Arabidopsis* pseudo-Hpts, their conserved His residue was replaced by N. These pseudo-Hpts are also sharing high similarity which suggests that they may share similar functionality. Same pattern was observed in cucumber and rice (Supplementary Table S2) (Du et al., 2007; He et al., 2016a). Gene structure revealed that each of the eight *BoHP* genes contains six to seven exons with five to six introns, respectively, except *BoHP6*. *BoHP6* contains five exons and four introns (Figure 2E). Motif analysis suggested that all members had almost four motifs (motifs 1, 2, 3, and 16) conserved in them. While the rest of the motifs had random distribution across the members (Figure 2F).

#### 3.1.3 RR protein family in *Brassica oleracea*

A total of 51 *RR* genes were identified in *B. oleracea* of which 18 are RR-Type-A, 16 RR-Type-B, 5 RR-Type-C, and 12 PRRs (Supplementary Table S4). The final response to the environment is provided by RRs and the RR family in *Arabidopsis* has been shown to contain conserved aspartic acid (D) and lysine (K) residue. All the sequences containing the Rec domain only are identified as RR-Type-A and contain conserved D-D-K motifs which is the final receiver of the phosphate group with highly conserved D-residue at N-terminal (Gahlaut et al., 2014).

RR-Type-B family members are different from RR-Type-A family members because they contain an additional DNA binding domain (MYB). It has been shown that RR-Type-B functions as a transcription factor. The 16 RR-Type-B family members were found in *S. bicolor* (Supplementary Table S2). They also contain a conserved N-terminal D residue which plays a key role in receiving the phosphate group to initiate downstream processes. Different from RR-Type-A, five identified RR-Type-C contained a Rec domain but lacked C-terminal extension. *Arabidopsis* has two members of the RR-Type-C family (ARR22 and ARR24). These are divergent from RR-Type-A and do not express during cytokinin response. *Brassica oleracea* was found to contain five members of this family (BoRR22.1, BoRR22.2, BoRR24.1, BoRR24.2, and BoRR24.3) having significant homologous relationships with their *Arabidopsis* counterparts. All these five proteins had three motifs conserved.

**FIGURE 3:**
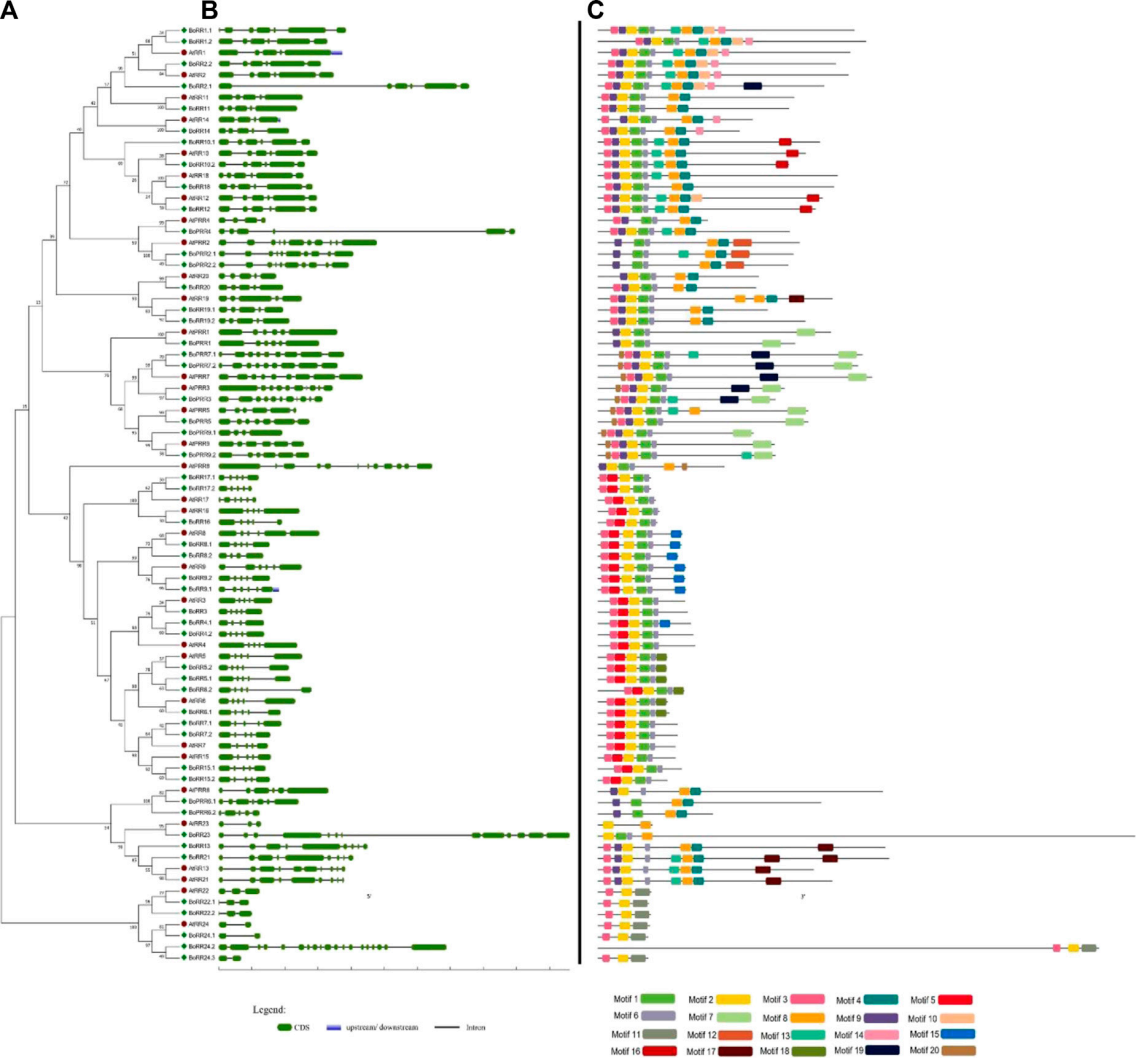
Gene structure and conserved motifs of RRs in *B. oleracea*. **(A)** The phylogenetic tree of all BoRRs was constructed using MEGA 7 through the neighbor-joining (NJ) method and bootstrap value of 1000. **(B)** This part represents the intronic and exonic parts of a specific gene sequence: the green color shows the exonic region whereas black lines show the intronic region. **(C)** Motifs are shown in specific colors and their presence in gene sequences indicates their conservation among sequences.

*Brassica oleracea* had 12 BoPRRs a conserved Rec domain and CCT domain, which play an important role in regulating circadian rhythms. These are further divided into type-B PRRs and Cloak-associated PRRs. Type-B PRRs have a Myb domain instead of a CCT motif and include BoPRR2.1/2.2 and BoPRR6.1. Cloak-associated PRRs have the conserved arginine and lysine in the CCT motif and include BoPRR1, BoPRR3, BoPRR5, BoPRR7.1, BoPRR7.2, and BoPRR9.1. All these *BoPRR* genes also have conserved gene structures and motifs (Figure 3).

### 3.2 Physiochemical characteristics of BoTCS proteins

The sequence characteristics of 80 BoTCS proteins were analyzed (Supplementary Table S4). The BoTCSs are present on nine chromosomes (Chr), Chr 1-9. The exon number ranged from 3 to 19. The amino acid length varied from 134 (BoRR24.3) to 1,327 (BoRR24.3) aa. Accordingly, the molecular weight (MW) ranged from 15,030.16 to 146,733.58. The ExPasy ProtParam tool showed that BoTCS proteins had a very different isoelectric point (pI) with a value ranging from 4.65 to 9.2. This suggests the acidic and basic nature of proteins. Instability index (II) ranged from 22.62 (stable) to 81.76 (unstable), aliphatic index (AI) value ranging from 61.38 to 116.92, and grand average of hydropathicity index (GRAVY) values from −0.741 to −0.08 which showed protein’s hydrophobic as well as hydrophilic nature. BoTCS proteins localize mostly in the plasma membrane, nucleus, cytoplasm, and mitochondria (Supplementary Table S4).

### 3.3 Comparative phylogenetic analysis of two-component system proteins in diverse plant species

**FIGURE 4:**
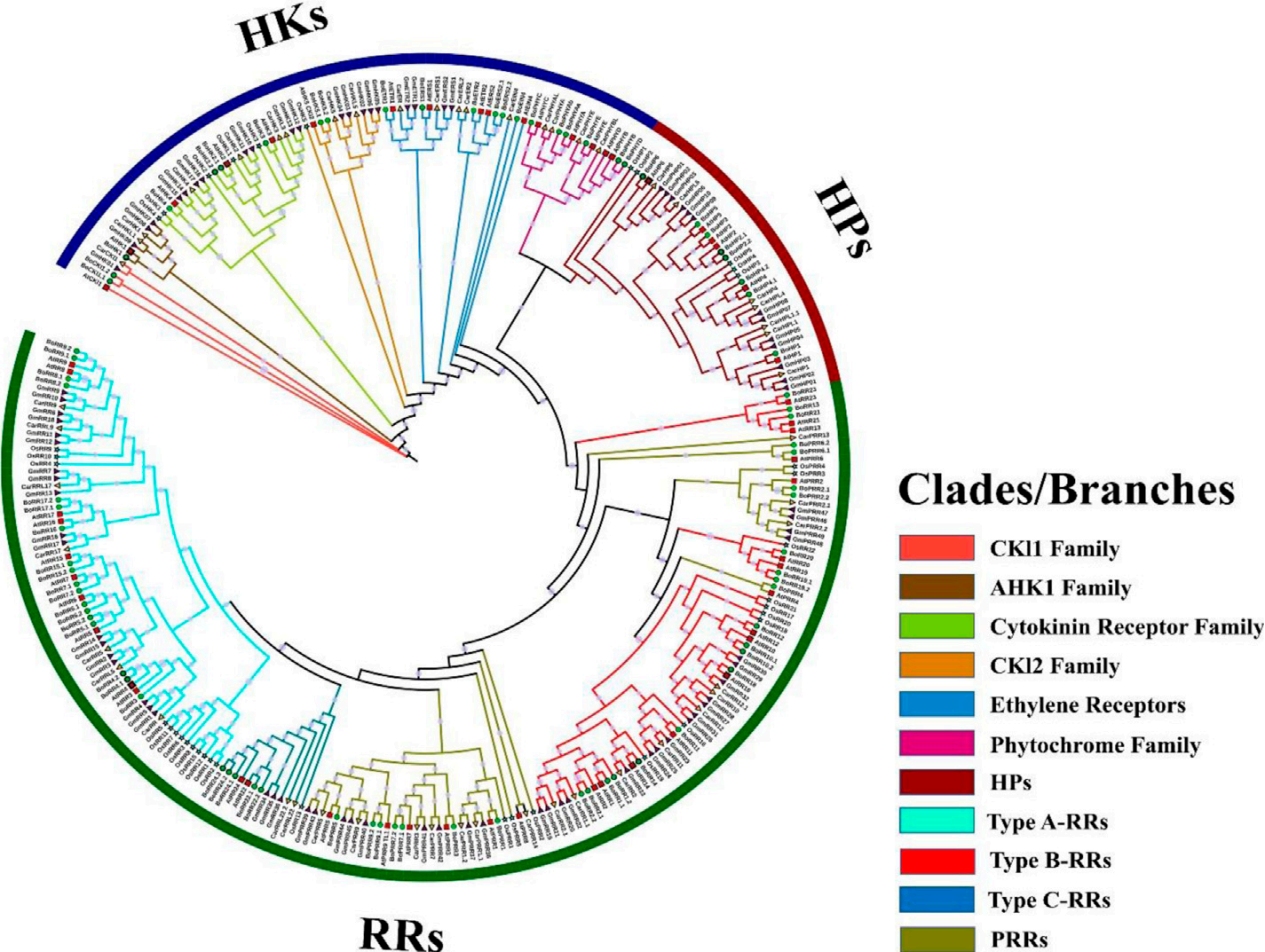
Comparative phylogenetic analysis of HKs, HPs, and RRs proteins present in *B. oleracea*, *A. thaliana*, *C. arietinum*, *O. sativa*, and *G. max*. The phylogenetic tree of all BoTCS members was constructed using IQ-Tree through a maximum-likelihood approach and bootstrap value of 1000. Three groups are further divided into subgroups. The clades and branches are given different colors. Each colored clade is representing a specific subgroup.

TCS genes in *B. oleracea*, *A. thaliana*, *C. arietinum*, *Oryza sativa,* and *Glycine* max have closer phylogenetic relationships. Based on these closer relationships, a phylogenetic and evolutionary analysis of five different plant species (mentioned above) was carried out (Figure 4). TCS gene family members were divided into three main families HKs, HPs, and RRs. HK family is further divided into five subgroups: CKI1 and AHK1, Cytokinin receptors, CKI2, ethylene receptors, and phytochromes. BoCKI1.1, BoCKI1.2, and BoHK1 are the members of the CKI1 and AHK1 subgroups which are orthologues of AtCKI1 and AHK1. AHK1 protein is involved in osmosensation and primarily expresses in the *A. thaliana* roots during salt stress (Cheng & Kieber, 2014). Cytokinin receptor subfamily has four members in *B. oleracea* while three members (BoHK2.1, BoHK2.2, BoHK3, and BoHK4) were present in *A. thaliana* (AHK2, AHK3, and AHK4). In *A. thaliana* this subfamily controls various cytokinin-regulated responses like seed size, cell division, leaf senescence, and other stress responses Mochida et al., 2010. Similarly, other plant species also have similar phylogenetic relationships with plants. These closer relationships among *B. oleracea*, their *A. thaliana* orthologues, and other plant members suggest that they may have a functional similarity. BoHK5.1 and BoHK5.2 belong to the CKI2 subfamily. Six members of the ethylene receptor subfamily are present in *B. oleracea* having homologous relationships with ETR1, ASR1, and EIN4. Since the phylogenetic analysis has shown homologous relationships among the TCS members, it suggests that they have similar functions in different plant species as they emerged from the same ancestral sequence (Figure 4).

Phytochromes or photoreceptors control growth and developmental process during light stress. Five members of this subfamily are present in *A. thaliana* while the current study has found six members in *S. bicolor*. Direct homologous relationships are found among all the members belonging to five plants. All the BoHPs are having close relationships with *A. thaliana*. This subfamily has six and eight members in *A. thaliana* and *B. oleracea*, respectively.

RRs from five plants were used to perform phylogenetic analysis and are classified as true RRs and pseudo-RRs (PRRs). True RRs are divided into further subgroups: RR-Type-A, RR-Type-B, and RR-Type-C. RR-Type-B is further classified as Type-B1, Type-B2, and Type-B3 RRs. All these BoRRs are closely related to their *A. thaliana* homologs. RRs give final responses to the environmental factors. RR-Type-B and RR-Type-C are mostly involved in cytokinin activities whereas RR-Type-A is found exclusively in land plants and is considered new members because of their absence in unicellular alga. PRRs are further classified into two subgroups: Clock-associated PRRs and Type-b PRRs based on their domain organization. Both subgroups contain seven and 5 members respectively.

### 3.4 Promoter analysis of *BoTCS* genes

The promoter sequences of the *BoTCS* genes were analyzed to predict the cis-regulatory elements to understand their functional as well as transcriptional roles. Several abiotic stress and hormone-related cis-regulatory elements have been found.

**FIGURE 5:**
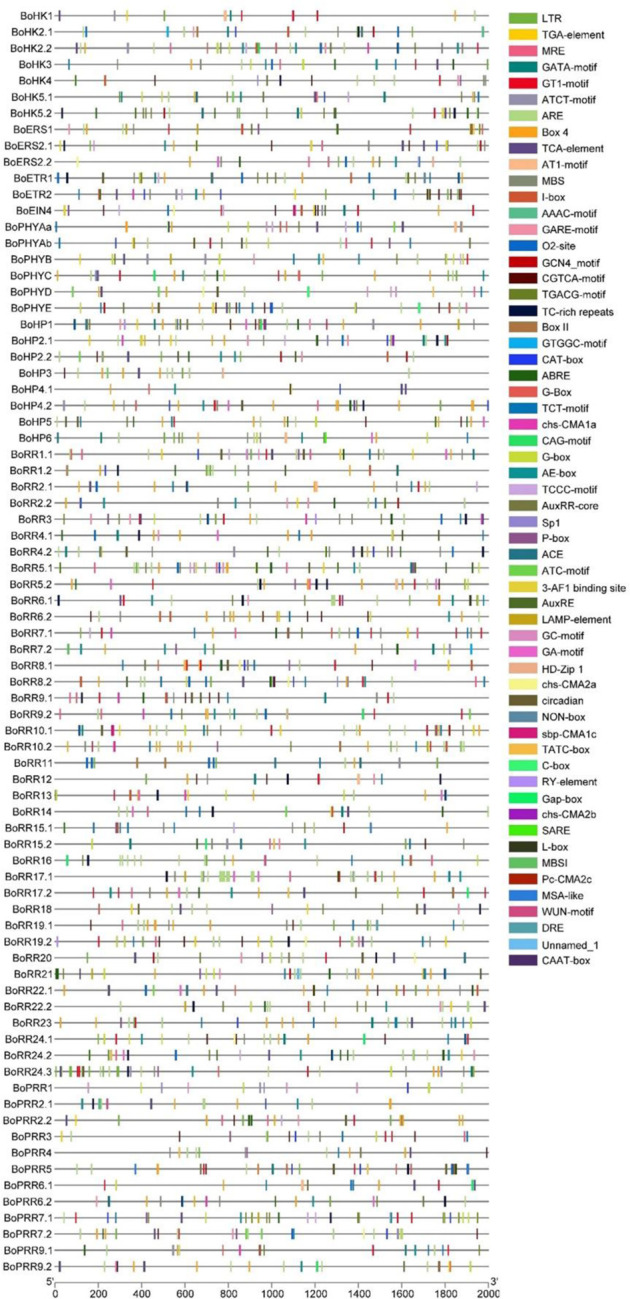
*cis*-regulatory elements in the *B. oleracea* TCS gene promoters, the1kb Region upstream the start codon. Each *cis*-element is given a specific color which indicates whether or not it is present in the gene promoter sequence.

Major classes of cis-elements found include Stress-responsive (low temperature, light), MYB-binding sites, and hormone-responsive elements. The element involved in low-temperature stress (LTR: CCGAAA), light-responsive element (GATA motif: GATAGGG, Box II: TGGTAATAA, LAMP element: CTTTATCA), gibberellin-responsive element (GARE-motif: TCTGTTG), abscisic acid-responsive element (ABRE: ACGTG), element involved in salicylic acid response (TCA-element: CCATCTTTTT), MYB binding site (MRE: AACCTAA), cis-regulatory element involved in circadian control (circadian: CAAAGATATC), MeJa-responsive element (CGTCA-motif: CGTCA) and auxin-responsive element (TGA-element: AACGAC) were common in almost all *BoTCS* genes (Figure 5; Supplementary Table S5). All these elements induce a response to abiotic stress and growth hormone regulation. An element for anaerobic induction (ARE) is also found. These results imply that TCS family members of *B. oleracea* have cis-acting elements in their promoter sequence and hence can play pivotal roles in abiotic stress tolerance. Most of these *cis*-elements were associated with shade stress which further led to the involvement and regulation of these genes in shade stress. Most importantly, *BoPHYs, BoERS1.1, BoERS2.1, BoERS2.2, BoRR10.2,* and *BoRR7.1* contained shade-stress related elements which can be used for shade stress tolerant breedings.

### 3.5 Chromosomal localization of *BoTCS* genes

To better understand the genomic distribution of *BoTCS* genes, their location on chromosomes and duplication events were predicted by performing syntenic analysis. The nine chromosomes had a different number of genes distributed on them (Supplementary Figure S2). The distribution of genes is extremely random, like chromosome 2 contains only six genes while chromosome 4 contains 12 genes. All *BoRRs* were distributed on every chromosome. Similarly, every gene family has its member on every chromosome.

Gene duplication events were also identified for this family. Gene duplication event is an important event in evolution; therefore, it led to analyze the evolution of these genes. Both tandem and segmental duplications were analyzed. The potential gene duplication events identified were 27. Among duplication types, tandem duplication was found in three pairs (*BoCKI1.1/BoCKl1.2, BoRR8.1/BoRR9.2,* and *BoRR15.1/BoRR15.2*). Rests of the pairs were segmentally duplicated. Synonymous rate (*Ks*), non-synonymous (*Ka*), and their ratio (*Ka/Ks*) were calculated (Table 1). Using these values, duplication time was also calculated. The *Ks* value for these duplications ranged from 0.18 to 0.8922. Therefore, the time of divergence ranged from 2.41 to 40.236 million years ago (mya).

**TABLE 1:** Calculation of *Ka*, *Ks*, and *Ka/Ks* values and divergence time of the duplicated *BoTCS* gene pairs.

Duplicated gene pairs	*Ka*	*Ks*	*Ka*/*Ks*	Time (MYA^a^)	Duplication type
*BoCKI1.1/BoCKl1.2*	0.1064	0.124	0.858065	4.133333333	Tandem
*BoERS1/BoETR1*	0.6122	1.4943	0.40969	49.81	Segmental
*BoERS2.1/BoERS2.2*	0.1048	0.1308	0.801223	4.36	Segmental
*BoPHYAa/BoPHYAb*	0.0704	0.4248	0.165725	14.16	Segmental
*BoPHYB/BoPHYD*	0.0767	0.0917	0.836423	3.056666667	Segmental
*BoHP2.1/BoHP2.2*	0.107	0.2665	0.401501	8.883333333	Segmental
*BoHP2.1/BoHP3*	0.1064	0.2726	0.390315	9.086666667	Segmental
*BoHP4.1/BoHP4.2*	0.1051	0.2858	0.36774	9.526666667	Segmental
*BoRR1.1/BoRR1.2*	0.1571	0.4618	0.340191	15.39333333	Segmental
*BoRR1.2/BoRR2.1*	0.5566	1.7376	0.320327	57.92	Segmental
*BoRR2.1/BoRR2.2*	0.576	0.6956	0.828062	23.18666667	Segmental
*BoRR3/BoRR4.2*	0.2012	0.1001	2.00999	3.336666667	Segmental
*BoRR6.1/BoRR6.2*	0.1163	0.0812	1.432266	2.706666667	Segmental
*BoRR7.1/BoRR7.2*	0.0675	0.1367	0.493782	4.556666667	Segmental
*BoRR7.2/BoRR15.2*	0.1805	0.1399	1.290207	4.663333333	Segmental
*BoRR8.1/BoRR9.1*	0.1531	0.0812	1.885468	2.706666667	Segmental
*BoRR8.1/BoRR9.2*	0.2305	0.0846	2.724586	2.82	Tandem
*BoRR8.2/BoRR9.1*	0.197	0.0723	2.724758	2.41	Segmental
*BoRR10.1/BoRR10.2*	0.159	0.7166	0.221881	23.88666667	Segmental
*BoRR10.1/BoRR12*	0.3779	0.824	0.458617	27.46666667	Segmental
*BoRR10.1/BoRR14*	0.8922	1.2071	0.739127	40.23666667	Segmental
*BoRR10.2/BoRR12*	0.2627	0.3342	0.786056	11.14	Segmental
*BoRR13/BoRR21*	0.1424	0.1337	1.065071	4.456666667	Segmental
*BoRR15.1/BoRR15.2*	0.18	0.1416	1.271186	4.72	Tandem
*BoRR22.1/BoRR22.2*	0.0629	0.4582	0.137276	15.27333333	Segmental
*BoPRR2.1/BoPRR2.2*	0.1844	0.4836	0.381307	16.12	Segmental
*BoPRR5/BoPRR9.1*	0.6968	0.8073	0.863124	26.91	Segmental

^a^MYA: million years ago.

### 3.6 MiRNA target prediction of *BoTCSs*

**FIGURE 6:**
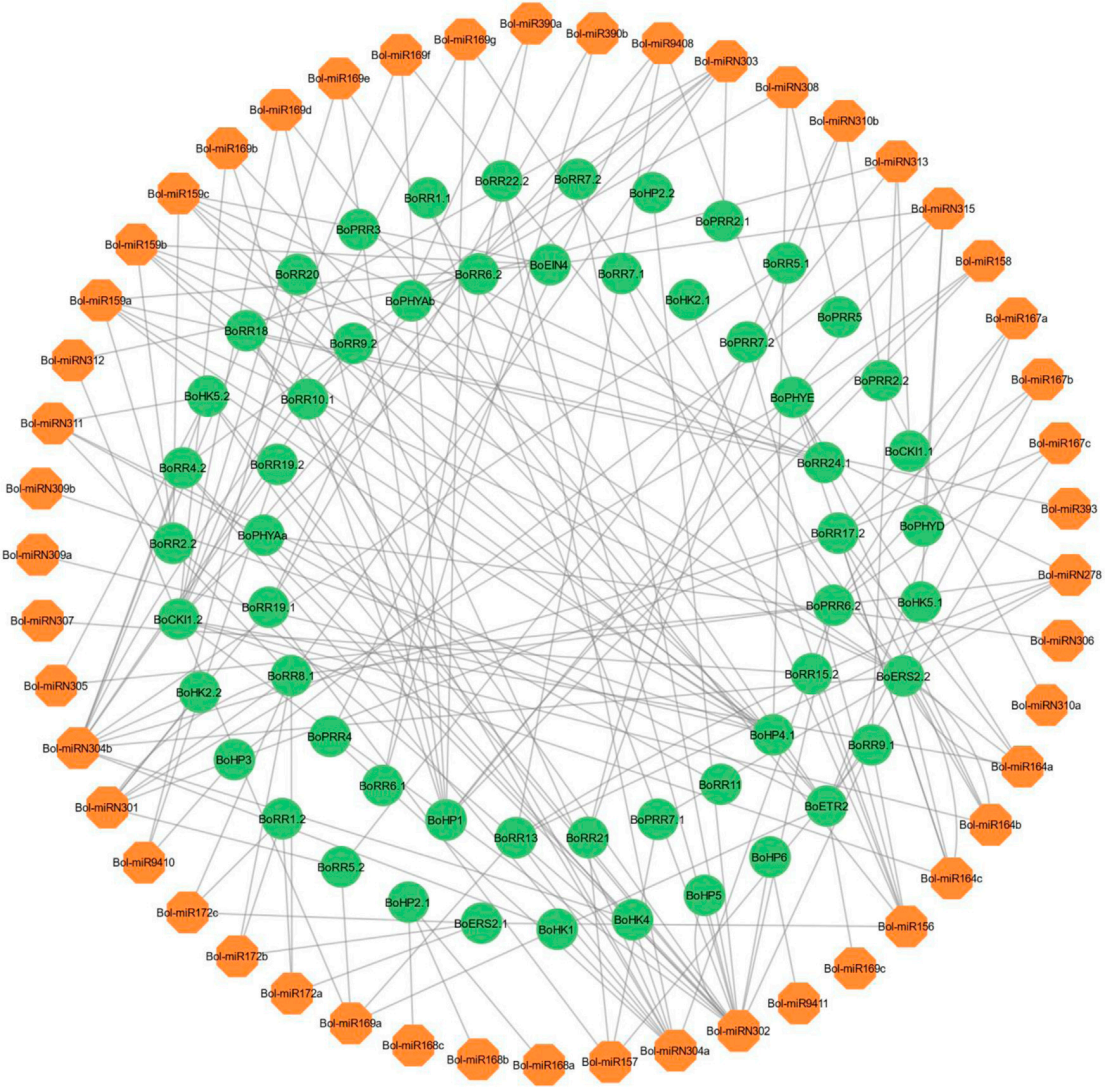
Network representation of regulatory association among miRNAs and *BoTCSs*. The network has been constructed by using Cytoscape. The miRNAs involved in regulating *BoTCSs* are in orange color. *BoTCS* genes are in green color and black colored lines represent the regulatory relationship.

A total of 186 miRNAs were identified that targeted 80 BoTCSs, with expectation values ranging from 3 to 5 (Figure 6; Supplementary Table S6). 13 miRNAs were targeting *BoCKI1.2*, 12 miRNAs were targeting *BoERS2.2*, while some other *BoTCSs* were targeted by less than 10 miRNAs. Among these miRNAs, most of them were responsible for inhibiting the cleavage of target transcript while only a few were involved in inhibiting the translation of target genes.

### 3.7 BoTCS protein’s structure prediction

A protein’s structural features indicate its potential interactions or binding with the other molecules as well as its functional correlations. In this study, the three-dimensional structures of 6 BoTCS proteins were predicted by using the I-TASSER server. These models had higher stereochemical characteristics locally as well as globally. In these models, the major structural component is found in the helix. The structural symmetry of proteins belonging to a particular family is similar. From the HK family, models of two proteins BoHK5.1 and BoETR2 are predicted. Different helices are shown in blue, green, yellow, and orange colors. Helices are the main structural component present in these proteins with fewer loops and sheets. From the HP family, BoHP1 and BoHP6 were modeled and helices mainly constitute these proteins. From the RR family, BoRR6.1 and BoRR9.2 had their structures modeled. These proteins have helices as well as loops (Supplementary Figure S3). This conservation of secondary structures among proteins suggests their similarity in functions as well.

### 3.8 Molecular docking analysis of BoTCS proteins with abscisic acid (ABA)

**FIGURE 7:**
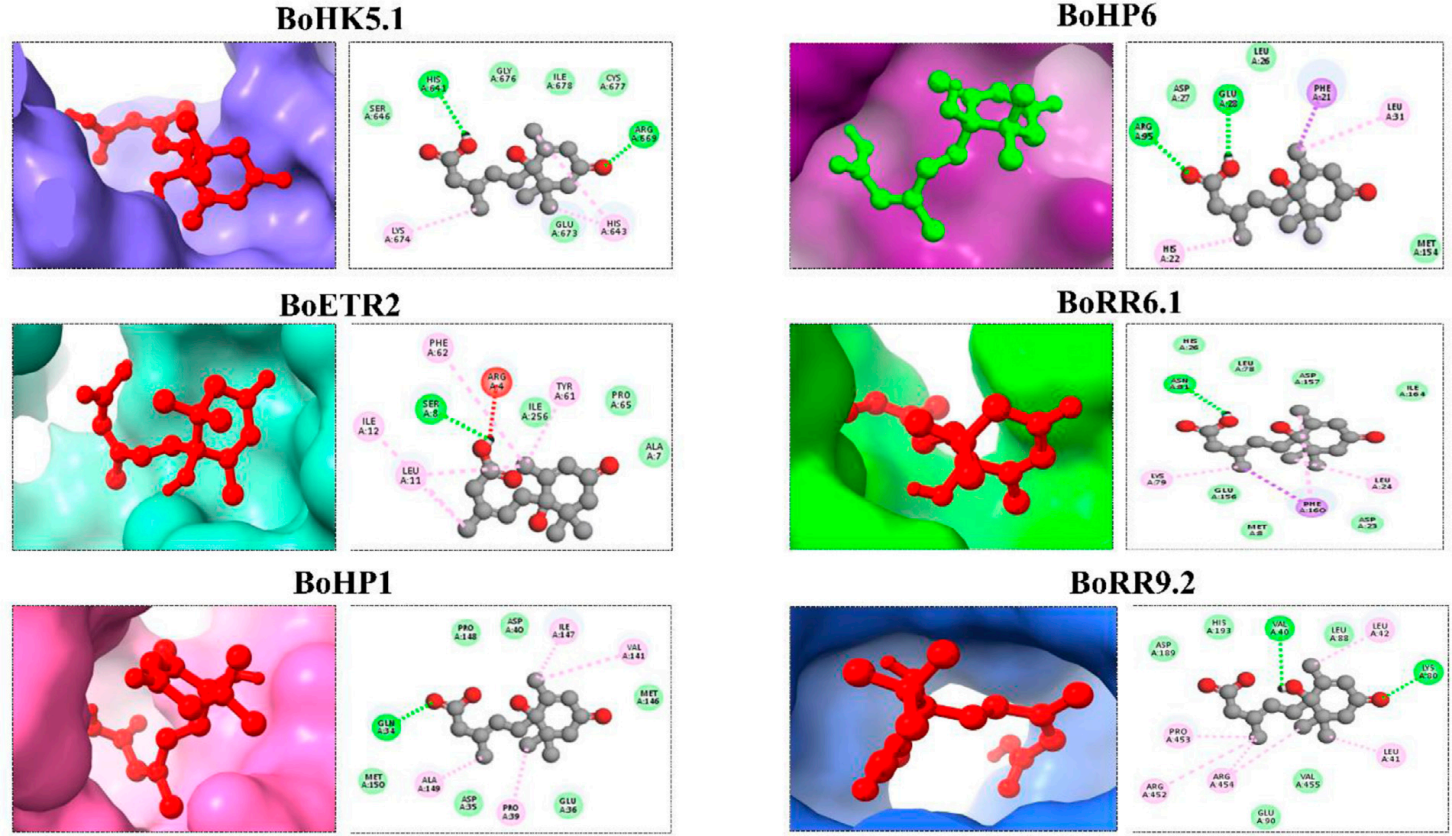
Molecular docking of Abscisic acid (ABA) against TCS proteins from *B. oleracea*. Different colored structures in the background are proteins bound with the ligand (ABA). Their 2D structures are shown to represent interacting molecules.

ABA was docked against 6 BoTCSs to check their binding because TCSs regulate a variety of biological activities and responses to environmental stimuli (Figure 7). The cytokinin-responsive TCS controls ABA and osmotic stress responses (Tran et al., 2010).

Docking complexes have −6.1 Kcal/Mol (BoRR9.2) to −5 Kcal/Mol (BoHP6) global energy. RMSD value for BoHK5.1 is 2.659 Å. Its conserved residues are HIS A:641, HIS A:643, ARG A:669, GLU A:673, and LYS A: 674 BoETR2 is having conserved residues, ARG A:4, ALA A:7, SER A:8, LEU A:11, ILE A:12, TYR A:61, and PHE A:62 with their RMSD value of 2.644 Å BoHP1 conserved residues are GLN A:34, PRO A:39, VAL A:141, ILE A:147, and ALA A:149 having an RMSD value of 2.216 Å BoHP6’s conserved residues include PHE A:21, HIS A:22, GLU A:28, LEU A:31, and ARG A:95 with an RMSD value of 2.82 Å BoRR6.1 has conserved residues including LEU A:24, LYS A:79, ASN A:81, and PHE A:160 RMSD value of 0.498 Å BoRR9.2 conserved residues include VAL A:40, LEU A: 41, LEU A: 42, LYS A:80, ARG A:452, PRO A:453, and ARG A:454 with RMSD value of 0.465 Å (Supplementary Table S4). All these binding affinities of the BoTCS proteins imply that they can bind with the ligand to perform specific functions.

### 3.9 Analysis of differently expressed genes in the TCS family of *Brassica oleracea*

**FIGURE 8:**
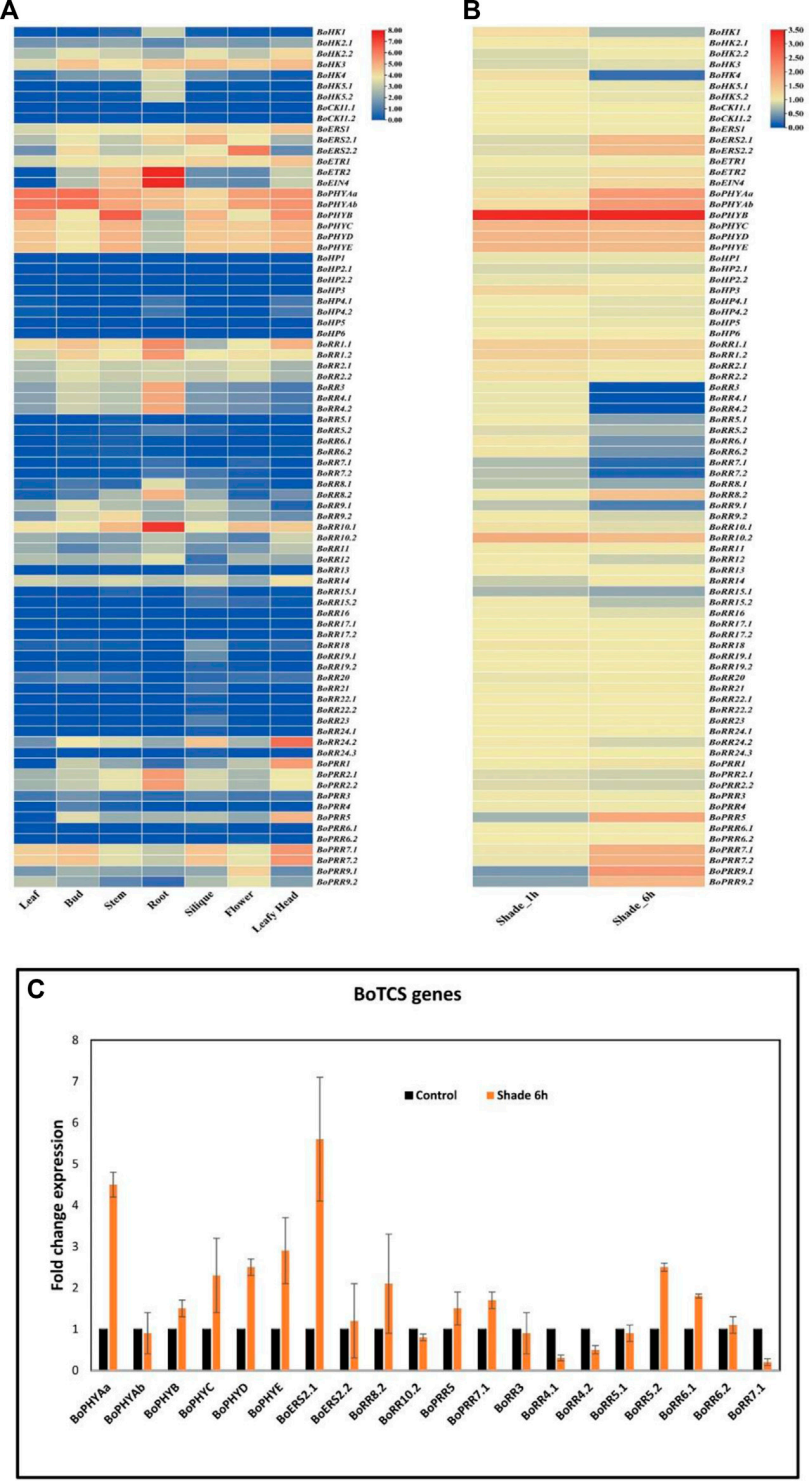
**(A)** Expression analysis of *BoTCS* gene family. Heatmap shows the expression profiles of the genes in the leaf, bud, stem, root, siliques, flower, and leafy head. **(B)** Expression analysis of *BoTCSs* in shade for 1 h and 6 h. For each gene, the relative FPKM value was calculated and the log 2 (fold change) scaled expression FPKM values were used to evaluate the expression level. The mean of three biological replicates is calculated for each specific tissue. The level of relative decreased expression (blue) or increased expression (red) is shown. **(C)** Expression validation of *BoTCS* genes in control light (black) and shade (brown) stress. Vertical lines are representing the standard deviation of expression value from the mean point.

According to previous studies and studies of cis-regulatory elements, it is found that TCS genes can play a key role in abiotic stress response. In the current study, the expression of BoTCS genes was investigated using RNA-seq data to understand the tissue-specific and stress-responsive (light/shade) expression of these genes. The expression of *BoTCS* genes was estimated in different tissues including leaf, bud, stem, root, flower, and leafy head. The majority of TCS genes were not expressed or weakly expressed in all tissues. However, members of the PHY subfamily were mostly expressed in all tissues. Similarly, *BoETR2, BoEIN4,* and *BoRR10.1* were highly expressed in roots (Figure 8A). In stress response (shade stress for 1 and 6 h) majority of TCS genes were expressed (Figure 8B). Members of the PHY family were mostly highly upregulated in response to shade. In response to shade stress of 6 h, *BoPHYAa*, *BoPHYAb*, *BoPHYB*, *BoPHYC*, *BoPHYD*, *BoPHYE*, *BoERS2.1*, *BoERS2.2, BoRR8.2, BoRR10.2, BoPRR5, BoPRR7.1, BoPRR7.2, BoPRR9.1,* and *BoPRR9.2* were upregulated and *BoRR3, BoRR4.1, BoRR4.2, BoRR5.1, BoRR5.2, BoRR6.1, BoRR6.2, BoRR7.1, BoRR7.2, BoRR8.1, BoRR9.*1 were downregulated (Figure 7B). To validate the expression level of stress-responsive BoTCS genes, real-time RT-qPCR was performed. It was observed that most of the genes demonstrated expression patterns similar to RNA-seq data except *BoPHYAb, BoERS2.2, BoRR8.2, BoRR10.2, BoRR5.2,* and *BoRR6.1* (Figure 8C).

## 4 Discussion

Cytokinins are the key plant hormones that regulate various processes in plants including germination, apical dominance, development of flower and fruit, pathogen interaction of plants, and cell division of several tissues. Signal transduction of cytokinin occurs using a phosphor-relay which is derived from TCS (L. He et al., 2020). Phosphorylation of proteins is one of the most common methods for intracellular signaling where an outside stimuli is received and processed to generate the required response. Protein kinases use ATP as the phosphate donor to phosphorylate their substrates (Sharan et al., 2017).

In this study, the main objective was to find a Two-Component System (TCS) in *B. oleracea*, its involvement in light/shade stress, and how they express in multiple tissues. To accomplish this objective, a comprehensive genomic analysis was carried out in *B. oleracea* which identified 21 HKs, 8 HPs, and 51 RRs. In Arabidopsis a number of these genes identified were as follows: 17HKs, 6 HPs, and 33 RRs (Cheng & Kieber, 2014). Similarly in *S. bicolor,* 13HKs*,*5 HPs, and 19RRs were identified (Zameer et al., 2021) In melon 51 members of this family were identified with no identified Type-C RR*.* All these differences in gene number across species is attributed to the duplication events. Type-C RR is thought to be the earliest RR and changes in its promoters may have led to the emergence of Type-A RRs (Pils & Heyl, 2009).

The phylogenetic study showed that *B. oleracea* TCS proteins are divided into three Groups: BoHKs, BoHPs, and BoRRs. These groups were further divided into subfamilies. A similar grouping was found in Arabidopsis and rice (Sharan et al., 2017). On the phylogenetic tree, these subfamilies were dispersed and proteins belonging to the same subfamilies were clustered together. HKs were further grouped into CKI1 and CKI2 according to their domains. CKI1 contained AHK1 and CKI2 group contained true histidine kinase BoHK5 (Dhar et al., 2019). The presence of the CHASE domain makes identification of the clade regarding cytokinin receptors. RRs subfamilies cluster as a larger clade in the phylogenetic tree which further subdivides the RRs according to their specific clades (Wallmeroth et al., 2019).

The numbers of genes and phylogenetic analysis show that different plants have a different number of genes belonging to the same superfamily. Various events like gene duplication as well as whole genome duplication can lead to the difference in the number of genes in multiple plants. Duplication studies revealed that mainly segmental duplication led to the expansion of this gene family in *S. bicolor*. Segmental duplication was the main mechanism of duplication in *A. thaliana*, *Brassica rapa* (Chinese cabbage), and *C. arietinum*. Although tandem duplication is also found but mainly the genes are segmentally duplicated. In tomato, both events have been identified. This shows the role of duplication in the expansion of this gene family in plants.

In the promoter region of *BoTCS* genes, several cis-acting elements for the regulation of abiotic stress responses are found. These include low-temperature responsive, light responsive, drought-inducibility, and hormone-responsive elements (auxin, gibberellins, salicylic acid, and MeJa responsive elements). Similar results were observed for *S. bicolor* genes which contained elements for light, drought, and hormone responsiveness (Zameer et al., 2021), Similarly, in watermelon and cucumber, drought-responsive elements, abscisic acid-responsive and elements for anaerobic induction were found (He et al., 2016a). In banana, several hormone-responsive elements were found abundantly. This shows that TCS-mediated signaling is induced by hormones, developmental conditions, and various stresses. In plants, complementary miRNAs and the target genes control various developmental, metabolic, and stress-related responses. Similarly, for *BoTCSs*, predicted associated miRNAs were mostly responsible for inhibiting the cleavage of target transcripts. Only a few of these predicted miRNAs were involved in the inhibition of the target gene’s translation.

Expression analysis demonstrated that several genes express in the root, stem, bud, leaves, and leafy head and under shade/light treatment (Figure 7). In tomatoes, 12 genes from HK and RR families were found to express highly in roots which is the main site for the synthesis of cytokinins (He et al., 2016b). These genes are likely to be involved in cytokinin-mediated signal transduction in *Arabidopsis* as well its homologs (Schaller et al., 2008). In *S. bicolor*, the expression was showed by ethylene receptors and RRs mainly. In tomatoes, similar proteins HKl especially ethylene receptors and PRRs were expressed during the ripening of the fruit. It is been found that these genes are involved in the development and ripening of fruits (Tieman et al., 2000; Gupta et al., 2014). Most of the genes in Chinese cabbage and soybean responded to drought and salt stresses. Most of the tomato genes were expressed under drought stress while *Arabidopsis* genes show a decline in expression under drought stress (Nguyen et al., 2016).

This expression profiling of TCS is important for understanding the function of these genes under different abiotic stresses. Cis-regulatory elements for abiotic stresses are found in all subfamilies of *BoTCS,* especially RRs. Similarly, most of the genes from the RR subfamily are found to have higher expression in multiple tissues as well as in shade stress. This reveals the involvement of *BoTCSs* especially BoRRs in abiotic stress tolerance. In *Medicago truncatula* few TCS genes were highly expressed in different organs suggesting their tissue-specific role (Tan et al., 2019). In chickpea, RRs were localized in the nucleus and interacted with the promoters of nodulation genes which indicate that they act as a transcription factor and regulate the early stages of nodulation (Varshney et al., 2013). In *Arabidopsis*, HKs and the type-A RRs that induce cold stress had a negative role in cold stress through inhibition of ABA response (Jeon et al., 2010). In *B. rapa*, gene expression regarding high salinity and drought stress was found. Ethylene receptors are involved in pollen thermotolerance in tomatoes. Similarly, TCS genes express under dehydration stress in *G. max*. GATA-motif and Protein binding element in the promoter region of TCS genes were identified which is consistent with the result of previous studies (Taquet et al., 2001; Fusada et al., 2005). These studies show the evolution of the TCS gene family in *B. oleracea* and how this evolution led to the expansion of the genes as well as their functionality. These studies require further biotechnological analyses, however, all the information gained through this study is valuable for the future studies on the functional insights of the *B. oleracea* under environmental stresses. The genes showing distinct expression pattern can be used in the development of genetically modified crops which will not only be resistant to the stresses but also provide an increase in crop yield.

## 5 Conclusion

In *B. oleracea*, 80 genes have been identified as potential members of the TCS gene family. HKs accounted for 21 of the 80 identified genes, whereas HPs and RRs accounted for 8 and 51 genes respectively. Moreover, further information regarding conserved domains, motifs, cis-regulatory elements, phylogenetic analysis, and protein structure prediction showed the conservation of the TCS gene family in diverse plant species. Gene duplication events for maximum genes were identified and mainly the segmental duplication caused the expansion of these genes. MiRNAs also showed associations with certain *BoTCS* genes. The genes showing unique expression (higher or lower) in light/shade stress are ideal candidates for gene manipulation and characterization in breeding for shade tolerance. The qRT-PCR results verified the expression variation of genes including *BoERS1*, *BoERS2.1*, *BoERS2.2*, *BoRR10.2*, *BoRR7.1,* and *BoPHYs*. This would help to acquire a better understanding of TCS and further genomic research may help to increase plant resistance to abiotic stresses.

## Data Availability

The original contributions presented in the study are included in the article/Supplementary Material, further inquiries can be directed to the corresponding authors.

## References

[B1] Ahmad B. Azeem F. Ali M. A. Nawaz M. A. Nadeem H. Abbas A. (2020). Genome-wide identification and expression analysis of two component system genes in Cicer arietinum. Genomics 112 (2), 1371–1383. 10.1016/j.ygeno.2019.08.006 31415811

[B2] Arapitsas P. Sjöberg P. J. R. Turner C. (2008). Characterisation of anthocyanins in red cabbage using high resolution liquid chromatography coupled with photodiode array detection and electrospray ionization-linear ion trap mass spectrometry. Food Chem. 109 (1), 219–226. 10.1016/j.foodchem.2007.12.030 26054284

[B3] Asakura Y. Hagino T. Ohta Y. Aoki K. Yonekura-Sakakibara K. Deji A. (2003). Molecular characterization of His-Asp phosphorelay signaling factors in maize leaves: Implications of the signal divergence by cytokinin-inducible response regulators in the cytosol and the nuclei. Plant Mol. Biol. 52 (2), 331–341. 10.1023/A:1023971315108 12856940

[B4] Azeem F. Zameer R. Rehman Rashid M. A. Rasul I. Ul-Allah S. Siddique M. H. (2022). Genome-wide analysis of potassium transport genes in Gossypium raimondii suggest a role of GrHAK/KUP/KT8, GrAKT2.1 and GrAKT1.1 in response to abiotic stress. Plant Physiology Biochem. 170, 110–122. 10.1016/j.plaphy.2021.11.038 34864561

[B5] Bailey T. L. Johnson J. Grant C. E. Noble W. S. (2015). The MEME suite. Nucleic Acids Res. 43, W39–W49. 10.1093/nar/gkv416 25953851 PMC4489269

[B6] Bateman A. Coin L. Durbin R. Finn R. D. Hollich V. Griffiths‐Jones S. (2004). The Pfam protein families database. Nucleic Acids Res. 32, D138–D141. 10.1093/nar/gkh121 14681378 PMC308855

[B7] Brown G. R. Hem V. Katz K. S. Ovetsky M. Wallin C. Ermolaeva O. (2015). Gene: A gene-centered information resource at NCBI. Nucleic Acids Res. 43, 36–42. 10.1093/nar/gku1055 PMC4383897 25355515

[B8] Chen C. Xia R. Chen H. He Y. (2018). “TBtools, a Toolkit for Biologists integrating various HTS-data handling tools with a user-friendly interface,”. bioRxiv.

[B9] Cheng C. Y. Kieber J. J. (2014). Cytokinin signaling in plants. Mol. Biol., 269–289. 10.1007/978-1-4614-7570-5_14

[B10] Colling J. Pollier J. Makunga N. P. Goossens A. (2013). Jasmonate Signal. 1011, 305–315. 10.1007/978-1-62703-414-2 23616005

[B11] Dai X. Zhuang Z. Zhao P. X. (2018). psRNATarget: a plant small RNA target analysis server (2017 release). Nucleic Acids Res. 46 (W1), W49–W54. 10.1093/nar/gky316 29718424 PMC6030838

[B12] Dhar Y. V. Lakhwani D. Pandey A. Singh S. Trivedi P. K. Asif M. H. (2019). Genome-wide identification and interactome analysis of members of two-component system in Banana. BMC Genomics 20 (1), 674–715. 10.1186/s12864-019-6050-1 31455217 PMC6712864

[B13] Du L. Jiao F. Chu J. Jin G. Chen M. Wu P. (2007). The two-component signal system in rice (Oryza sativa L): A genome-wide study of cytokinin signal perception and transduction. Genomics 89 (6), 697–707. 10.1016/j.ygeno.2007.02.001 17408920

[B14] Elshemey W. M. Elfiky A. A. Gawad W. A. (2010). Correlation to protein conformation of wide-angle X-ray scatter parameters. Protein J. 29 (8), 545–550. 10.1007/s10930-010-9291-z 21046443

[B15] Fusada N. Masuda T. Kuroda H. Shimada H. Ohta H. Takamiya K. I. (2005). Identification of a novel cis-element exhibiting cytokinin-dependent protein binding *in vitro* in the 5′-region of NADPH-protochlorophyllide oxidoreductase gene in cucumber. Plant Mol. Biol. 59 (4), 631–645. 10.1007/s11103-005-0579-x 16244912

[B16] Gahlaut V. Mathur S. Dhariwal R. Khurana J. P. Tyagi A. K. Balyan H. S. (2014). A multi-step phosphorelay two-component system impacts on tolerance against dehydration stress in common wheat. Funct. Integr. Genomics 14 (4), 707–716. 10.1007/s10142-014-0398-8 25228409

[B17] Gasteiger E. Hoogland C. Gattiker A. Wilkins M. R. Appel R. D. Bairoch A. (2005). Protein identification and analysis tools on the ExPASy server. Proteomics Protoc. Handb., 571–607. 10.1385/1-59259-890-0:571

[B18] Guo Z. Kuang Z. Wang Y. Zhao Y. Tao Y. Cheng C. (2020). PmiREN: A comprehensive encyclopedia of plant miRNAs. Nucleic Acids Res. 48 (D1), D1114–D1121. 10.1093/nar/gkz894 31602478 PMC6943064

[B19] Gupta S. K. Sharma S. Santisree P. Kilambi H. V. Appenroth K. Sreelakshmi Y. (2014). Complex and shifting interactions of phytochromes regulate fruit development in tomato. Plant, Cell Environ. 37 (7), 1688–1702. 10.1111/pce.12279 24433205

[B20] Halawa M. Cortleven A. Schmülling T. Heyl A. (2021). Characterization of CHARK, an unusual cytokinin receptor of rice. Sci. Rep. 11 (1), 1722–1810. 10.1038/s41598-020-80223-2 33462253 PMC7814049

[B21] He L. Zhang F. Wu X. Hu Y. Dong L. Dewitte W. (2020). Genome‐wide characterization and expression of two‐component system genes in cytokinin‐regulated gall formation in zizania latifolia. Plants 9 (11), 1409–1426. 10.3390/plants9111409 33105697 PMC7690396

[B22] He Y. Liu X. Ye L. Pan C. Chen L. Zou T. (2016a). Genome-wide identification and expression analysis of two-component system genes in tomato. Int. J. Mol. Sci. 17 (8), 1204. 10.3390/ijms17081204 27472316 PMC5000602

[B23] He Y. Liu X. Zou T. Pan C. Qin L. Chen L. (2016b). Genome-Wide identification of two-component system genes in cucurbitaceae crops and expression profiling analyses in Cucumber. Front. Plant Sci. 7, 899–918. 10.3389/fpls.2016.00899 27446129 PMC4916222

[B24] Hu B. Jin J. Guo A. Y. Zhang H. Luo J. Gao G. (2015). Gsds 2.0: An upgraded gene feature visualization server. Bioinformatics 31 (8), 1296–1297. 10.1093/bioinformatics/btu817 25504850 PMC4393523

[B25] Hunter T. (1991). 4 protein kinases and phosphorylation site sequences. 200.

[B26] Hwang I. Chen H. Sheen J. (2020). Two-component signal transduction pathways in Arabidopsis. Plant Physiol. 129, 500–515. 10.1104/pp.005504 PMC161668 12068096

[B27] Jenuth J. P. (2000). The NCBI. Publicly available tools and resources on the Web. Methods Mol. Biol. Clift. N.J.) 132, 301–312. 10.1385/1-59259-192-2:301 10547843

[B28] Jeon J. Kim N. Y. Kim S. Kang N. Y. Novák O. Ku S. J. (2010). A subset of cytokinin two-component signaling system plays a role in cold temperature stress response in Arabidopsis. J. Biol. Chem. 285 (30), 23371–23386. 10.1074/jbc.M109.096644 20463025 PMC2906329

[B29] Kibbe W. A. (2007). OligoCalc: An online oligonucleotide properties calculator. Nucleic Acids Res. 35, W43–W46. 10.1093/nar/gkm234 17452344 PMC1933198

[B30] Kohl M. Wiese S. Warscheid B. (2011). “Cytoscape: Software for visualization and analysis of biological networks,” in Data mining in proteomics (Germany: Springer). 10.1007/978-1-60761-987-1_18 21063955

[B31] Le D. T. Nishiyama R. Watanabe Y. Mochida K. Yamaguchi-Shinozaki K. Shinozaki K. (2011). Genome-wide expression profiling of soybean two-component system genes in soybean root and shoot tissues under dehydration stress. DNA Res. 18 (1), 17–29. 10.1093/dnares/dsq032 21208938 PMC3041507

[B32] Letunic I. Bork P. (2021). Interactive tree of Life (iTOL) v5: An online tool for phylogenetic tree display and annotation. Nucleic Acids Res. 49, W293–W296. 10.1093/nar/gkab301 33885785 PMC8265157

[B33] Liu P. Wang S. Wang X. Yang X. Li Q. Wang C. (2020). Genome-wide characterization of two-component system (TCS) genes in melon (Cucumis melo L). Plant Physiology Biochem. 151, 197–213. 10.1016/j.plaphy.2020.03.017 32229405

[B34] Liu Z. Zhang M. Kong L. Lv Y. Zou M. Lu G. (2014). Genome-wide identification, phylogeny, duplication, and expression analyses of two-component system genes in Chinese cabbage (Brassica rapa ssp. pekinensis). DNA Res. 21 (4), 379–396. 10.1093/dnares/dsu004 24585003 PMC4131832

[B35] Mochida K. Yoshida T. Sakurai T. Yamaguchi-Shinozaki K. Shinozaki K. Tran L. S. P. (2010). Genome-wide analysis of two-component systems and prediction of stress-responsive two-component system members in soybean. DNA Res. 17 (5), 303–324. 10.1093/dnares/dsq021 20817745 PMC2955714

[B36] Nakashima K. Yamaguchi-Shinozaki K. (2013). ABA signaling in stress-response and seed development. Plant Cell Rep. 32 (7), 959–970. 10.1007/s00299-013-1418-1 23535869

[B37] Nguyen K. H. Ha C. V Nishiyama R. Watanabe Y. Leyva-González M. A. Fujita Y. (2016). Arabidopsis type B cytokinin response regulators ARR1, ARR10, and ARR12 negatively regulate plant responses to drought. Proc. Natl. Acad. Sci. U. S. A. 113 (11), 3090–3095. 10.1073/pnas.1600399113 26884175 PMC4801291

[B38] Orlek A. Phan H. Sheppard A. E. Doumith M. Ellington M. Peto T. (2017). A curated dataset of complete Enterobacteriaceae plasmids compiled from the NCBI nucleotide database. Data Brief 12, 423–426. 10.1016/j.dib.2017.04.024 28516137 PMC5426034

[B39] Pettersen E. F. Goddard T. D. Huang C. C. Couch G. S. Greenblatt D. M. Meng E. C. (2004). UCSF Chimera - a visualization system for exploratory research and analysis. J. Comput. Chem. 25 (13), 1605–1612. 10.1002/jcc.20084 15264254

[B40] Pils B. Heyl A. (2009). Unraveling the evolution of cytokinin signaling. Plant Physiol. 151 (2), 782–791. 10.1104/pp.109.139188 19675156 PMC2754637

[B41] Rombauts S. Déhais P. Van Montagu M. Rouzé P. (1999). PlantCARE, a plant cis-acting regulatory element database. Nucleic Acids Res. 27 (1), 295–296. 10.1093/nar/27.1.295 9847207 PMC148162

[B42] Rozas J. Ferrer-Mata A. Sanchez-DelBarrio J. C. Guirao-Rico S. Librado P. Ramos-Onsins S. E. (2017). DnaSP 6: DNA sequence polymorphism analysis of large data sets. Mol. Biol. Evol. 34 (12), 3299–3302. 10.1093/molbev/msx248 29029172

[B43] Sanchez G. (2013). Las instituciones de ciencia y tecnología en los procesos de aprendizaje de la producción agroalimentaria en Argentina. El Sist. Argent. Innovación Inst. Empres. Redes. El Desafío La Creación Apropiación Conoc. 651, 659–664. 10.1002/prot

[B44] Schaller G. E. Kieber J. J. Shiu S.-H. (2008). Two-component signaling elements and histidyl-aspartyl phosphorelays. Arabidopsis Book 6, e0112. 10.1199/tab.0112 22303237 PMC3243373

[B45] Schultz J. Copley R. R. Doerks T. Ponting C. P. Bork P. (2000). Smart: A web-based tool for the study of genetically mobile domains. Nucleic Acids Res. 28 (1), 231–234. 10.1093/nar/28.1.231 10592234 PMC102444

[B46] Shahriari A. G. Tahmasebi A. (2018). Evaluation of different RNA extraction methods from agropatch suppressor assay for small quantities of plant tissue and their application for analysis of gene expression. Not. Sci. Biol. 10 (3), 348–353. 10.15835/nsb10310307

[B47] Sharan A. Soni P. Nongpiur R. C. Singla-Pareek S. L. Pareek A. (2017). Mapping the “Two-component system” network in rice. Sci. Rep. 7 (1), 9287–9313. 10.1038/s41598-017-08076-w 28839155 PMC5571105

[B48] Tan S. Debellé F. Gamas P. Frugier F. Brault M. (2019). Diversification of cytokinin phosphotransfer signaling genes in Medicago truncatula and other legume genomes. BMC Genomics 20 (1), 373–419. 10.1186/s12864-019-5724-z 31088345 PMC6518804

[B49] Taquet P. Pale C. Biology E. Rhodin A. G. Miyata K. Sakai H. (2001). ARR1, a transcription factor for genes immediately responsive to cytokinins. Science 294, 1519–1521. 10.1126/science.1065201 11691951

[B50] Tieman D. M. Taylor M. G. Ciardi J. A. Klee H. J. (2000). The tomato ethylene receptors NR and LeETR4 are negative regulators of ethylene response and exhibit functional compensation within a multigene family. Proc. Natl. Acad. Sci. U. S. A. 97 (10), 5663–5668. 10.1073/pnas.090550597 10792050 PMC25885

[B51] Tran L. S. P. Shinozaki K. Yamaguchi-Shinozaki K. (2010). Role of cytokinin responsive two-component system in ABA and osmotic stress signalings. Plant Signal. Behav. 5 (2), 148–150. 10.4161/psb.5.2.10411 20023424 PMC2884120

[B52] Trifinopoulos J. Nguyen L. T. von Haeseler A. Minh B. Q. (2016). W-IQ-TREE: A fast online phylogenetic tool for maximum likelihood analysis. Nucleic Acids Res. 44, W232–W235. 10.1093/nar/gkw256 27084950 PMC4987875

[B53] Tsai Y. C. Weir N. R. Hill K. Zhang W. Kim H. J. Shiu S. H. (2012). Characterization of genes involved in cytokinin signaling and metabolism from rice. Plant Physiol. 158 (4), 1666–1684. 10.1104/pp.111.192765 22383541 PMC3320177

[B54] Varshney R. K. Song C. Saxena R. K. Azam S. Yu S. Sharpe A. G. (2013). Draft genome sequence of chickpea (Cicer arietinum) provides a resource for trait improvement. Nat. Biotechnol. 31 (3), 240–246. 10.1038/nbt.2491 23354103

[B55] Wallmeroth N. Jeschke D. Slane D. Nägele J. Veerabagu M. Mira-Rodado V. (2019). ARR22 overexpression can suppress plant two-component regulatory systems. PLoS ONE 14, e0212056. 10.1371/journal.pone.0212056 30742656 PMC6370222

[B56] Wei S. Xiao X. Wei L. Li L. Li G. Liu F. (2021). Development and comprehensive HS-SPME/GC–MS analysis optimization, comparison, and evaluation of different cabbage cultivars (*Brassica oleracea* L. var. capitata L) volatile components. Food Chem. 340, 128166. 10.1016/j.foodchem.2020.128166 33010642

[B57] Wohlbach D. J. Quirino B. F. Sussman M. R. (2008). Analysis of the Arabidopsis histidine kinase ATHK1 reveals a connection between vegetative osmotic stress sensing and seed maturation. Plant Cell 20 (4), 1101–1117. 10.1105/tpc.107.055871 18441212 PMC2390728

[B58] Yang J. Yan R. Roy A. Xu D. Poisson J. Zhang Y. (2015). The I-tasser suite: Protein structure and function prediction. Nat. Methods 12 (1), 7–8. 10.1038/nmeth.3213 PMC4428668 25549265

[B59] Ye J. Coulouris G. Zaretskaya I. Cutcutache I. Rozen S. Madden T. L. (2012). Primer-BLAST: A tool to design target-specific primers for polymerase chain reaction. BMC Bioinforma. 13, 134–211. 10.1186/1471-2105-13-134 PMC3412702 22708584

[B60] Yu C.-S. Lin C.-J. Hwang J.-K. (2004). Predicting subcellular localization of proteins for Gram-negative bacteria by support vector machines based on n -peptide compositions. Protein Sci. 13 (5), 1402–1406. 10.1110/ps.03479604 15096640 PMC2286765

[B61] Zameer R. Fatima K. Azeem F. Algwaiz H. I. M. Sadaqat M. Rasheed A. (2022). Genome-wide characterization of superoxide dismutase (SOD) genes in daucus carota: Novel insights into structure, expression, and binding interaction with hydrogen peroxide (H2O2) under abiotic stress condition. Front. Plant Sci. 13, 870241. 10.3389/fpls.2022.870241 35783965 PMC9246500

[B62] Zameer R. Sadaqat M. Fatima K. Fiaz S. Rasul S. Zafar H. (2021). Two-component system genes in sorghum bicolor: Genome-wide identification and expression profiling in response to environmental stresses. Front. Genet. 12, 794305. 10.3389/fgene.2021.794305 34899869 PMC8655132

[B63] Zia K. Rao M. J. Sadaqat M. Azeem F. Fatima K. ul Qamar M. T. (2022). Pangenome-wide analysis of cyclic nucleotide-gated channel (CNGC) gene family in citrus Spp. Revealed their intraspecies diversity and potential roles in abiotic stress tolerance. Front. Genet. 13, 1034921. 10.3389/fgene.2022.1034921 36303546 PMC9593079

