# Supplementary figures and images for: Genome-wide identification and expression profiling of two-component system (TCS) genes in Brassica oleracea in response to shade stress
Source: Front Genet. 2023 May 30;14:1142544. doi: 10.3389/fgene.2023.1142544 (PMC10267837; doi:10.3389/fgene.2023.1142544)

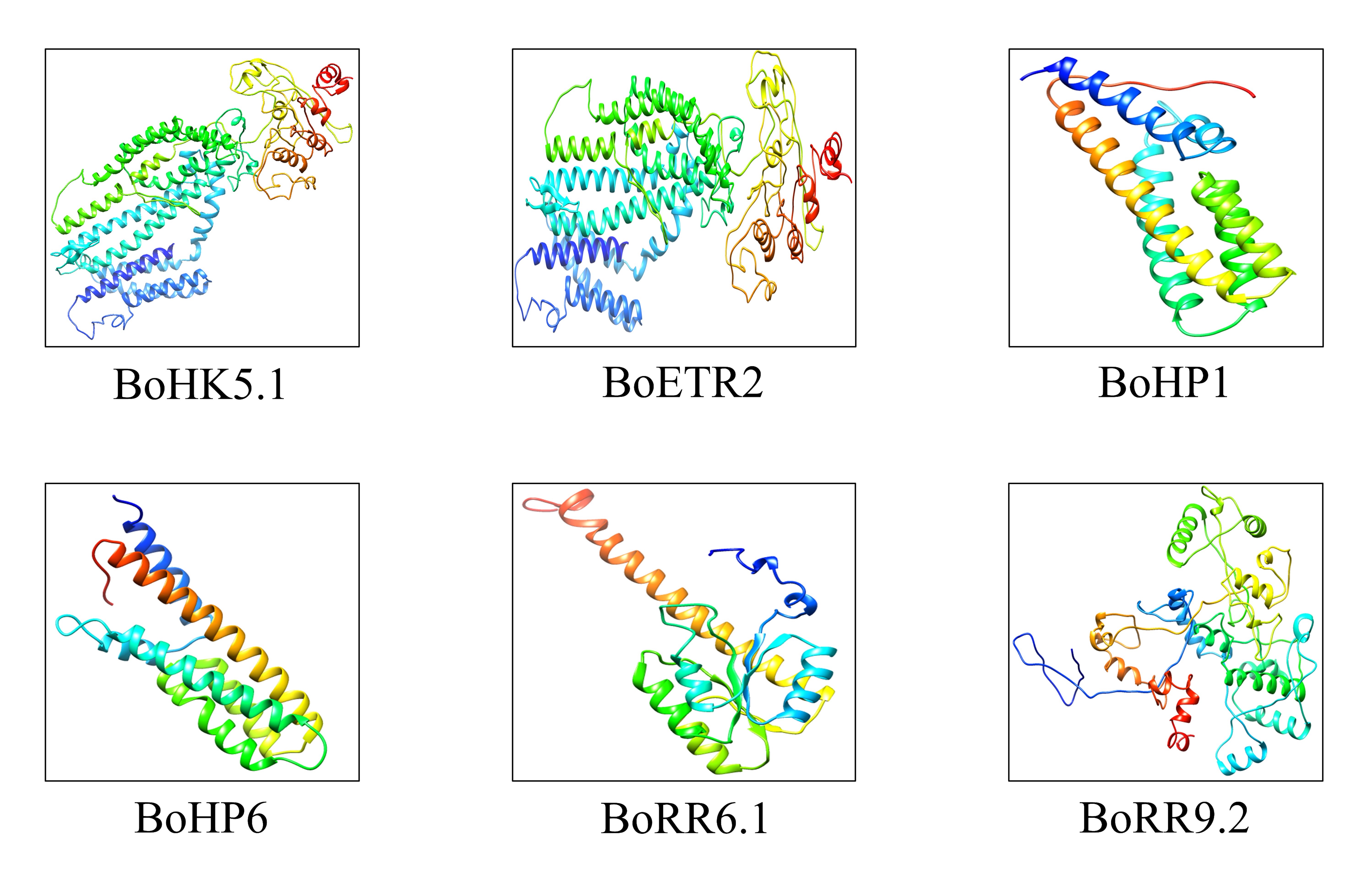

Supplement: Supplementary file 1 [file Image3.JPEG]

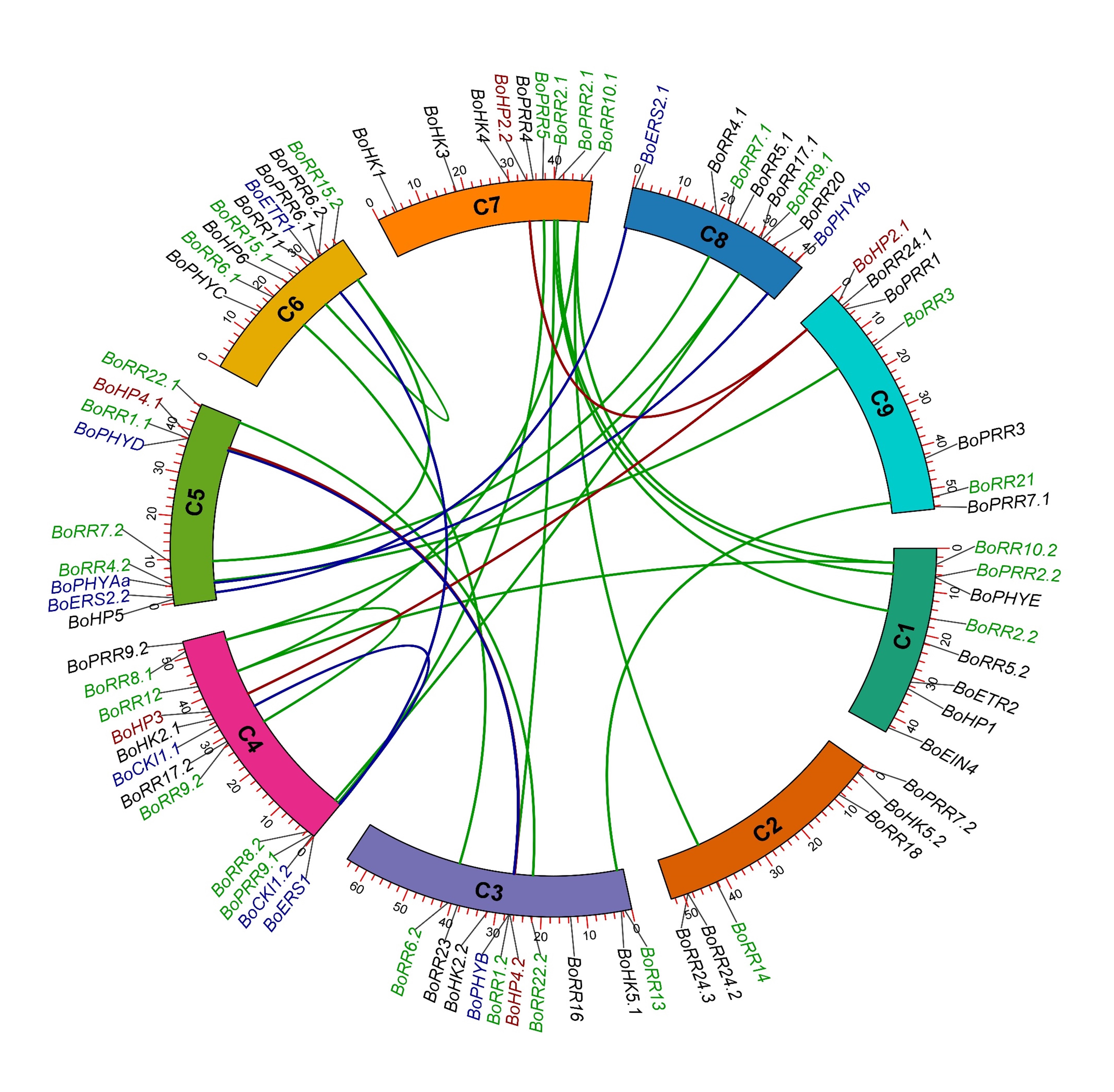

Supplement: Supplementary file 4 [file Image2.JPEG]

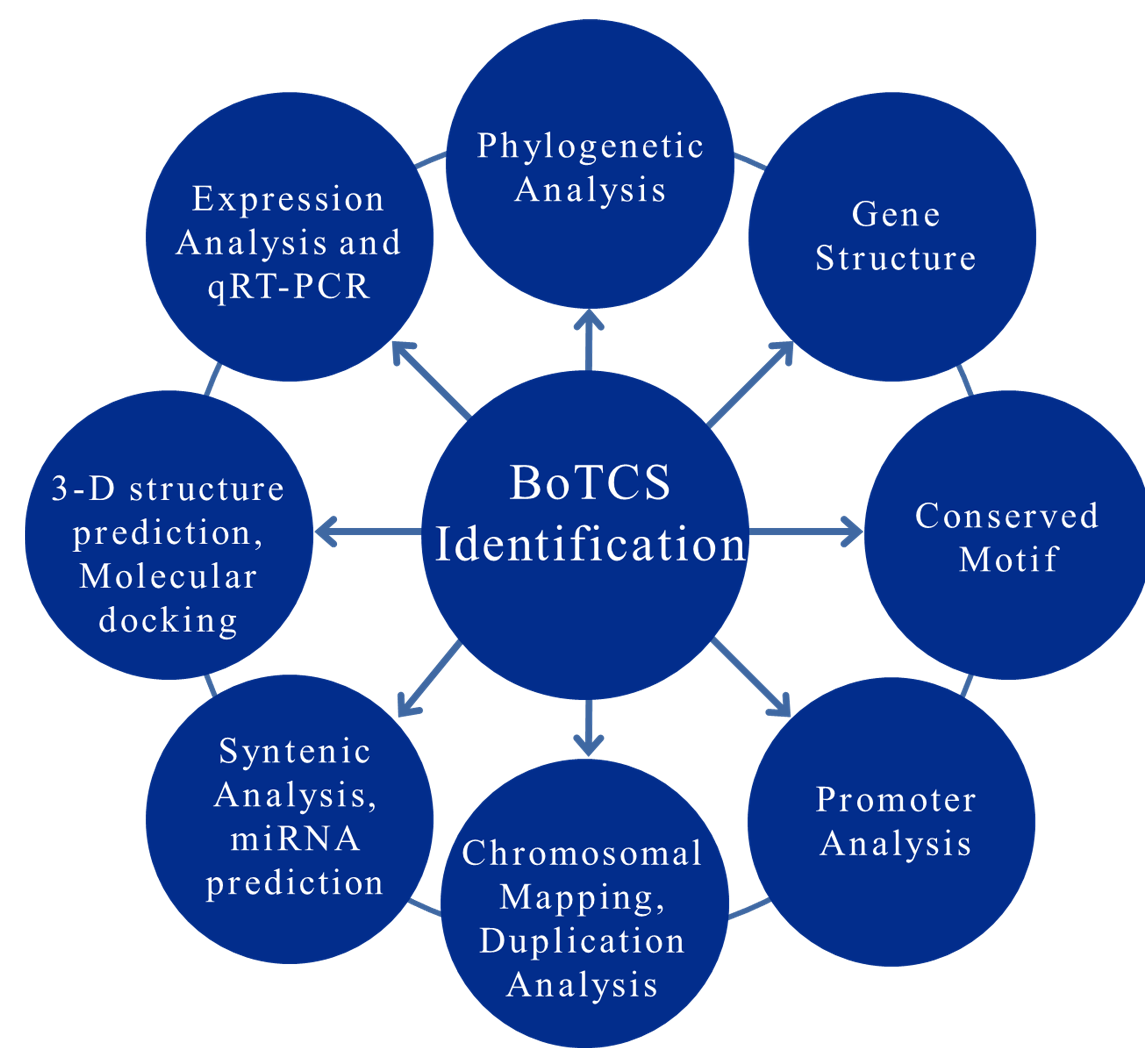

Supplement: Supplementary file 5 [file Image1.TIF]
