# Supplementary material for: Genome-wide identification and expression profiling of two-component system (TCS) genes in Brassica oleracea in response to shade stress
Source: Front Genet. 2023 May 30;14:1142544. doi: 10.3389/fgene.2023.1142544 (PMC10267837; doi:10.3389/fgene.2023.1142544)
Supplement: Supplementary file 2 [file Table1.DOCX]

**Table S1:** List of primers used for qRT-PCR.

| **Gene Name** | **Primer Sequence** |  | **Product Length** |
| --- | --- | --- | --- |
| *BoHK4* | GAAGGCGCCTATACCGTTGA | F | 99 |
|  | ATATGCACAGCTGCACCGTA | R |  |
| *BoPHYAa* | TGTCTTTGGCGTCAGGTGAA | F | 90 |
|  | CGATCCTAGCGCTGTGTCTT | R |  |
| *BoPHYAb* | TGGGAAGCATCTCCTCACAC | F | 107 |
|  | ACTCAGTGCAAGTAAAGGAAGA | R |  |
| *BoPHYB* | GATACGCTCATTTCTCGTGCG | F | 71 |
|  | TGCGATAGCAAAACGAACAACA | R |  |
| *BoPHYC* | ACAAGCGATAGGCAAACCTGT | F | 117 |
|  | ACACAAACGACATTTGCTTCCA | R |  |
| *BoPHYD* | AGAGGTACATTCAGTTCGCCA | F | 93 |
|  | CACATTCCTGTCCTCGTCCC | R |  |
| *BoPHYE* | CTCGCGCTTGCTATTGTGAC | F | 104 |
|  | TAACGTAACGGGAAGGGCAC | R |  |
| *BoRR3* | GAAACTCGCCGACGTGAAAC | F | 111 |
|  | GCCAAAGAAGAGAAACGGCTG | R |  |
| *BoRR4.1* | AAGGTCTCAACTTTCCCGCC | F | 115 |
|  | GTCGGAAACTGGACGATTCCT | R |  |
| *BoRR4.2* | CGCCTGATTCTTCGGACTCT | F | 78 |
|  | CTATCGGGGATGAGAGCTGC | R |  |
| *BoRR5.1* | TTCAGCACCTTCTTCGAGACA | F | 106 |
|  | ATGAGCTGGCCATTTTGTGG | R |  |
| *BoRR5.2* | GCTTGTTGTTATGAGCTGGCA | F | 110 |
|  | TCAGCACCTTCTTCAAGACATC | R |  |
| *BoRR6.1* | CACAGCAAATTGTTAACGGTGA | F | 105 |
|  | TCCAGTCATTCCGGGCATAG | R |  |
| *BoRR6.2* | CATGTTCTGGCCGTAGACGA | F | 105 |
|  | ACGATGAACCAAAACTTCTTCG | R |  |
| *BoRR7.1* | TGCTTTACGCCATGTTCACG | F | 89 |
|  | TAAACCAGGATGGGTTCGGGC | R |  |
| *BoRR7.2* | ACCGGTGAAACTAGCTGATGT | F | 74 |
|  | AGGTTTTGCAATCCTCTGCT | R |  |
| *BoRR8.2* | GCAACAAGTCTCCAGACCTAA | F | 111 |
|  | CTTCAGCTCCTTCTTCCAGACA | R |  |
| *BoRR9.1* | TGAGCTTATGTTGTGCGACTCT | F | 117 |
|  | TCAGGTTGTTGAGATCAGCC | R |  |
| *BoRR10.2* | GCTTCAGGGAACGCCATTTC | F | 116 |
|  | GAACCATACTGGGAGCCACC | R |  |
| *BoRR15.1* | ATGACAGAGGGAGCAGAGGA | F | 70 |
|  | GTTCTGTTAACCGCTTCACGTC | R |  |
| *BoPRR5* | TGTGGTTCTTGTTGCCAGGT |  | 91 |
|  | ACTTGCCGCAACGTTCTTTC |  |  |
| *BoPRR7.1* | GAAAGTAAGCCCGGCAGGAT |  | 119 |
|  | GTGGTGGTAACTTCCGCTCA |  |  |
| *BoPRR7.2* | AATGGGAGCATTGGCCTGAA |  | 72 |
|  | CGCATTTTCTCTTACCTGGGC |  |  |
| *BoPRR9.1* | GGGTTATGAATAATCAGCTGCG |  | 72 |
|  | CGTTCTGCTGAGAAGCTGGTA |  |  |
| *BoPRR9.2* | AGTTCAGCTGAGCCCAAGAC |  | 91 |
|  | TCTCATGGTTGCTGCTCGTT |  |  |
