# Supplementary material for: Genome-wide identification and expression profiling of two-component system (TCS) genes in Brassica oleracea in response to shade stress
Source: Front Genet. 2023 May 30;14:1142544. doi: 10.3389/fgene.2023.1142544 (PMC10267837; doi:10.3389/fgene.2023.1142544)
Supplement: Supplementary file 3 [file Table7.DOCX]

**Table S7:** Molecular docking results.

| Docaking_Complex | Binding Affinity (kcal/mol) | RMSD (Å) | Interacting Residues |
| --- | --- | --- | --- |
| BoHK5.1_ABA | -5.2 | 2.659 | HIS A:641, HIS A:643, ARG A:669, GLU A:673, LYS A: 674 |
| BoETR2_ABA | -5.6 | 2.644 | ARG A:4, ALA A:7, SER A:8, LEU A:11, ILE A:12, TYR A:61, PHE A:62 |
| BoHP1_ABA | -5.3 | 2.216 | GLN A:34, PRO A:39, VAL A:141, ILE A:147, ALA A:149 |
| BoHP6_ABA | -5 | 2.82 | PHE A:21, HIS A:22, GLU A:28, LEU A:31, ARG A:95 |
| BoRR6.1_ABA | -5.4 | 0.498 | LEU A:24, LYS A:79, ASN A:81, PHE A:160 |
| BoRR9.1_ABA | -6.1 | 0.465 | VAL A:40, LEU A: 41, LEU A: 42, LYS A:80, ARG A:452, PRO A:453, ARG A:454 |
