# Supplementary material for: Genome-wide identification and expression profiling of two-component system (TCS) genes in Brassica oleracea in response to shade stress
Source: Front Genet. 2023 May 30;14:1142544. doi: 10.3389/fgene.2023.1142544 (PMC10267837; doi:10.3389/fgene.2023.1142544)
Supplement: Supplementary file 7 [file Table2.DOCX]

**Table S1:** Summary of the identified TCS genes from different plant species.

| **Species** | **HK(L)** | **HP**  **(Pseudo-HP)** | **Type-A RR** | **Type-B RR** | **Type-C RR** | **Pseudo RR** | **Total** | **Reference** |
| --- | --- | --- | --- | --- | --- | --- | --- | --- |
| *Arabidopsis thaliana* | 17(9) | 6(1) | 10 | 12 | 2 | 9 | 47 | (Cheng and Kieber 2014) |
| *Oryza sativa L.* | 5 | 5 | 15 | 7 | 0 | 5 | 37 | (Du et al. 2007) |
| *Solanum lycopersicum* | 20(11) | 6(2) | 7 | 23 | 1 | 8 | 65 | (Y. He, Liu, Ye, et al. 2016) |
| *Zizania latifolia* | 25(4) | 8(3) | 14 | 14 | 2 | 6 | 69 | (L. He et al. 2020) |
| *Populus trichocarpa* | 12 | 12 | 9 | 11 | 0 | 5 | 49 | (Singh and Kumar 2012) |
| *Brassica rapa* | 20(9) | 8(1) | 21 | 17 | 4 | 15 | 85 | (Z. Liu et al. 2014) |
| *Cicer arietinum* | 18 | 7(2) | 7 | 7 | 2 | 10^b^ | 51 | (Ahmad et al. 2020) |
| *Glycine max* | 21 | 13 | 18 | 15 | 3 | 13 | 83 | (Le et al. 2011) |
| *Sorghum bicolor* | 13 | 5(2) | 3 | 7 | 2 | 7^b^ | 37 | (Zameer et al. 2021) |
| *Cucumis sativus L* | 18(8) | 7(2) | 8 | 8 | 0 | 5 | 46 | (Y. He, Liu, Zou, et al. 2016) |
| *Physcomitrella patens* | 18 | 3 | 7 | 5 | 2 | 4^a^ | 39 | (Satbhai et al. 2011) |
| *Cucumis melo L.* | 17(8) | 9(3) | 8 | 11 | 0 | 6 | 51 | (P. Liu et al. 2020) |
| *Triticum aestivum* | 7 | 10 | 41 | 2 | 0 | 2 | 62 | (Gahlaut et al. 2014) |
| *Zea mays* | 11(3) | 9(2) | 16 | 9 | 3 | 11^a^ | 59 | (Asakura et al. 2003) |
| *Lotus japonicus* | 14 | 7 | 7 | 11 | 1 | 5^a^ | 40 | (Ishida et al. 2009) |
| *Citrullus lanatus* | 19(9) | 6(2) | 8 | 10 | 1 | 5 | 49 | (Y. He, Liu, Zou, et al. 2016) |
| *Brassica oleracea* | 21 | 8 | 18 | 16 | 5 | 12^b^ | 80 | Present work |

a Only clock-associated.

b Both clock associated and type-B PRRs.
