# Supplementary material for: Genome-wide identification and expression profiling of two-component system (TCS) genes in Brassica oleracea in response to shade stress
Source: Front Genet. 2023 May 30;14:1142544. doi: 10.3389/fgene.2023.1142544 (PMC10267837; doi:10.3389/fgene.2023.1142544)
Supplement: Supplementary file 8 [file Table5.DOCX]

**Table S5:** *Cis*-regulatory elements present in *BoTCSs.*

| **Gene Name** | **Cis-element** | **Sequence** | **Numbers** | **Function** |
| --- | --- | --- | --- | --- |
| BoHK1 | LTR | CCGAAA | 1 | cis-acting element involved in low-temperature responsiveness |
| BoHK1 | TGA-element | AACGAC | 1 | auxin-responsive element |
| BoHK1 | MRE | AACCTAA | 1 | MYB binding site involved in light responsiveness |
| BoHK1 | GATA-motif | GATAGGG | 1 | part of a light responsive element |
| BoHK1 | GT1-motif | GGTTAA | 3 | light responsive element |
| BoHK1 | ATCT-motif | AATCTAATCC | 1 | part of a conserved DNA module involved in light responsiveness |
| BoHK1 | ARE | AAACCA | 1 | cis-acting regulatory element essential for the anaerobic induction |
| BoHK1 | Box 4 | ATTAAT | 1 | part of a conserved DNA module involved in light responsiveness |
| BoHK1 | TCA-element | CCATCTTTTT | 1 | cis-acting element involved in salicylic acid responsiveness |
| BoHK1 | AT1-motif | AATTATTTTTTATT | 1 | part of a light responsive module |
| BoHK2.1 | ARE | AAACCA | 3 | cis-acting regulatory element essential for the anaerobic induction |
| BoHK2.1 | MBS | CAACTG | 1 | MYB binding site involved in drought-inducibility |
| BoHK2.1 | I-box | cCATATCCAAT | 1 | part of a light responsive element |
| BoHK2.1 | AAAC-motif | CAATCAAAACCT | 1 | light responsive element |
| BoHK2.1 | GARE-motif | TCTGTTG | 1 | gibberellin-responsive element |
| BoHK2.1 | O2-site | GATGACATGG | 3 | cis-acting regulatory element involved in zein metabolism regulation |
| BoHK2.1 | GCN4_motif | TGAGTCA | 1 | cis-regulatory element involved in endosperm expression |
| BoHK2.1 | CGTCA-motif | CGTCA | 1 | cis-acting regulatory element involved in the MeJA-responsiveness |
| BoHK2.1 | TGACG-motif | TGACG | 1 | cis-acting regulatory element involved in the MeJA-responsiveness |
| BoHK2.1 | TC-rich repeats | GTTTTCTTAC | 1 | cis-acting element involved in defense and stress responsiveness |
| BoHK2.1 | Box II | TGGTAATAA | 1 | part of a light responsive element |
| BoHK2.1 | GTGGC-motif | CATCGTGTGGC | 1 | part of a light responsive element |
| BoHK2.1 | CAT-box | GCCACT | 1 | cis-acting regulatory element related to meristem expression |
| BoHK2.1 | LTR | CCGAAA | 2 | cis-acting element involved in low-temperature responsiveness |
| BoHK2.1 | GATA-motif | AAGGATAAGG | 1 | part of a light responsive element |
| BoHK2.2 | ABRE | ACGTG | 4 | cis-acting element involved in the abscisic acid responsiveness |
| BoHK2.2 | TCA-element | CCATCTTTTT | 1 | cis-acting element involved in salicylic acid responsiveness |
| BoHK2.2 | CGTCA-motif | CGTCA | 2 | cis-acting regulatory element involved in the MeJA-responsiveness |
| BoHK2.2 | Box 4 | ATTAAT | 1 | part of a conserved DNA module involved in light responsiveness |
| BoHK2.2 | TC-rich repeats | GTTTTCTTAC | 1 | cis-acting element involved in defense and stress responsiveness |
| BoHK2.2 | G-Box | CACGTT | 5 | cis-acting regulatory element involved in light responsiveness |
| BoHK2.2 | GARE-motif | TCTGTTG | 1 | gibberellin-responsive element |
| BoHK2.2 | TCT-motif | TCTTAC | 1 | part of a light responsive element |
| BoHK2.2 | chs-CMA1a | TTACTTAA | 1 | part of a light responsive element |
| BoHK2.2 | MRE | AACCTAA | 1 | MYB binding site involved in light responsiveness |
| BoHK2.2 | CAG-motif | GAAAGGCAGAC | 1 | part of a light response element |
| BoHK2.2 | ARE | AAACCA | 5 | cis-acting regulatory element essential for the anaerobic induction |
| BoHK2.2 | MBS | CAACTG | 2 | MYB binding site involved in drought-inducibility |
| BoHK2.2 | GT1-motif | GGTTAA | 1 | light responsive element |
| BoHK2.2 | O2-site | GATGATGTGG | 1 | cis-acting regulatory element involved in zein metabolism regulation |
| BoHK2.2 | TGACG-motif | TGACG | 2 | cis-acting regulatory element involved in the MeJA-responsiveness |
| BoHK2.2 | ATCT-motif | AATCTAATCC | 1 | part of a conserved DNA module involved in light responsiveness |
| BoHK2.2 | AE-box | AGAAACAA | 2 | part of a module for light response |
| BoHK2.2 | I-box | cCATATCCAAT | 1 | part of a light responsive element |
| BoHK2.2 | TCCC-motif | TCTCCCT | 1 | part of a light responsive element |
| BoHK2.2 | AuxRR-core | GGTCCAT | 1 | cis-acting regulatory element involved in auxin responsiveness |
| BoHK3 | MRE | AACCTAA | 1 | MYB binding site involved in light responsiveness |
| BoHK3 | MBS | CAACTG | 2 | MYB binding site involved in drought-inducibility |
| BoHK3 | O2-site | GATGATGTGG | 1 | cis-acting regulatory element involved in zein metabolism regulation |
| BoHK3 | ABRE | ACGTG | 3 | cis-acting element involved in the abscisic acid responsiveness |
| BoHK3 | TCA-element | TCAGAAGAGG | 1 | cis-acting element involved in salicylic acid responsiveness |
| BoHK3 | TCT-motif | TCTTAC | 3 | part of a light responsive element |
| BoHK3 | ARE | AAACCA | 1 | cis-acting regulatory element essential for the anaerobic induction |
| BoHK3 | G-box | TACGTG | 2 | cis-acting regulatory element involved in light responsiveness |
| BoHK3 | LTR | CCGAAA | 1 | cis-acting element involved in low-temperature responsiveness |
| BoHK3 | Box 4 | ATTAAT | 1 | part of a conserved DNA module involved in light responsiveness |
| BoHK3 | AE-box | AGAAACAA | 1 | part of a module for light response |
| BoHK3 | GT1-motif | GGTTAA | 1 | light responsive element |
| BoHK4 | G-box | CACGTC | 1 | cis-acting regulatory element involved in light responsiveness |
| BoHK4 | TGACG-motif | TGACG | 2 | cis-acting regulatory element involved in the MeJA-responsiveness |
| BoHK4 | AuxRR-core | GGTCCAT | 1 | cis-acting regulatory element involved in auxin responsiveness |
| BoHK4 | ABRE | ACGTG | 1 | cis-acting element involved in the abscisic acid responsiveness |
| BoHK4 | MBS | CAACTG | 2 | MYB binding site involved in drought-inducibility |
| BoHK4 | HD-Zip 1 | CAAT(A/T)ATTG | 1 | element involved in differentiation of the palisade mesophyll cells |
| BoHK4 | ARE | AAACCA | 4 | cis-acting regulatory element essential for the anaerobic induction |
| BoHK4 | Sp1 | GGGCGG | 1 | light responsive element |
| BoHK4 | TC-rich repeats | GTTTTCTTAC | 1 | cis-acting element involved in defense and stress responsiveness |
| BoHK4 | GARE-motif | TCTGTTG | 1 | gibberellin-responsive element |
| BoHK4 | GT1-motif | GGTTAA | 1 | light responsive element |
| BoHK4 | P-box | CCTTTTG | 1 | gibberellin-responsive element |
| BoHK4 | CGTCA-motif | CGTCA | 2 | cis-acting regulatory element involved in the MeJA-responsiveness |
| BoHK4 | I-box | GTATAAGGCC | 1 | part of a light responsive element |
| BoHK5.1 | ACE | GCGACGTACC | 1 | cis-acting element involved in light responsiveness |
| BoHK5.1 | TCT-motif | TCTTAC | 1 | part of a light responsive element |
| BoHK5.1 | TCCC-motif | TCTCCCT | 1 | part of a light responsive element |
| BoHK5.1 | ATC-motif | AGTAATCT | 1 | part of a conserved DNA module involved in light responsiveness |
| BoHK5.1 | AuxRR-core | GGTCCAT | 1 | cis-acting regulatory element involved in auxin responsiveness |
| BoHK5.1 | ARE | AAACCA | 1 | cis-acting regulatory element essential for the anaerobic induction |
| BoHK5.1 | Box 4 | ATTAAT | 2 | part of a conserved DNA module involved in light responsiveness |
| BoHK5.1 | MRE | AACCTAA | 1 | MYB binding site involved in light responsiveness |
| BoHK5.1 | G-box | CACGAC | 2 | cis-acting regulatory element involved in light responsiveness |
| BoHK5.1 | CGTCA-motif | CGTCA | 3 | cis-acting regulatory element involved in the MeJA-responsiveness |
| BoHK5.1 | MBS | CAACTG | 1 | MYB binding site involved in drought-inducibility |
| BoHK5.1 | GATA-motif | GATAGGA | 3 | part of a light responsive element |
| BoHK5.1 | CAT-box | GCCACT | 1 | cis-acting regulatory element related to meristem expression |
| BoHK5.1 | TGACG-motif | TGACG | 3 | cis-acting regulatory element involved in the MeJA-responsiveness |
| BoHK5.1 | TCA-element | CCATCTTTTT | 1 | cis-acting element involved in salicylic acid responsiveness |
| BoHK5.2 | CGTCA-motif | CGTCA | 7 | cis-acting regulatory element involved in the MeJA-responsiveness |
| BoHK5.2 | MBS | CAACTG | 1 | MYB binding site involved in drought-inducibility |
| BoHK5.2 | LTR | CCGAAA | 1 | cis-acting element involved in low-temperature responsiveness |
| BoHK5.2 | TGACG-motif | TGACG | 7 | cis-acting regulatory element involved in the MeJA-responsiveness |
| BoHK5.2 | G-Box | CACGTT | 5 | cis-acting regulatory element involved in light responsiveness |
| BoHK5.2 | GT1-motif | GGTTAAT | 1 | light responsive element |
| BoHK5.2 | 3-AF1 binding site | TAAGAGAGGAA | 1 | light responsive element |
| BoHK5.2 | ACE | CTAACGTATT | 1 | cis-acting element involved in light responsiveness |
| BoHK5.2 | ARE | AAACCA | 3 | cis-acting regulatory element essential for the anaerobic induction |
| BoHK5.2 | AuxRE | TGTCTCAATAAG | 1 | part of an auxin-responsive element |
| BoHK5.2 | ABRE | TACGGTC | 6 | cis-acting element involved in the abscisic acid responsiveness |
| BoHK5.2 | TC-rich repeats | GTTTTCTTAC | 2 | cis-acting element involved in defense and stress responsiveness |
| BoHK5.2 | GATA-motif | AAGGATAAGG | 1 | part of a light responsive element |
| BoHK5.2 | TCA-element | CCATCTTTTT | 1 | cis-acting element involved in salicylic acid responsiveness |
| BoHK5.2 | GCN4_motif | TGAGTCA | 1 | cis-regulatory element involved in endosperm expression |
| BoCKl1.1 | TCCC-motif | TCTCCCT | 3 | part of a light responsive element |
| BoCKl1.1 | ARE | AAACCA | 5 | cis-acting regulatory element essential for the anaerobic induction |
| BoCKl1.1 | CAT-box | GCCACT | 1 | cis-acting regulatory element related to meristem expression |
| BoCKl1.1 | ABRE | ACGTG | 1 | cis-acting element involved in the abscisic acid responsiveness |
| BoCKl1.1 | TCT-motif | TCTTAC | 1 | part of a light responsive element |
| BoCKl1.1 | ATCT-motif | AATCTAATCC | 1 | part of a conserved DNA module involved in light responsiveness |
| BoCKl1.1 | LTR | CCGAAA | 1 | cis-acting element involved in low-temperature responsiveness |
| BoCKl1.1 | AE-box | AGAAACTT | 1 | part of a module for light response |
| BoCKl1.1 | P-box | CCTTTTG | 1 | gibberellin-responsive element |
| BoCKl1.1 | TCA-element | CCATCTTTTT | 1 | cis-acting element involved in salicylic acid responsiveness |
| BoCKl1.1 | MBS | CAACTG | 3 | MYB binding site involved in drought-inducibility |
| BoCKl1.1 | TGACG-motif | TGACG | 1 | cis-acting regulatory element involved in the MeJA-responsiveness |
| BoCKl1.1 | G-box | CACGTC | 1 | cis-acting regulatory element involved in light responsiveness |
| BoCKl1.1 | CGTCA-motif | CGTCA | 1 | cis-acting regulatory element involved in the MeJA-responsiveness |
| BoCKl1.2 | CGTCA-motif | CGTCA | 1 | cis-acting regulatory element involved in the MeJA-responsiveness |
| BoCKl1.2 | TGACG-motif | TGACG | 1 | cis-acting regulatory element involved in the MeJA-responsiveness |
| BoCKl1.2 | GT1-motif | GGTTAA | 1 | light responsive element |
| BoCKl1.2 | G-Box | CACGTG | 2 | cis-acting regulatory element involved in light responsiveness |
| BoCKl1.2 | Box 4 | ATTAAT | 1 | part of a conserved DNA module involved in light responsiveness |
| BoCKl1.2 | CAT-box | GCCACT | 1 | cis-acting regulatory element related to meristem expression |
| BoCKl1.2 | TC-rich repeats | ATTCTCTAAC | 3 | cis-acting element involved in defense and stress responsiveness |
| BoCKl1.2 | LTR | CCGAAA | 2 | cis-acting element involved in low-temperature responsiveness |
| BoCKl1.2 | ABRE | CACGTG | 2 | cis-acting element involved in the abscisic acid responsiveness |
| BoCKl1.2 | ARE | AAACCA | 3 | cis-acting regulatory element essential for the anaerobic induction |
| BoCKl1.2 | TCT-motif | TCTTAC | 2 | part of a light responsive element |
| BoCKl1.2 | TCCC-motif | TCTCCCT | 1 | part of a light responsive element |
| BoCKl1.2 | chs-CMA2a | TCACTTGA | 1 | part of a light responsive element |
| BoCKl1.2 | circadian | CAAAGATATC | 1 | cis-acting regulatory element involved in circadian control |
| BoCKl1.2 | MRE | AACCTAA | 1 | MYB binding site involved in light responsiveness |
| BoERS1 | Box 4 | ATTAAT | 1 | part of a conserved DNA module involved in light responsiveness |
| BoERS1 | LAMP-element | CTTTATCA | 2 | part of a light responsive element |
| BoERS1 | P-box | CCTTTTG | 1 | gibberellin-responsive element |
| BoERS1 | ABRE | ACGTG | 5 | cis-acting element involved in the abscisic acid responsiveness |
| BoERS1 | CGTCA-motif | CGTCA | 3 | cis-acting regulatory element involved in the MeJA-responsiveness |
| BoERS1 | GT1-motif | GGTTAA | 2 | light responsive element |
| BoERS1 | ARE | AAACCA | 2 | cis-acting regulatory element essential for the anaerobic induction |
| BoERS1 | G-box | CACGTC | 5 | cis-acting regulatory element involved in light responsiveness |
| BoERS1 | MBS | CAACTG | 1 | MYB binding site involved in drought-inducibility |
| BoERS1 | I-box | AAGATAAGGCT | 2 | part of a light responsive element |
| BoERS1 | AuxRE | TGTCTCAATAAG | 1 | part of an auxin-responsive element |
| BoERS1 | TGACG-motif | TGACG | 3 | cis-acting regulatory element involved in the MeJA-responsiveness |
| BoERS1 | GARE-motif | TCTGTTG | 1 | gibberellin-responsive element |
| BoERS2.1 | MRE | AACCTAA | 1 | MYB binding site involved in light responsiveness |
| BoERS2.1 | GATA-motif | GATAGGA | 1 | part of a light responsive element |
| BoERS2.1 | CAT-box | GCCACT | 1 | cis-acting regulatory element related to meristem expression |
| BoERS2.1 | MBSI | aaaAaaC(G/C)GTTA | 1 | MYB binding site involved in flavonoid biosynthetic genes regulation |
| BoERS2.1 | TC-rich repeats | GTTTTCTTAC | 1 | cis-acting element involved in defense and stress responsiveness |
| BoERS2.1 | MBS | CAACTG | 2 | MYB binding site involved in drought-inducibility |
| BoERS2.1 | LTR | CCGAAA | 1 | cis-acting element involved in low-temperature responsiveness |
| BoERS2.1 | TCA-element | CCATCTTTTT | 2 | cis-acting element involved in salicylic acid responsiveness |
| BoERS2.1 | TGACG-motif | TGACG | 2 | cis-acting regulatory element involved in the MeJA-responsiveness |
| BoERS2.1 | CGTCA-motif | CGTCA | 2 | cis-acting regulatory element involved in the MeJA-responsiveness |
| BoERS2.1 | Box 4 | ATTAAT | 1 | part of a conserved DNA module involved in light responsiveness |
| BoERS2.1 | ARE | AAACCA | 1 | cis-acting regulatory element essential for the anaerobic induction |
| BoERS2.1 | TCT-motif | TCTTAC | 1 | part of a light responsive element |
| BoERS2.1 | TGA-element | AACGAC | 1 | auxin-responsive element |
| BoERS2.1 | G-box | CCACGTAA | 2 | cis-acting regulatory element involved in light responsiveness |
| BoERS2.1 | ABRE | ACGTG | 1 | cis-acting element involved in the abscisic acid responsiveness |
| BoERS2.1 | GC-motif | CCCCCG | 1 | enhancer-like element involved in anoxic specific inducibility |
| BoERS2.1 | GCN4_motif | TGAGTCA | 1 | cis-regulatory element involved in endosperm expression |
| BoERS2.1 | 3-AF1 binding site | TAAGAGAGGAA | 2 | light responsive element |
| BoERS2.2 | ARE | AAACCA | 2 | cis-acting regulatory element essential for the anaerobic induction |
| BoERS2.2 | G-Box | CACGTG | 1 | cis-acting regulatory element involved in light responsiveness |
| BoERS2.2 | GA-motif | ATAGATAA | 1 | part of a light responsive element |
| BoERS2.2 | G-box | CACGTG | 1 | cis-acting regulatory element involved in light responsiveness |
| BoERS2.2 | Box 4 | ATTAAT | 2 | part of a conserved DNA module involved in light responsiveness |
| BoERS2.2 | CGTCA-motif | CGTCA | 1 | cis-acting regulatory element involved in the MeJA-responsiveness |
| BoERS2.2 | O2-site | GATGA(C/T)(A/G)TG(A/G) | 1 | cis-acting regulatory element involved in zein metabolism regulation |
| BoERS2.2 | LTR | CCGAAA | 1 | cis-acting element involved in low-temperature responsiveness |
| BoERS2.2 | MRE | AACCTAA | 2 | MYB binding site involved in light responsiveness |
| BoERS2.2 | TCT-motif | TCTTAC | 1 | part of a light responsive element |
| BoERS2.2 | TGACG-motif | TGACG | 1 | cis-acting regulatory element involved in the MeJA-responsiveness |
| BoERS2.2 | chs-CMA2a | TCACTTGA | 1 | part of a light responsive element |
| BoERS2.2 | MBS | CAACTG | 1 | MYB binding site involved in drought-inducibility |
| BoERS2.2 | AE-box | AGAAACTT | 1 | part of a module for light response |
| BoERS2.2 | TCCC-motif | TCTCCCT | 1 | part of a light responsive element |
| BoERS2.2 | ABRE | ACGTG | 2 | cis-acting element involved in the abscisic acid responsiveness |
| BoETR1 | TC-rich repeats | ATTCTCTAAC | 2 | cis-acting element involved in defense and stress responsiveness |
| BoETR1 | G-Box | CACGTT | 1 | cis-acting regulatory element involved in light responsiveness |
| BoETR1 | MBS | CAACTG | 2 | MYB binding site involved in drought-inducibility |
| BoETR1 | Box II | ACACGTAGA | 1 | part of a light responsive element |
| BoETR1 | ABRE | ACGTG | 2 | cis-acting element involved in the abscisic acid responsiveness |
| BoETR1 | circadian | CAAAGATATC | 1 | cis-acting regulatory element involved in circadian control |
| BoETR1 | NON-box | AGATCGACG | 1 | cis-acting regulatory element related to meristem specific activation |
| BoETR1 | ARE | AAACCA | 2 | cis-acting regulatory element essential for the anaerobic induction |
| BoETR1 | I-box | TGATAATGT | 1 | part of a light responsive element |
| BoETR1 | TCT-motif | TCTTAC | 2 | part of a light responsive element |
| BoETR1 | G-box | TACGTG | 1 | cis-acting regulatory element involved in light responsiveness |
| BoETR1 | Box 4 | ATTAAT | 3 | part of a conserved DNA module involved in light responsiveness |
| BoETR1 | GATA-motif | GATAGGA | 1 | part of a light responsive element |
| BoETR1 | CGTCA-motif | CGTCA | 7 | cis-acting regulatory element involved in the MeJA-responsiveness |
| BoETR1 | TCCC-motif | TCTCCCT | 1 | part of a light responsive element |
| BoETR1 | O2-site | GATGATGTGG | 4 | cis-acting regulatory element involved in zein metabolism regulation |
| BoETR1 | TGACG-motif | TGACG | 7 | cis-acting regulatory element involved in the MeJA-responsiveness |
| BoETR2 | MBS | CAACTG | 1 | MYB binding site involved in drought-inducibility |
| BoETR2 | ARE | AAACCA | 1 | cis-acting regulatory element essential for the anaerobic induction |
| BoETR2 | G-Box | CACGTT | 1 | cis-acting regulatory element involved in light responsiveness |
| BoETR2 | O2-site | GATGACATGG | 1 | cis-acting regulatory element involved in zein metabolism regulation |
| BoETR2 | Box 4 | ATTAAT | 1 | part of a conserved DNA module involved in light responsiveness |
| BoETR2 | ATCT-motif | AATCTAATCC | 1 | part of a conserved DNA module involved in light responsiveness |
| BoETR2 | GATA-motif | GATAGGA | 1 | part of a light responsive element |
| BoETR2 | LTR | CCGAAA | 4 | cis-acting element involved in low-temperature responsiveness |
| BoETR2 | I-box | TGATAATGT | 1 | part of a light responsive element |
| BoETR2 | TCT-motif | TCTTAC | 1 | part of a light responsive element |
| BoETR2 | AE-box | AGAAACAA | 1 | part of a module for light response |
| BoETR2 | chs-CMA1a | TTACTTAA | 1 | part of a light responsive element |
| BoETR2 | TCCC-motif | TCTCCCT | 1 | part of a light responsive element |
| BoETR2 | ABRE | ACGTG | 1 | cis-acting element involved in the abscisic acid responsiveness |
| BoETR2 | TGA-element | AACGAC | 1 | auxin-responsive element |
| BoETR2 | CAT-box | GCCACT | 2 | cis-acting regulatory element related to meristem expression |
| BoETR2 | TGACG-motif | TGACG | 6 | cis-acting regulatory element involved in the MeJA-responsiveness |
| BoETR2 | TCA-element | CCATCTTTTT | 1 | cis-acting element involved in salicylic acid responsiveness |
| BoETR2 | CGTCA-motif | CGTCA | 6 | cis-acting regulatory element involved in the MeJA-responsiveness |
| BoEIN4 | Box 4 | ATTAAT | 1 | part of a conserved DNA module involved in light responsiveness |
| BoEIN4 | G-box | CACGTC | 1 | cis-acting regulatory element involved in light responsiveness |
| BoEIN4 | TGACG-motif | TGACG | 3 | cis-acting regulatory element involved in the MeJA-responsiveness |
| BoEIN4 | sbp-CMA1c | CTTTATCTCTTCCA | 1 | part of a light responsive element |
| BoEIN4 | ATCT-motif | AATCTAATCC | 1 | part of a conserved DNA module involved in light responsiveness |
| BoEIN4 | GT1-motif | GGTTAA | 4 | light responsive element |
| BoEIN4 | P-box | CCTTTTG | 1 | gibberellin-responsive element |
| BoEIN4 | ABRE | ACGTG | 1 | cis-acting element involved in the abscisic acid responsiveness |
| BoEIN4 | I-box | gGATAAGGTG | 1 | part of a light responsive element |
| BoEIN4 | TC-rich repeats | ATTCTCTAAC | 1 | cis-acting element involved in defense and stress responsiveness |
| BoEIN4 | TCCC-motif | TCTCCCT | 2 | part of a light responsive element |
| BoEIN4 | chs-CMA2a | TCACTTGA | 1 | part of a light responsive element |
| BoEIN4 | CGTCA-motif | CGTCA | 3 | cis-acting regulatory element involved in the MeJA-responsiveness |
| BoEIN4 | TGA-element | AACGAC | 1 | auxin-responsive element |
| BoEIN4 | O2-site | GATGA(C/T)(A/G)TG(A/G) | 1 | cis-acting regulatory element involved in zein metabolism regulation |
| BoEIN4 | 3-AF1 binding site | TAAGAGAGGAA | 1 | light responsive element |
| BoPHYAa | ABRE | ACGTG | 2 | cis-acting element involved in the abscisic acid responsiveness |
| BoPHYAa | O2-site | GATGATGTGG | 1 | cis-acting regulatory element involved in zein metabolism regulation |
| BoPHYAa | TATC-box | TATCCCA | 1 | cis-acting element involved in gibberellin-responsiveness |
| BoPHYAa | AT1-motif | AATTATTTTTTATT | 1 | part of a light responsive module |
| BoPHYAa | ATCT-motif | AATCTAATCC | 1 | part of a conserved DNA module involved in light responsiveness |
| BoPHYAa | TC-rich repeats | GTTTTCTTAC | 1 | cis-acting element involved in defense and stress responsiveness |
| BoPHYAa | ATC-motif | AGTAATCT | 1 | part of a conserved DNA module involved in light responsiveness |
| BoPHYAa | Box 4 | ATTAAT | 3 | part of a conserved DNA module involved in light responsiveness |
| BoPHYAa | G-box | CACGAC | 4 | cis-acting regulatory element involved in light responsiveness |
| BoPHYAa | TCT-motif | TCTTAC | 1 | part of a light responsive element |
| BoPHYAa | GC-motif | CCCCCG | 1 | enhancer-like element involved in anoxic specific inducibility |
| BoPHYAa | MBS | CAACTG | 1 | MYB binding site involved in drought-inducibility |
| BoPHYAa | TCCC-motif | TCTCCCT | 2 | part of a light responsive element |
| BoPHYAa | TCA-element | CCATCTTTTT | 2 | cis-acting element involved in salicylic acid responsiveness |
| BoPHYAa | ARE | AAACCA | 1 | cis-acting regulatory element essential for the anaerobic induction |
| BoPHYAa | CGTCA-motif | CGTCA | 1 | cis-acting regulatory element involved in the MeJA-responsiveness |
| BoPHYAa | TGACG-motif | TGACG | 1 | cis-acting regulatory element involved in the MeJA-responsiveness |
| BoPHYAb | ARE | AAACCA | 2 | cis-acting regulatory element essential for the anaerobic induction |
| BoPHYAb | MRE | AACCTAA | 1 | MYB binding site involved in light responsiveness |
| BoPHYAb | I-box | gGATAAGGTG | 1 | part of a light responsive element |
| BoPHYAb | P-box | CCTTTTG | 1 | gibberellin-responsive element |
| BoPHYAb | CGTCA-motif | CGTCA | 2 | cis-acting regulatory element involved in the MeJA-responsiveness |
| BoPHYAb | AuxRR-core | GGTCCAT | 1 | cis-acting regulatory element involved in auxin responsiveness |
| BoPHYAb | GT1-motif | GGTTAA | 3 | light responsive element |
| BoPHYAb | G-Box | CACGTT | 2 | cis-acting regulatory element involved in light responsiveness |
| BoPHYAb | TGA-element | AACGAC | 1 | auxin-responsive element |
| BoPHYAb | TGACG-motif | TGACG | 2 | cis-acting regulatory element involved in the MeJA-responsiveness |
| BoPHYAb | ABRE | ACGTG | 3 | cis-acting element involved in the abscisic acid responsiveness |
| BoPHYAb | TCT-motif | TCTTAC | 2 | part of a light responsive element |
| BoPHYAb | G-box | CACGAC | 2 | cis-acting regulatory element involved in light responsiveness |
| BoPHYAb | CAT-box | GCCACT | 1 | cis-acting regulatory element related to meristem expression |
| BoPHYAb | Gap-box | CAAATGAA(A/G)A | 1 | part of a light responsive element |
| BoPHYB | ATCT-motif | AATCTAATCC | 2 | part of a conserved DNA module involved in light responsiveness |
| BoPHYB | ABRE | ACGTG | 7 | cis-acting element involved in the abscisic acid responsiveness |
| BoPHYB | ARE | AAACCA | 1 | cis-acting regulatory element essential for the anaerobic induction |
| BoPHYB | GARE-motif | TCTGTTG | 1 | gibberellin-responsive element |
| BoPHYB | CGTCA-motif | CGTCA | 2 | cis-acting regulatory element involved in the MeJA-responsiveness |
| BoPHYB | AE-box | AGAAACTT | 1 | part of a module for light response |
| BoPHYB | TGACG-motif | TGACG | 2 | cis-acting regulatory element involved in the MeJA-responsiveness |
| BoPHYB | TCCC-motif | TCTCCCT | 1 | part of a light responsive element |
| BoPHYB | GT1-motif | GGTTAA | 1 | light responsive element |
| BoPHYB | TATC-box | TATCCCA | 1 | cis-acting element involved in gibberellin-responsiveness |
| BoPHYB | LAMP-element | CTTTATCA | 1 | part of a light responsive element |
| BoPHYB | TCA-element | CCATCTTTTT | 1 | cis-acting element involved in salicylic acid responsiveness |
| BoPHYB | TCT-motif | TCTTAC | 2 | part of a light responsive element |
| BoPHYB | TGA-element | AACGAC | 1 | auxin-responsive element |
| BoPHYB | G-box | CACGAC | 9 | cis-acting regulatory element involved in light responsiveness |
| BoPHYC | G-Box | CACGTT | 2 | cis-acting regulatory element involved in light responsiveness |
| BoPHYC | Box 4 | ATTAAT | 5 | part of a conserved DNA module involved in light responsiveness |
| BoPHYC | GATA-motif | AAGGATAAGG | 3 | part of a light responsive element |
| BoPHYC | ABRE | GACACGTGGC | 3 | cis-acting element involved in the abscisic acid responsiveness |
| BoPHYC | P-box | CCTTTTG | 1 | gibberellin-responsive element |
| BoPHYC | G-box | TACGTG | 1 | cis-acting regulatory element involved in light responsiveness |
| BoPHYC | Sp1 | GGGCGG | 1 | light responsive element |
| BoPHYC | MSA-like | (T/C)C(T/C)AACGG(T/C)(T/C)A | 1 | cis-acting element involved in cell cycle regulation |
| BoPHYC | LTR | CCGAAA | 3 | cis-acting element involved in low-temperature responsiveness |
| BoPHYC | ARE | AAACCA | 3 | cis-acting regulatory element essential for the anaerobic induction |
| BoPHYC | CAG-motif | GAAAGGCAGAC | 3 | part of a light response element |
| BoPHYD | Box 4 | ATTAAT | 2 | part of a conserved DNA module involved in light responsiveness |
| BoPHYD | MRE | AACCTAA | 1 | MYB binding site involved in light responsiveness |
| BoPHYD | ATC-motif | AGTAATCT | 2 | part of a conserved DNA module involved in light responsiveness |
| BoPHYD | ARE | AAACCA | 2 | cis-acting regulatory element essential for the anaerobic induction |
| BoPHYD | GC-motif | CCCCCG | 3 | enhancer-like element involved in anoxic specific inducibility |
| BoPHYD | TCT-motif | TCTTAC | 1 | part of a light responsive element |
| BoPHYD | chs-CMA2a | TCACTTGA | 1 | part of a light responsive element |
| BoPHYD | TCA-element | CCATCTTTTT | 1 | cis-acting element involved in salicylic acid responsiveness |
| BoPHYD | circadian | CAAAGATATC | 1 | cis-acting regulatory element involved in circadian control |
| BoPHYD | C-box | ACGAGCACCGCC | 1 | cis-acting regulatory element involved in light responsiveness |
| BoPHYD | TATC-box | TATCCCA | 1 | cis-acting element involved in gibberellin-responsiveness |
| BoPHYE | 3-AF1 binding site | TAAGAGAGGAA | 1 | light responsive element |
| BoPHYE | TGACG-motif | TGACG | 4 | cis-acting regulatory element involved in the MeJA-responsiveness |
| BoPHYE | I-box | AGATAAGG | 1 | part of a light responsive element |
| BoPHYE | MRE | AACCTAA | 1 | MYB binding site involved in light responsiveness |
| BoPHYE | Box 4 | ATTAAT | 1 | part of a conserved DNA module involved in light responsiveness |
| BoPHYE | LTR | CCGAAA | 1 | cis-acting element involved in low-temperature responsiveness |
| BoPHYE | TCA-element | CCATCTTTTT | 1 | cis-acting element involved in salicylic acid responsiveness |
| BoPHYE | AuxRR-core | GGTCCAT | 1 | cis-acting regulatory element involved in auxin responsiveness |
| BoPHYE | TCT-motif | TCTTAC | 4 | part of a light responsive element |
| BoPHYE | GCN4_motif | TGAGTCA | 1 | cis-regulatory element involved in endosperm expression |
| BoPHYE | CGTCA-motif | CGTCA | 4 | cis-acting regulatory element involved in the MeJA-responsiveness |
| BoPHYE | TATC-box | TATCCCA | 1 | cis-acting element involved in gibberellin-responsiveness |
| BoPHYE | C-box | ACGAGCACCGCC | 1 | cis-acting regulatory element involved in light responsiveness |
| BoPHYE | CAT-box | GCCACT | 2 | cis-acting regulatory element related to meristem expression |
| BoPHYE | ARE | AAACCA | 3 | cis-acting regulatory element essential for the anaerobic induction |
| BoPHYE | ABRE | ACGTG | 1 | cis-acting element involved in the abscisic acid responsiveness |
| BoPHYE | G-box | TACGTG | 2 | cis-acting regulatory element involved in light responsiveness |
| BoPHYE | circadian | CAAAGATATC | 1 | cis-acting regulatory element involved in circadian control |
| BoHP1 | GATA-motif | GATAGGG | 1 | part of a light responsive element |
| BoHP1 | ABRE | ACGTG | 3 | cis-acting element involved in the abscisic acid responsiveness |
| BoHP1 | TGACG-motif | TGACG | 2 | cis-acting regulatory element involved in the MeJA-responsiveness |
| BoHP1 | LAMP-element | CTTTATCA | 1 | part of a light responsive element |
| BoHP1 | ATCT-motif | AATCTAATCC | 1 | part of a conserved DNA module involved in light responsiveness |
| BoHP1 | ARE | AAACCA | 2 | cis-acting regulatory element essential for the anaerobic induction |
| BoHP1 | G-box | CACGTC | 2 | cis-acting regulatory element involved in light responsiveness |
| BoHP1 | RY-element | CATGCATG | 1 | cis-acting regulatory element involved in seed-specific regulation |
| BoHP1 | Gap-box | CAAATGAA(A/G)A | 1 | part of a light responsive element |
| BoHP1 | TC-rich repeats | GTTTTCTTAC | 1 | cis-acting element involved in defense and stress responsiveness |
| BoHP1 | TCT-motif | TCTTAC | 3 | part of a light responsive element |
| BoHP1 | MBS | CAACTG | 1 | MYB binding site involved in drought-inducibility |
| BoHP1 | TGA-element | AACGAC | 1 | auxin-responsive element |
| BoHP1 | AuxRR-core | GGTCCAT | 4 | cis-acting regulatory element involved in auxin responsiveness |
| BoHP1 | Box 4 | ATTAAT | 4 | part of a conserved DNA module involved in light responsiveness |
| BoHP1 | CGTCA-motif | CGTCA | 2 | cis-acting regulatory element involved in the MeJA-responsiveness |
| BoHP1 | GT1-motif | GGTTAA | 2 | light responsive element |
| BoHP1 | GARE-motif | TCTGTTG | 1 | gibberellin-responsive element |
| BoHP1 | GA-motif | ATAGATAA | 1 | part of a light responsive element |
| BoHP1 | TCA-element | CCATCTTTTT | 1 | cis-acting element involved in salicylic acid responsiveness |
| BoHP2.1 | GATA-motif | AAGATAAGATT | 1 | part of a light responsive element |
| BoHP2.1 | O2-site | GATGATGTGG | 1 | cis-acting regulatory element involved in zein metabolism regulation |
| BoHP2.1 | TCT-motif | TCTTAC | 1 | part of a light responsive element |
| BoHP2.1 | Box 4 | ATTAAT | 3 | part of a conserved DNA module involved in light responsiveness |
| BoHP2.1 | GARE-motif | TCTGTTG | 2 | gibberellin-responsive element |
| BoHP2.1 | TCCC-motif | TCTCCCT | 1 | part of a light responsive element |
| BoHP2.1 | ARE | AAACCA | 4 | cis-acting regulatory element essential for the anaerobic induction |
| BoHP2.1 | MBS | CAACTG | 2 | MYB binding site involved in drought-inducibility |
| BoHP2.1 | chs-CMA1a | TTACTTAA | 1 | part of a light responsive element |
| BoHP2.1 | TGA-element | AACGAC | 3 | auxin-responsive element |
| BoHP2.1 | G-box | CACGTC | 1 | cis-acting regulatory element involved in light responsiveness |
| BoHP2.1 | CAT-box | GCCACT | 1 | cis-acting regulatory element related to meristem expression |
| BoHP2.1 | chs-CMA2b | GAACCTACACAC | 1 | part of a light responsive element |
| BoHP2.1 | LTR | CCGAAA | 1 | cis-acting element involved in low-temperature responsiveness |
| BoHP2.1 | TC-rich repeats | GTTTTCTTAC | 1 | cis-acting element involved in defense and stress responsiveness |
| BoHP2.1 | GCN4_motif | TGAGTCA | 1 | cis-regulatory element involved in endosperm expression |
| BoHP2.1 | ABRE | CGCACGTGTC | 2 | cis-acting element involved in the abscisic acid responsiveness |
| BoHP2.1 | Sp1 | GGGCGG | 1 | light responsive element |
| BoHP2.1 | AE-box | AGAAACAA | 1 | part of a module for light response |
| BoHP2.2 | GC-motif | CCCCCG | 1 | enhancer-like element involved in anoxic specific inducibility |
| BoHP2.2 | G-Box | CACGTT | 2 | cis-acting regulatory element involved in light responsiveness |
| BoHP2.2 | AE-box | AGAAACAA | 2 | part of a module for light response |
| BoHP2.2 | TCA-element | CCATCTTTTT | 1 | cis-acting element involved in salicylic acid responsiveness |
| BoHP2.2 | TGACG-motif | TGACG | 1 | cis-acting regulatory element involved in the MeJA-responsiveness |
| BoHP2.2 | GARE-motif | TCTGTTG | 1 | gibberellin-responsive element |
| BoHP2.2 | I-box | gGATAAGGTG | 1 | part of a light responsive element |
| BoHP2.2 | GCN4_motif | TGAGTCA | 2 | cis-regulatory element involved in endosperm expression |
| BoHP2.2 | CGTCA-motif | CGTCA | 1 | cis-acting regulatory element involved in the MeJA-responsiveness |
| BoHP2.2 | LTR | CCGAAA | 3 | cis-acting element involved in low-temperature responsiveness |
| BoHP2.2 | ATC-motif | AGTAATCT | 1 | part of a conserved DNA module involved in light responsiveness |
| BoHP2.2 | ABRE | ACGTG | 3 | cis-acting element involved in the abscisic acid responsiveness |
| BoHP2.2 | ARE | AAACCA | 2 | cis-acting regulatory element essential for the anaerobic induction |
| BoHP3 | LTR | CCGAAA | 1 | cis-acting element involved in low-temperature responsiveness |
| BoHP3 | TGACG-motif | TGACG | 1 | cis-acting regulatory element involved in the MeJA-responsiveness |
| BoHP3 | ATC-motif | AGTAATCT | 1 | part of a conserved DNA module involved in light responsiveness |
| BoHP3 | HD-Zip 1 | CAAT(A/T)ATTG | 2 | element involved in differentiation of the palisade mesophyll cells |
| BoHP3 | TCA-element | CCATCTTTTT | 1 | cis-acting element involved in salicylic acid responsiveness |
| BoHP3 | CGTCA-motif | CGTCA | 1 | cis-acting regulatory element involved in the MeJA-responsiveness |
| BoHP3 | ARE | AAACCA | 5 | cis-acting regulatory element essential for the anaerobic induction |
| BoHP3 | Box 4 | ATTAAT | 3 | part of a conserved DNA module involved in light responsiveness |
| BoHP3 | MBSI | aaaAaaC(G/C)GTTA | 1 | MYB binding site involved in flavonoid biosynthetic genes regulation |
| BoHP4.1 | TCA-element | TCAGAAGAGG | 2 | cis-acting element involved in salicylic acid responsiveness |
| BoHP4.1 | AE-box | AGAAACTT | 1 | part of a module for light response |
| BoHP4.1 | Box 4 | ATTAAT | 1 | part of a conserved DNA module involved in light responsiveness |
| BoHP4.1 | G-box | CACGAC | 2 | cis-acting regulatory element involved in light responsiveness |
| BoHP4.1 | ABRE | ACGTG | 1 | cis-acting element involved in the abscisic acid responsiveness |
| BoHP4.1 | CAT-box | GCCACT | 1 | cis-acting regulatory element related to meristem expression |
| BoHP4.1 | CGTCA-motif | CGTCA | 2 | cis-acting regulatory element involved in the MeJA-responsiveness |
| BoHP4.2 | G-box | TACGTG | 1 | cis-acting regulatory element involved in light responsiveness |
| BoHP4.2 | CGTCA-motif | CGTCA | 1 | cis-acting regulatory element involved in the MeJA-responsiveness |
| BoHP4.2 | ABRE | ACGTG | 3 | cis-acting element involved in the abscisic acid responsiveness |
| BoHP4.2 | TCA-element | CCATCTTTTT | 1 | cis-acting element involved in salicylic acid responsiveness |
| BoHP4.2 | MBS | CAACTG | 2 | MYB binding site involved in drought-inducibility |
| BoHP4.2 | G-Box | CACGTT | 1 | cis-acting regulatory element involved in light responsiveness |
| BoHP4.2 | CAT-box | GCCACT | 2 | cis-acting regulatory element related to meristem expression |
| BoHP4.2 | MRE | AACCTAA | 1 | MYB binding site involved in light responsiveness |
| BoHP4.2 | ATCT-motif | AATCTAATCC | 1 | part of a conserved DNA module involved in light responsiveness |
| BoHP4.2 | LTR | CCGAAA | 1 | cis-acting element involved in low-temperature responsiveness |
| BoHP4.2 | AE-box | AGAAACAA | 1 | part of a module for light response |
| BoHP4.2 | circadian | CAAAGATATC | 1 | cis-acting regulatory element involved in circadian control |
| BoHP4.2 | GT1-motif | GTGTGTGAA | 1 | light responsive element |
| BoHP4.2 | TC-rich repeats | ATTCTCTAAC | 1 | cis-acting element involved in defense and stress responsiveness |
| BoHP4.2 | Box 4 | ATTAAT | 8 | part of a conserved DNA module involved in light responsiveness |
| BoHP4.2 | TGACG-motif | TGACG | 1 | cis-acting regulatory element involved in the MeJA-responsiveness |
| BoHP4.2 | GA-motif | ATAGATAA | 1 | part of a light responsive element |
| BoHP4.2 | ARE | AAACCA | 1 | cis-acting regulatory element essential for the anaerobic induction |
| BoHP5 | TGACG-motif | TGACG | 2 | cis-acting regulatory element involved in the MeJA-responsiveness |
| BoHP5 | AE-box | AGAAACTT | 1 | part of a module for light response |
| BoHP5 | G-box | TACGTG | 7 | cis-acting regulatory element involved in light responsiveness |
| BoHP5 | GA-motif | ATAGATAA | 1 | part of a light responsive element |
| BoHP5 | TCT-motif | TCTTAC | 1 | part of a light responsive element |
| BoHP5 | circadian | CAAAGATATC | 1 | cis-acting regulatory element involved in circadian control |
| BoHP5 | AuxRR-core | GGTCCAT | 1 | cis-acting regulatory element involved in auxin responsiveness |
| BoHP5 | CGTCA-motif | CGTCA | 2 | cis-acting regulatory element involved in the MeJA-responsiveness |
| BoHP5 | Box 4 | ATTAAT | 5 | part of a conserved DNA module involved in light responsiveness |
| BoHP5 | ARE | AAACCA | 3 | cis-acting regulatory element essential for the anaerobic induction |
| BoHP5 | GT1-motif | GGTTAA | 1 | light responsive element |
| BoHP5 | ABRE | ACGTG | 5 | cis-acting element involved in the abscisic acid responsiveness |
| BoHP6 | LTR | CCGAAA | 3 | cis-acting element involved in low-temperature responsiveness |
| BoHP6 | Box 4 | ATTAAT | 1 | part of a conserved DNA module involved in light responsiveness |
| BoHP6 | TCA-element | CCATCTTTTT | 1 | cis-acting element involved in salicylic acid responsiveness |
| BoHP6 | GA-motif | ATAGATAA | 1 | part of a light responsive element |
| BoHP6 | ARE | AAACCA | 2 | cis-acting regulatory element essential for the anaerobic induction |
| BoHP6 | AT1-motif | AATTATTTTTTATT | 1 | part of a light responsive module |
| BoHP6 | TATC-box | TATCCCA | 1 | cis-acting element involved in gibberellin-responsiveness |
| BoHP6 | GATA-motif | GATAGGA | 2 | part of a light responsive element |
| BoHP6 | ABRE | ACGTG | 2 | cis-acting element involved in the abscisic acid responsiveness |
| BoHP6 | CGTCA-motif | CGTCA | 1 | cis-acting regulatory element involved in the MeJA-responsiveness |
| BoHP6 | ACE | GACACGTATG | 1 | cis-acting element involved in light responsiveness |
| BoHP6 | TGACG-motif | TGACG | 1 | cis-acting regulatory element involved in the MeJA-responsiveness |
| BoHP6 | G-box | CACGTC | 2 | cis-acting regulatory element involved in light responsiveness |
| BoHP6 | MBS | CAACTG | 1 | MYB binding site involved in drought-inducibility |
| BoHP6 | TCT-motif | TCTTAC | 1 | part of a light responsive element |
| BoHP6 | SARE | TTCGACCATCTT | 1 | cis-acting element involved in salicylic acid responsiveness |
| BoRR1.1 | CAT-box | GCCACT | 1 | cis-acting regulatory element related to meristem expression |
| BoRR1.1 | Sp1 | GGGCGG | 1 | light responsive element |
| BoRR1.1 | ARE | AAACCA | 4 | cis-acting regulatory element essential for the anaerobic induction |
| BoRR1.1 | GA-motif | ATAGATAA | 1 | part of a light responsive element |
| BoRR1.1 | CGTCA-motif | CGTCA | 1 | cis-acting regulatory element involved in the MeJA-responsiveness |
| BoRR1.1 | TGA-element | AACGAC | 3 | auxin-responsive element |
| BoRR1.1 | ABRE | ACGTG | 2 | cis-acting element involved in the abscisic acid responsiveness |
| BoRR1.1 | TGACG-motif | TGACG | 1 | cis-acting regulatory element involved in the MeJA-responsiveness |
| BoRR1.1 | G-Box | CACGTT | 1 | cis-acting regulatory element involved in light responsiveness |
| BoRR1.1 | MRE | AACCTAA | 2 | MYB binding site involved in light responsiveness |
| BoRR1.1 | GT1-motif | GGTTAA | 1 | light responsive element |
| BoRR1.1 | P-box | CCTTTTG | 1 | gibberellin-responsive element |
| BoRR1.1 | TCA-element | TCAGAAGAGG | 1 | cis-acting element involved in salicylic acid responsiveness |
| BoRR1.1 | TCCC-motif | TCTCCCT | 1 | part of a light responsive element |
| BoRR1.1 | LTR | CCGAAA | 1 | cis-acting element involved in low-temperature responsiveness |
| BoRR1.1 | circadian | CAAAGATATC | 2 | cis-acting regulatory element involved in circadian control |
| BoRR1.1 | GC-motif | CCCCCG | 2 | enhancer-like element involved in anoxic specific inducibility |
| BoRR1.1 | MBS | CAACTG | 1 | MYB binding site involved in drought-inducibility |
| BoRR1.1 | ACE | CTAACGTATT | 1 | cis-acting element involved in light responsiveness |
| BoRR1.1 | Box 4 | ATTAAT | 1 | part of a conserved DNA module involved in light responsiveness |
| BoRR1.1 | TCT-motif | TCTTAC | 1 | part of a light responsive element |
| BoRR1.2 | MRE | AACCTAA | 1 | MYB binding site involved in light responsiveness |
| BoRR1.2 | ARE | AAACCA | 2 | cis-acting regulatory element essential for the anaerobic induction |
| BoRR1.2 | Box 4 | ATTAAT | 2 | part of a conserved DNA module involved in light responsiveness |
| BoRR1.2 | G-Box | CACGTT | 1 | cis-acting regulatory element involved in light responsiveness |
| BoRR1.2 | I-box | AGATAAGG | 3 | part of a light responsive element |
| BoRR1.2 | ABRE | AACCCGG | 2 | cis-acting element involved in the abscisic acid responsiveness |
| BoRR1.2 | TC-rich repeats | GTTTTCTTAC | 1 | cis-acting element involved in defense and stress responsiveness |
| BoRR1.2 | CAT-box | GCCACT | 1 | cis-acting regulatory element related to meristem expression |
| BoRR1.2 | MBS | CAACTG | 1 | MYB binding site involved in drought-inducibility |
| BoRR1.2 | GATA-motif | AAGGATAAGG | 1 | part of a light responsive element |
| BoRR1.2 | LTR | CCGAAA | 4 | cis-acting element involved in low-temperature responsiveness |
| BoRR2.1 | MBS | CAACTG | 1 | MYB binding site involved in drought-inducibility |
| BoRR2.1 | TCCC-motif | TCTCCCT | 1 | part of a light responsive element |
| BoRR2.1 | O2-site | GATGACATGG | 1 | cis-acting regulatory element involved in zein metabolism regulation |
| BoRR2.1 | ARE | AAACCA | 1 | cis-acting regulatory element essential for the anaerobic induction |
| BoRR2.1 | TCA-element | CCATCTTTTT | 1 | cis-acting element involved in salicylic acid responsiveness |
| BoRR2.1 | Box 4 | ATTAAT | 7 | part of a conserved DNA module involved in light responsiveness |
| BoRR2.1 | LTR | CCGAAA | 1 | cis-acting element involved in low-temperature responsiveness |
| BoRR2.1 | GT1-motif | GGTTAAT | 1 | light responsive element |
| BoRR2.1 | TC-rich repeats | GTTTTCTTAC | 1 | cis-acting element involved in defense and stress responsiveness |
| BoRR2.1 | TCT-motif | TCTTAC | 3 | part of a light responsive element |
| BoRR2.1 | AT1-motif | AATTATTTTTTATT | 1 | part of a light responsive module |
| BoRR2.2 | MBS | CAACTG | 2 | MYB binding site involved in drought-inducibility |
| BoRR2.2 | G-Box | CACGTT | 1 | cis-acting regulatory element involved in light responsiveness |
| BoRR2.2 | Box 4 | ATTAAT | 1 | part of a conserved DNA module involved in light responsiveness |
| BoRR2.2 | TGA-element | AACGAC | 1 | auxin-responsive element |
| BoRR2.2 | G-box | TAACACGTAG | 1 | cis-acting regulatory element involved in light responsiveness |
| BoRR2.2 | chs-CMA1a | TTACTTAA | 1 | part of a light responsive element |
| BoRR2.2 | GARE-motif | TCTGTTG | 2 | gibberellin-responsive element |
| BoRR2.2 | ARE | AAACCA | 2 | cis-acting regulatory element essential for the anaerobic induction |
| BoRR2.2 | TCT-motif | TCTTAC | 2 | part of a light responsive element |
| BoRR2.2 | ABRE | AACCCGG | 2 | cis-acting element involved in the abscisic acid responsiveness |
| BoRR2.2 | AE-box | AGAAACAA | 2 | part of a module for light response |
| BoRR2.2 | GCN4_motif | TGAGTCA | 1 | cis-regulatory element involved in endosperm expression |
| BoRR3 | CGTCA-motif | CGTCA | 3 | cis-acting regulatory element involved in the MeJA-responsiveness |
| BoRR3 | TC-rich repeats | ATTCTCTAAC | 3 | cis-acting element involved in defense and stress responsiveness |
| BoRR3 | LTR | CCGAAA | 1 | cis-acting element involved in low-temperature responsiveness |
| BoRR3 | G-box | CACGAC | 1 | cis-acting regulatory element involved in light responsiveness |
| BoRR3 | GARE-motif | TCTGTTG | 2 | gibberellin-responsive element |
| BoRR3 | MBS | CAACTG | 2 | MYB binding site involved in drought-inducibility |
| BoRR3 | TGACG-motif | TGACG | 3 | cis-acting regulatory element involved in the MeJA-responsiveness |
| BoRR3 | O2-site | GATGATGTGG | 1 | cis-acting regulatory element involved in zein metabolism regulation |
| BoRR3 | TCT-motif | TCTTAC | 3 | part of a light responsive element |
| BoRR3 | TCA-element | CCATCTTTTT | 1 | cis-acting element involved in salicylic acid responsiveness |
| BoRR3 | Box 4 | ATTAAT | 1 | part of a conserved DNA module involved in light responsiveness |
| BoRR3 | ABRE | CGTACGTGCA | 1 | cis-acting element involved in the abscisic acid responsiveness |
| BoRR3 | chs-CMA1a | TTACTTAA | 2 | part of a light responsive element |
| BoRR3 | RY-element | CATGCATG | 1 | cis-acting regulatory element involved in seed-specific regulation |
| BoRR3 | ARE | AAACCA | 3 | cis-acting regulatory element essential for the anaerobic induction |
| BoRR3 | P-box | CCTTTTG | 1 | gibberellin-responsive element |
| BoRR3 | GT1-motif | GGTTAAT | 2 | light responsive element |
| BoRR4.1 | Box 4 | ATTAAT | 5 | part of a conserved DNA module involved in light responsiveness |
| BoRR4.1 | Box 4 | ATTAAT | 1 | part of a conserved DNA module involved in light responsiveness |
| BoRR4.1 | O2-site | GATGACATGG | 1 | cis-acting regulatory element involved in zein metabolism regulation |
| BoRR4.1 | TC-rich repeats | GTTTTCTTAC | 1 | cis-acting element involved in defense and stress responsiveness |
| BoRR4.1 | ARE | AAACCA | 1 | cis-acting regulatory element essential for the anaerobic induction |
| BoRR4.1 | GARE-motif | TCTGTTG | 1 | gibberellin-responsive element |
| BoRR4.1 | G-Box | CACGTT | 2 | cis-acting regulatory element involved in light responsiveness |
| BoRR4.1 | ABRE | TACGGTC | 3 | cis-acting element involved in the abscisic acid responsiveness |
| BoRR4.1 | GATA-motif | AAGATAAGATT | 1 | part of a light responsive element |
| BoRR4.1 | GA-motif | ATAGATAA | 1 | part of a light responsive element |
| BoRR4.1 | TCT-motif | TCTTAC | 2 | part of a light responsive element |
| BoRR4.2 | circadian | CAAAGATATC | 1 | cis-acting regulatory element involved in circadian control |
| BoRR4.2 | CGTCA-motif | CGTCA | 1 | cis-acting regulatory element involved in the MeJA-responsiveness |
| BoRR4.2 | TCT-motif | TCTTAC | 1 | part of a light responsive element |
| BoRR4.2 | L-box | ATCCCACCTAC | 1 | part of a light responsive element |
| BoRR4.2 | CAT-box | GCCACT | 1 | cis-acting regulatory element related to meristem expression |
| BoRR4.2 | ATCT-motif | AATCTAATCC | 1 | part of a conserved DNA module involved in light responsiveness |
| BoRR4.2 | G-Box | CACGTT | 2 | cis-acting regulatory element involved in light responsiveness |
| BoRR4.2 | TGACG-motif | TGACG | 1 | cis-acting regulatory element involved in the MeJA-responsiveness |
| BoRR4.2 | G-box | TAAACGTG | 2 | cis-acting regulatory element involved in light responsiveness |
| BoRR4.2 | TCA-element | CCATCTTTTT | 1 | cis-acting element involved in salicylic acid responsiveness |
| BoRR4.2 | GATA-motif | AAGGATAAGG | 1 | part of a light responsive element |
| BoRR4.2 | MBS | CAACTG | 3 | MYB binding site involved in drought-inducibility |
| BoRR4.2 | TC-rich repeats | GTTTTCTTAC | 1 | cis-acting element involved in defense and stress responsiveness |
| BoRR4.2 | ABRE | ACGTG | 3 | cis-acting element involved in the abscisic acid responsiveness |
| BoRR4.2 | ARE | AAACCA | 3 | cis-acting regulatory element essential for the anaerobic induction |
| BoRR4.2 | Box 4 | ATTAAT | 1 | part of a conserved DNA module involved in light responsiveness |
| BoRR4.2 | P-box | CCTTTTG | 1 | gibberellin-responsive element |
| BoRR4.2 | LAMP-element | CTTTATCA | 1 | part of a light responsive element |
| BoRR4.2 | O2-site | GATGA(C/T)(A/G)TG(A/G) | 1 | cis-acting regulatory element involved in zein metabolism regulation |
| BoRR5.1 | G-box | CCACGTAA | 11 | cis-acting regulatory element involved in light responsiveness |
| BoRR5.1 | GARE-motif | TCTGTTG | 1 | gibberellin-responsive element |
| BoRR5.1 | AE-box | AGAAACTT | 1 | part of a module for light response |
| BoRR5.1 | O2-site | GATGATGTGG | 1 | cis-acting regulatory element involved in zein metabolism regulation |
| BoRR5.1 | LTR | CCGAAA | 2 | cis-acting element involved in low-temperature responsiveness |
| BoRR5.1 | CAT-box | GCCACT | 1 | cis-acting regulatory element related to meristem expression |
| BoRR5.1 | P-box | TTCCAACAAACCCC | 1 | gibberellin-responsive element and part of a light responsive element |
| BoRR5.1 | GC-motif | CCCCCG | 2 | enhancer-like element involved in anoxic specific inducibility |
| BoRR5.1 | GATA-motif | GATAGGG | 1 | part of a light responsive element |
| BoRR5.1 | ARE | AAACCA | 4 | cis-acting regulatory element essential for the anaerobic induction |
| BoRR5.1 | CGTCA-motif | CGTCA | 4 | cis-acting regulatory element involved in the MeJA-responsiveness |
| BoRR5.1 | MRE | AACCTAA | 3 | MYB binding site involved in light responsiveness |
| BoRR5.1 | TGA-element | AACGAC | 1 | auxin-responsive element |
| BoRR5.1 | Box 4 | ATTAAT | 3 | part of a conserved DNA module involved in light responsiveness |
| BoRR5.1 | TGACG-motif | TGACG | 4 | cis-acting regulatory element involved in the MeJA-responsiveness |
| BoRR5.1 | ABRE | ACGTG | 9 | cis-acting element involved in the abscisic acid responsiveness |
| BoRR5.2 | ABRE | TACGGTC | 4 | cis-acting element involved in the abscisic acid responsiveness |
| BoRR5.2 | TGACG-motif | TGACG | 1 | cis-acting regulatory element involved in the MeJA-responsiveness |
| BoRR5.2 | GT1-motif | GGTTAA | 3 | light responsive element |
| BoRR5.2 | AT1-motif | AATTATTTTTTATT | 1 | part of a light responsive module |
| BoRR5.2 | ARE | AAACCA | 2 | cis-acting regulatory element essential for the anaerobic induction |
| BoRR5.2 | ARE | AAACCA | 3 | cis-acting regulatory element essential for the anaerobic induction |
| BoRR5.2 | P-box | CCTTTTG | 1 | gibberellin-responsive element |
| BoRR5.2 | CGTCA-motif | CGTCA | 1 | cis-acting regulatory element involved in the MeJA-responsiveness |
| BoRR5.2 | G-box | TACGTG | 2 | cis-acting regulatory element involved in light responsiveness |
| BoRR5.2 | TC-rich repeats | ATTCTCTAAC | 3 | cis-acting element involved in defense and stress responsiveness |
| BoRR5.2 | Box 4 | ATTAAT | 6 | part of a conserved DNA module involved in light responsiveness |
| BoRR6.1 | CGTCA-motif | CGTCA | 2 | cis-acting regulatory element involved in the MeJA-responsiveness |
| BoRR6.1 | LTR | CCGAAA | 1 | cis-acting element involved in low-temperature responsiveness |
| BoRR6.1 | L-box | ATCCCACCTAC | 1 | part of a light responsive element |
| BoRR6.1 | TCA-element | CCATCTTTTT | 1 | cis-acting element involved in salicylic acid responsiveness |
| BoRR6.1 | GATA-motif | AAGGATAAGG | 1 | part of a light responsive element |
| BoRR6.1 | ABRE | CGCACGTGTC | 3 | cis-acting element involved in the abscisic acid responsiveness |
| BoRR6.1 | ARE | AAACCA | 5 | cis-acting regulatory element essential for the anaerobic induction |
| BoRR6.1 | G-Box | CACGTG | 2 | cis-acting regulatory element involved in light responsiveness |
| BoRR6.1 | TCT-motif | TCTTAC | 2 | part of a light responsive element |
| BoRR6.1 | Box 4 | ATTAAT | 2 | part of a conserved DNA module involved in light responsiveness |
| BoRR6.1 | AE-box | AGAAACAA | 1 | part of a module for light response |
| BoRR6.1 | TGACG-motif | TGACG | 2 | cis-acting regulatory element involved in the MeJA-responsiveness |
| BoRR6.1 | TC-rich repeats | GTTTTCTTAC | 2 | cis-acting element involved in defense and stress responsiveness |
| BoRR6.1 | P-box | CCTTTTG | 1 | gibberellin-responsive element |
| BoRR6.1 | GT1-motif | GTGTGTGAA | 3 | light responsive element |
| BoRR6.1 | GARE-motif | TCTGTTG | 1 | gibberellin-responsive element |
| BoRR6.2 | ABRE | ACGTG | 2 | cis-acting element involved in the abscisic acid responsiveness |
| BoRR6.2 | TGACG-motif | TGACG | 5 | cis-acting regulatory element involved in the MeJA-responsiveness |
| BoRR6.2 | TCT-motif | TCTTAC | 1 | part of a light responsive element |
| BoRR6.2 | I-box | gGATAAGGTG | 2 | part of a light responsive element |
| BoRR6.2 | Box 4 | ATTAAT | 4 | part of a conserved DNA module involved in light responsiveness |
| BoRR6.2 | G-box | CACGTC | 3 | cis-acting regulatory element involved in light responsiveness |
| BoRR6.2 | ARE | AAACCA | 2 | cis-acting regulatory element essential for the anaerobic induction |
| BoRR6.2 | CGTCA-motif | CGTCA | 5 | cis-acting regulatory element involved in the MeJA-responsiveness |
| BoRR6.2 | MBS | CAACTG | 2 | MYB binding site involved in drought-inducibility |
| BoRR7.1 | chs-CMA1a | TTACTTAA | 1 | part of a light responsive element |
| BoRR7.1 | TGA-element | AACGAC | 1 | auxin-responsive element |
| BoRR7.1 | O2-site | GATGATGTGG | 1 | cis-acting regulatory element involved in zein metabolism regulation |
| BoRR7.1 | GT1-motif | GGTTAA | 4 | light responsive element |
| BoRR7.1 | MBS | CAACTG | 3 | MYB binding site involved in drought-inducibility |
| BoRR7.1 | TCT-motif | TCTTAC | 2 | part of a light responsive element |
| BoRR7.1 | ARE | AAACCA | 1 | cis-acting regulatory element essential for the anaerobic induction |
| BoRR7.1 | CGTCA-motif | CGTCA | 1 | cis-acting regulatory element involved in the MeJA-responsiveness |
| BoRR7.1 | G-box | CACGTG | 2 | cis-acting regulatory element involved in light responsiveness |
| BoRR7.1 | MRE | AACCTAA | 2 | MYB binding site involved in light responsiveness |
| BoRR7.1 | Box 4 | ATTAAT | 1 | part of a conserved DNA module involved in light responsiveness |
| BoRR7.1 | LTR | CCGAAA | 1 | cis-acting element involved in low-temperature responsiveness |
| BoRR7.1 | ABRE | TACGGTC | 6 | cis-acting element involved in the abscisic acid responsiveness |
| BoRR7.1 | TCCC-motif | TCTCCCT | 1 | part of a light responsive element |
| BoRR7.1 | TGACG-motif | TGACG | 1 | cis-acting regulatory element involved in the MeJA-responsiveness |
| BoRR7.1 | G-Box | CACGTG | 2 | cis-acting regulatory element involved in light responsiveness |
| BoRR7.2 | LTR | CCGAAA | 1 | cis-acting element involved in low-temperature responsiveness |
| BoRR7.2 | Box 4 | ATTAAT | 2 | part of a conserved DNA module involved in light responsiveness |
| BoRR7.2 | G-box | CACGAC | 1 | cis-acting regulatory element involved in light responsiveness |
| BoRR7.2 | ABRE | CGTACGTGCA | 1 | cis-acting element involved in the abscisic acid responsiveness |
| BoRR7.2 | AE-box | AGAAACTT | 3 | part of a module for light response |
| BoRR7.2 | P-box | CCTTTTG | 1 | gibberellin-responsive element |
| BoRR7.2 | MBS | CAACTG | 1 | MYB binding site involved in drought-inducibility |
| BoRR7.2 | GATA-motif | AAGATAAGATT | 1 | part of a light responsive element |
| BoRR7.2 | ATCT-motif | AATCTAATCC | 1 | part of a conserved DNA module involved in light responsiveness |
| BoRR7.2 | GTGGC-motif | CAGCGTGTGGC | 1 | part of a light responsive element |
| BoRR7.2 | MBSI | TTTTTACGGTTA | 1 | MYB binding site involved in flavonoid biosynthetic genes regulation |
| BoRR8.1 | TGA-element | AACGAC | 1 | auxin-responsive element |
| BoRR8.1 | G-box | TACGTG | 1 | cis-acting regulatory element involved in light responsiveness |
| BoRR8.1 | ARE | AAACCA | 1 | cis-acting regulatory element essential for the anaerobic induction |
| BoRR8.1 | TCT-motif | TCTTAC | 2 | part of a light responsive element |
| BoRR8.1 | TGACG-motif | TGACG | 1 | cis-acting regulatory element involved in the MeJA-responsiveness |
| BoRR8.1 | ACE | GACACGTATG | 1 | cis-acting element involved in light responsiveness |
| BoRR8.1 | AuxRR-core | GGTCCAT | 1 | cis-acting regulatory element involved in auxin responsiveness |
| BoRR8.1 | GA-motif | ATAGATAA | 1 | part of a light responsive element |
| BoRR8.1 | TATC-box | TATCCCA | 3 | cis-acting element involved in gibberellin-responsiveness |
| BoRR8.1 | P-box | CCTTTTG | 1 | gibberellin-responsive element |
| BoRR8.1 | MBS | CAACTG | 1 | MYB binding site involved in drought-inducibility |
| BoRR8.1 | I-box | AGATAAGG | 1 | part of a light responsive element |
| BoRR8.1 | ABRE | ACGTG | 3 | cis-acting element involved in the abscisic acid responsiveness |
| BoRR8.1 | CAT-box | GCCACT | 1 | cis-acting regulatory element related to meristem expression |
| BoRR8.1 | circadian | CAAAGATATC | 1 | cis-acting regulatory element involved in circadian control |
| BoRR8.1 | G-Box | CACGTT | 1 | cis-acting regulatory element involved in light responsiveness |
| BoRR8.1 | chs-CMA2a | TCACTTGA | 1 | part of a light responsive element |
| BoRR8.1 | GT1-motif | GGTTAAT | 4 | light responsive element |
| BoRR8.1 | CGTCA-motif | CGTCA | 1 | cis-acting regulatory element involved in the MeJA-responsiveness |
| BoRR8.2 | ARE | AAACCA | 3 | cis-acting regulatory element essential for the anaerobic induction |
| BoRR8.2 | Sp1 | GGGCGG | 1 | light responsive element |
| BoRR8.2 | G-box | CACGAC | 4 | cis-acting regulatory element involved in light responsiveness |
| BoRR8.2 | AE-box | AGAAACTT | 1 | part of a module for light response |
| BoRR8.2 | ACE | GACACGTATG | 1 | cis-acting element involved in light responsiveness |
| BoRR8.2 | TGACG-motif | TGACG | 1 | cis-acting regulatory element involved in the MeJA-responsiveness |
| BoRR8.2 | O2-site | GATGATGTGG | 2 | cis-acting regulatory element involved in zein metabolism regulation |
| BoRR8.2 | LTR | CCGAAA | 1 | cis-acting element involved in low-temperature responsiveness |
| BoRR8.2 | TCA-element | CCATCTTTTT | 2 | cis-acting element involved in salicylic acid responsiveness |
| BoRR8.2 | ATCT-motif | AATCTAATCC | 1 | part of a conserved DNA module involved in light responsiveness |
| BoRR8.2 | GT1-motif | GGTTAAT | 2 | light responsive element |
| BoRR8.2 | chs-CMA2a | TCACTTGA | 1 | part of a light responsive element |
| BoRR8.2 | ABRE | ACGTG | 3 | cis-acting element involved in the abscisic acid responsiveness |
| BoRR8.2 | CGTCA-motif | CGTCA | 1 | cis-acting regulatory element involved in the MeJA-responsiveness |
| BoRR8.2 | I-box | cCATATCCAAT | 1 | part of a light responsive element |
| BoRR8.2 | TCT-motif | TCTTAC | 3 | part of a light responsive element |
| BoRR8.2 | MRE | AACCTAA | 1 | MYB binding site involved in light responsiveness |
| BoRR8.2 | CAT-box | GCCACT | 1 | cis-acting regulatory element related to meristem expression |
| BoRR8.2 | Box 4 | ATTAAT | 2 | part of a conserved DNA module involved in light responsiveness |
| BoRR9.1 | G-Box | CACGTT | 1 | cis-acting regulatory element involved in light responsiveness |
| BoRR9.1 | ABRE | GACACGTGGC | 2 | cis-acting element involved in the abscisic acid responsiveness |
| BoRR9.1 | TCT-motif | TCTTAC | 1 | part of a light responsive element |
| BoRR9.1 | TC-rich repeats | ATTCTCTAAC | 1 | cis-acting element involved in defense and stress responsiveness |
| BoRR9.1 | GATA-motif | AAGGATAAGG | 2 | part of a light responsive element |
| BoRR9.1 | ARE | AAACCA | 1 | cis-acting regulatory element essential for the anaerobic induction |
| BoRR9.1 | MRE | AACCTAA | 2 | MYB binding site involved in light responsiveness |
| BoRR9.1 | I-box | cGATAAGGCG | 1 | part of a light responsive element |
| BoRR9.1 | GT1-motif | GGTTAA | 2 | light responsive element |
| BoRR9.1 | TGACG-motif | TGACG | 3 | cis-acting regulatory element involved in the MeJA-responsiveness |
| BoRR9.1 | chs-CMA1a | TTACTTAA | 1 | part of a light responsive element |
| BoRR9.1 | circadian | CAAAGATATC | 1 | cis-acting regulatory element involved in circadian control |
| BoRR9.1 | MBS | CAACTG | 2 | MYB binding site involved in drought-inducibility |
| BoRR9.1 | CGTCA-motif | CGTCA | 3 | cis-acting regulatory element involved in the MeJA-responsiveness |
| BoRR9.2 | GARE-motif | TCTGTTG | 1 | gibberellin-responsive element |
| BoRR9.2 | ARE | AAACCA | 4 | cis-acting regulatory element essential for the anaerobic induction |
| BoRR9.2 | CGTCA-motif | CGTCA | 1 | cis-acting regulatory element involved in the MeJA-responsiveness |
| BoRR9.2 | O2-site | GATGA(C/T)(A/G)TG(A/G) | 2 | cis-acting regulatory element involved in zein metabolism regulation |
| BoRR9.2 | GATA-motif | GATAGGG | 1 | part of a light responsive element |
| BoRR9.2 | Box 4 | ATTAAT | 1 | part of a conserved DNA module involved in light responsiveness |
| BoRR9.2 | chs-CMA1a | TTACTTAA | 1 | part of a light responsive element |
| BoRR9.2 | MRE | AACCTAA | 2 | MYB binding site involved in light responsiveness |
| BoRR9.2 | TATC-box | TATCCCA | 1 | cis-acting element involved in gibberellin-responsiveness |
| BoRR9.2 | ATC-motif | AGTAATCT | 1 | part of a conserved DNA module involved in light responsiveness |
| BoRR9.2 | CAG-motif | GAAAGGCAGAC | 1 | part of a light response element |
| BoRR9.2 | GT1-motif | GGTTAA | 2 | light responsive element |
| BoRR9.2 | AE-box | AGAAACAA | 1 | part of a module for light response |
| BoRR9.2 | TGACG-motif | TGACG | 1 | cis-acting regulatory element involved in the MeJA-responsiveness |
| BoRR10.1 | ABRE | ACGTG | 2 | cis-acting element involved in the abscisic acid responsiveness |
| BoRR10.1 | TGA-element | AACGAC | 3 | auxin-responsive element |
| BoRR10.1 | CGTCA-motif | CGTCA | 1 | cis-acting regulatory element involved in the MeJA-responsiveness |
| BoRR10.1 | circadian | CAAAGATATC | 1 | cis-acting regulatory element involved in circadian control |
| BoRR10.1 | O2-site | GATGATGTGG | 1 | cis-acting regulatory element involved in zein metabolism regulation |
| BoRR10.1 | Pc-CMA2c | GCCCACGCA | 1 | part of a light responsive element |
| BoRR10.1 | MBS | CAACTG | 2 | MYB binding site involved in drought-inducibility |
| BoRR10.1 | ARE | AAACCA | 6 | cis-acting regulatory element essential for the anaerobic induction |
| BoRR10.1 | GATA-motif | GATAGGG | 3 | part of a light responsive element |
| BoRR10.1 | GT1-motif | GGTTAAT | 2 | light responsive element |
| BoRR10.1 | TC-rich repeats | ATTCTCTAAC | 1 | cis-acting element involved in defense and stress responsiveness |
| BoRR10.1 | G-box | CCACGTAA | 3 | cis-acting regulatory element involved in light responsiveness |
| BoRR10.1 | Box 4 | ATTAAT | 4 | part of a conserved DNA module involved in light responsiveness |
| BoRR10.1 | TCT-motif | TCTTAC | 2 | part of a light responsive element |
| BoRR10.1 | TGACG-motif | TGACG | 1 | cis-acting regulatory element involved in the MeJA-responsiveness |
| BoRR10.1 | sbp-CMA1c | CTTTATCTCTTCCA | 1 | part of a light responsive element |
| BoRR10.1 | I-box | gGATAAGGTG | 1 | part of a light responsive element |
| BoRR10.2 | I-box | cGATAAGGCG | 1 | part of a light responsive element |
| BoRR10.2 | Box II | TGGTAATAA | 1 | part of a light responsive element |
| BoRR10.2 | MBS | CAACTG | 1 | MYB binding site involved in drought-inducibility |
| BoRR10.2 | TCT-motif | TCTTAC | 2 | part of a light responsive element |
| BoRR10.2 | LTR | CCGAAA | 3 | cis-acting element involved in low-temperature responsiveness |
| BoRR10.2 | ARE | AAACCA | 5 | cis-acting regulatory element essential for the anaerobic induction |
| BoRR10.2 | CGTCA-motif | CGTCA | 1 | cis-acting regulatory element involved in the MeJA-responsiveness |
| BoRR10.2 | TGA-element | AACGAC | 1 | auxin-responsive element |
| BoRR10.2 | Box 4 | ATTAAT | 7 | part of a conserved DNA module involved in light responsiveness |
| BoRR10.2 | TGACG-motif | TGACG | 1 | cis-acting regulatory element involved in the MeJA-responsiveness |
| BoRR10.2 | P-box | CCTTTTG | 1 | gibberellin-responsive element |
| BoRR10.2 | GT1-motif | GGTTAA | 3 | light responsive element |
| BoRR10.2 | chs-CMA1a | TTACTTAA | 1 | part of a light responsive element |
| BoRR11 | O2-site | GATGACATGG | 4 | cis-acting regulatory element involved in zein metabolism regulation |
| BoRR11 | ARE | AAACCA | 1 | cis-acting regulatory element essential for the anaerobic induction |
| BoRR11 | ATCT-motif | AATCTAATCC | 1 | part of a conserved DNA module involved in light responsiveness |
| BoRR11 | Box II | TGGTAATAA | 2 | part of a light responsive element |
| BoRR11 | Box 4 | ATTAAT | 1 | part of a conserved DNA module involved in light responsiveness |
| BoRR11 | LTR | CCGAAA | 3 | cis-acting element involved in low-temperature responsiveness |
| BoRR11 | TCT-motif | TCTTAC | 1 | part of a light responsive element |
| BoRR11 | ACE | CTAACGTATT | 2 | cis-acting element involved in light responsiveness |
| BoRR12 | TCT-motif | TCTTAC | 1 | part of a light responsive element |
| BoRR12 | AT1-motif | AATTATTTTTTATT | 1 | part of a light responsive module |
| BoRR12 | GT1-motif | GGTTAA | 2 | light responsive element |
| BoRR12 | TGACG-motif | TGACG | 2 | cis-acting regulatory element involved in the MeJA-responsiveness |
| BoRR12 | CGTCA-motif | CGTCA | 2 | cis-acting regulatory element involved in the MeJA-responsiveness |
| BoRR12 | GARE-motif | TCTGTTG | 1 | gibberellin-responsive element |
| BoRR12 | TC-rich repeats | ATTCTCTAAC | 2 | cis-acting element involved in defense and stress responsiveness |
| BoRR12 | LTR | CCGAAA | 1 | cis-acting element involved in low-temperature responsiveness |
| BoRR12 | Box 4 | ATTAAT | 1 | part of a conserved DNA module involved in light responsiveness |
| BoRR12 | GATA-motif | AAGGATAAGG | 1 | part of a light responsive element |
| BoRR12 | MBS | CAACTG | 1 | MYB binding site involved in drought-inducibility |
| BoRR13 | ABRE | CGTACGTGCA | 3 | cis-acting element involved in the abscisic acid responsiveness |
| BoRR13 | TC-rich repeats | ATTCTCTAAC | 2 | cis-acting element involved in defense and stress responsiveness |
| BoRR13 | GT1-motif | GGTTAA | 1 | light responsive element |
| BoRR13 | TGA-element | AACGAC | 1 | auxin-responsive element |
| BoRR13 | ARE | AAACCA | 1 | cis-acting regulatory element essential for the anaerobic induction |
| BoRR13 | Box 4 | ATTAAT | 3 | part of a conserved DNA module involved in light responsiveness |
| BoRR13 | chs-CMA1a | TTACTTAA | 1 | part of a light responsive element |
| BoRR13 | I-box | atGATAAGGTC | 1 | part of a light responsive element |
| BoRR13 | TCT-motif | TCTTAC | 1 | part of a light responsive element |
| BoRR13 | GA-motif | ATAGATAA | 2 | part of a light responsive element |
| BoRR13 | G-box | CACGAC | 3 | cis-acting regulatory element involved in light responsiveness |
| BoRR14 | G-box | CACGAC | 1 | cis-acting regulatory element involved in light responsiveness |
| BoRR14 | TC-rich repeats | ATTCTCTAAC | 1 | cis-acting element involved in defense and stress responsiveness |
| BoRR14 | GATA-motif | AAGATAAGATT | 1 | part of a light responsive element |
| BoRR14 | MRE | AACCTAA | 1 | MYB binding site involved in light responsiveness |
| BoRR14 | TCA-element | CCATCTTTTT | 1 | cis-acting element involved in salicylic acid responsiveness |
| BoRR14 | ATC-motif | AGTAATCT | 1 | part of a conserved DNA module involved in light responsiveness |
| BoRR14 | I-box | TGATAATGT | 1 | part of a light responsive element |
| BoRR14 | ARE | AAACCA | 4 | cis-acting regulatory element essential for the anaerobic induction |
| BoRR14 | GA-motif | ATAGATAA | 1 | part of a light responsive element |
| BoRR14 | MBSI | aaaAaaC(G/C)GTTA | 1 | MYB binding site involved in flavonoid biosynthetic genes regulation |
| BoRR14 | ABRE | AACCCGG | 1 | cis-acting element involved in the abscisic acid responsiveness |
| BoRR14 | TCT-motif | TCTTAC | 2 | part of a light responsive element |
| BoRR15.1 | G-box | CACGTC | 1 | cis-acting regulatory element involved in light responsiveness |
| BoRR15.1 | CGTCA-motif | CGTCA | 1 | cis-acting regulatory element involved in the MeJA-responsiveness |
| BoRR15.1 | TGACG-motif | TGACG | 1 | cis-acting regulatory element involved in the MeJA-responsiveness |
| BoRR15.1 | TCT-motif | TCTTAC | 4 | part of a light responsive element |
| BoRR15.1 | GCN4_motif | TGAGTCA | 1 | cis-regulatory element involved in endosperm expression |
| BoRR15.1 | Box 4 | ATTAAT | 1 | part of a conserved DNA module involved in light responsiveness |
| BoRR15.1 | I-box | AGATAAGG | 1 | part of a light responsive element |
| BoRR15.1 | P-box | CCTTTTG | 1 | gibberellin-responsive element |
| BoRR15.1 | MSA-like | TCAAACGGT | 1 | cis-acting element involved in cell cycle regulation |
| BoRR15.1 | TCCC-motif | TCTCCCT | 1 | part of a light responsive element |
| BoRR15.1 | ARE | AAACCA | 1 | cis-acting regulatory element essential for the anaerobic induction |
| BoRR15.1 | ABRE | ACGTG | 1 | cis-acting element involved in the abscisic acid responsiveness |
| BoRR15.1 | MBS | CAACTG | 2 | MYB binding site involved in drought-inducibility |
| BoRR15.2 | MRE | AACCTAA | 1 | MYB binding site involved in light responsiveness |
| BoRR15.2 | ABRE | ACGTG | 2 | cis-acting element involved in the abscisic acid responsiveness |
| BoRR15.2 | Gap-box | CAAATGAA(A/G)A | 1 | part of a light responsive element |
| BoRR15.2 | G-box | CCACGTAA | 2 | cis-acting regulatory element involved in light responsiveness |
| BoRR15.2 | TATC-box | TATCCCA | 1 | cis-acting element involved in gibberellin-responsiveness |
| BoRR15.2 | ACE | GACACGTATG | 1 | cis-acting element involved in light responsiveness |
| BoRR15.2 | TGACG-motif | TGACG | 1 | cis-acting regulatory element involved in the MeJA-responsiveness |
| BoRR15.2 | CGTCA-motif | CGTCA | 1 | cis-acting regulatory element involved in the MeJA-responsiveness |
| BoRR15.2 | AE-box | AGAAACTT | 1 | part of a module for light response |
| BoRR15.2 | LTR | CCGAAA | 1 | cis-acting element involved in low-temperature responsiveness |
| BoRR15.2 | GARE-motif | TCTGTTG | 1 | gibberellin-responsive element |
| BoRR15.2 | ATCT-motif | AATCTAATCC | 1 | part of a conserved DNA module involved in light responsiveness |
| BoRR15.2 | TCT-motif | TCTTAC | 1 | part of a light responsive element |
| BoRR15.2 | MBS | CAACTG | 2 | MYB binding site involved in drought-inducibility |
| BoRR15.2 | GATA-motif | AAGATAAGATT | 1 | part of a light responsive element |
| BoRR16 | TCT-motif | TCTTAC | 2 | part of a light responsive element |
| BoRR16 | AE-box | AGAAACTT | 1 | part of a module for light response |
| BoRR16 | CGTCA-motif | CGTCA | 1 | cis-acting regulatory element involved in the MeJA-responsiveness |
| BoRR16 | MRE | AACCTAA | 1 | MYB binding site involved in light responsiveness |
| BoRR16 | HD-Zip 1 | CAAT(A/T)ATTG | 1 | element involved in differentiation of the palisade mesophyll cells |
| BoRR16 | I-box | cCATATCCAAT | 1 | part of a light responsive element |
| BoRR16 | ABRE | ACGTG | 1 | cis-acting element involved in the abscisic acid responsiveness |
| BoRR16 | ARE | AAACCA | 8 | cis-acting regulatory element essential for the anaerobic induction |
| BoRR16 | TGACG-motif | TGACG | 1 | cis-acting regulatory element involved in the MeJA-responsiveness |
| BoRR16 | G-box | TACGTG | 1 | cis-acting regulatory element involved in light responsiveness |
| BoRR16 | Box 4 | ATTAAT | 1 | part of a conserved DNA module involved in light responsiveness |
| BoRR16 | WUN-motif | AAATTTCCT | 1 | wound-responsive element |
| BoRR16 | Gap-box | CAAATGAA(A/G)A | 1 | part of a light responsive element |
| BoRR16 | LTR | CCGAAA | 2 | cis-acting element involved in low-temperature responsiveness |
| BoRR16 | TC-rich repeats | GTTTTCTTAC | 1 | cis-acting element involved in defense and stress responsiveness |
| BoRR17.1 | ATCT-motif | AATCTAATCC | 1 | part of a conserved DNA module involved in light responsiveness |
| BoRR17.1 | GT1-motif | GGTTAA | 3 | light responsive element |
| BoRR17.1 | TCT-motif | TCTTAC | 2 | part of a light responsive element |
| BoRR17.1 | MRE | AACCTAA | 1 | MYB binding site involved in light responsiveness |
| BoRR17.1 | LAMP-element | CTTTATCA | 1 | part of a light responsive element |
| BoRR17.1 | TGACG-motif | TGACG | 1 | cis-acting regulatory element involved in the MeJA-responsiveness |
| BoRR17.1 | AE-box | AGAAACTT | 2 | part of a module for light response |
| BoRR17.1 | MBS | CAACTG | 2 | MYB binding site involved in drought-inducibility |
| BoRR17.1 | Box 4 | ATTAAT | 2 | part of a conserved DNA module involved in light responsiveness |
| BoRR17.1 | CGTCA-motif | CGTCA | 1 | cis-acting regulatory element involved in the MeJA-responsiveness |
| BoRR17.1 | G-box | CACGTC | 1 | cis-acting regulatory element involved in light responsiveness |
| BoRR17.1 | ABRE | ACGTG | 1 | cis-acting element involved in the abscisic acid responsiveness |
| BoRR17.1 | ARE | AAACCA | 14 | cis-acting regulatory element essential for the anaerobic induction |
| BoRR17.1 | Gap-box | CAAATGAA(A/G)A | 1 | part of a light responsive element |
| BoRR17.1 | GA-motif | ATAGATAA | 2 | part of a light responsive element |
| BoRR17.1 | TC-rich repeats | GTTTTCTTAC | 1 | cis-acting element involved in defense and stress responsiveness |
| BoRR17.2 | GT1-motif | GGTTAA | 4 | light responsive element |
| BoRR17.2 | ABRE | AACCCGG | 3 | cis-acting element involved in the abscisic acid responsiveness |
| BoRR17.2 | Box 4 | ATTAAT | 2 | part of a conserved DNA module involved in light responsiveness |
| BoRR17.2 | CGTCA-motif | CGTCA | 1 | cis-acting regulatory element involved in the MeJA-responsiveness |
| BoRR17.2 | AE-box | AGAAACTT | 2 | part of a module for light response |
| BoRR17.2 | G-box | CACGTC | 1 | cis-acting regulatory element involved in light responsiveness |
| BoRR17.2 | ARE | AAACCA | 1 | cis-acting regulatory element essential for the anaerobic induction |
| BoRR17.2 | G-Box | CACGTT | 1 | cis-acting regulatory element involved in light responsiveness |
| BoRR17.2 | TCA-element | CCATCTTTTT | 1 | cis-acting element involved in salicylic acid responsiveness |
| BoRR17.2 | TATC-box | TATCCCA | 1 | cis-acting element involved in gibberellin-responsiveness |
| BoRR17.2 | MBS | CAACTG | 1 | MYB binding site involved in drought-inducibility |
| BoRR17.2 | TGACG-motif | TGACG | 1 | cis-acting regulatory element involved in the MeJA-responsiveness |
| BoRR17.2 | GA-motif | ATAGATAA | 1 | part of a light responsive element |
| BoRR17.2 | MRE | AACCTAA | 1 | MYB binding site involved in light responsiveness |
| BoRR17.2 | Gap-box | CAAATGAA(A/G)A | 1 | part of a light responsive element |
| BoRR17.2 | AuxRR-core | GGTCCAT | 1 | cis-acting regulatory element involved in auxin responsiveness |
| BoRR18 | TGA-element | AACGAC | 2 | auxin-responsive element |
| BoRR18 | ABRE | ACGTG | 1 | cis-acting element involved in the abscisic acid responsiveness |
| BoRR18 | GARE-motif | TCTGTTG | 1 | gibberellin-responsive element |
| BoRR18 | TC-rich repeats | ATTCTCTAAC | 1 | cis-acting element involved in defense and stress responsiveness |
| BoRR18 | CGTCA-motif | CGTCA | 2 | cis-acting regulatory element involved in the MeJA-responsiveness |
| BoRR18 | ARE | AAACCA | 3 | cis-acting regulatory element essential for the anaerobic induction |
| BoRR18 | TCA-element | CCATCTTTTT | 2 | cis-acting element involved in salicylic acid responsiveness |
| BoRR18 | G-Box | CACGTT | 1 | cis-acting regulatory element involved in light responsiveness |
| BoRR18 | TGACG-motif | TGACG | 2 | cis-acting regulatory element involved in the MeJA-responsiveness |
| BoRR18 | P-box | CCTTTTG | 1 | gibberellin-responsive element |
| BoRR18 | circadian | CAAAGATATC | 2 | cis-acting regulatory element involved in circadian control |
| BoRR19.1 | LTR | CCGAAA | 2 | cis-acting element involved in low-temperature responsiveness |
| BoRR19.1 | MBS | CAACTG | 2 | MYB binding site involved in drought-inducibility |
| BoRR19.1 | TGACG-motif | TGACG | 2 | cis-acting regulatory element involved in the MeJA-responsiveness |
| BoRR19.1 | TCT-motif | TCTTAC | 2 | part of a light responsive element |
| BoRR19.1 | ARE | AAACCA | 5 | cis-acting regulatory element essential for the anaerobic induction |
| BoRR19.1 | MRE | AACCTAA | 1 | MYB binding site involved in light responsiveness |
| BoRR19.1 | CGTCA-motif | CGTCA | 2 | cis-acting regulatory element involved in the MeJA-responsiveness |
| BoRR19.1 | Box 4 | ATTAAT | 5 | part of a conserved DNA module involved in light responsiveness |
| BoRR19.2 | TCA-element | CCATCTTTTT | 1 | cis-acting element involved in salicylic acid responsiveness |
| BoRR19.2 | RY-element | CATGCATG | 1 | cis-acting regulatory element involved in seed-specific regulation |
| BoRR19.2 | MBS | CAACTG | 1 | MYB binding site involved in drought-inducibility |
| BoRR19.2 | TCT-motif | TCTTAC | 2 | part of a light responsive element |
| BoRR19.2 | MRE | AACCTAA | 2 | MYB binding site involved in light responsiveness |
| BoRR19.2 | G-Box | CACGTT | 1 | cis-acting regulatory element involved in light responsiveness |
| BoRR19.2 | TGA-element | AACGAC | 3 | auxin-responsive element |
| BoRR19.2 | ARE | AAACCA | 5 | cis-acting regulatory element essential for the anaerobic induction |
| BoRR19.2 | ABRE | ACGTG | 3 | cis-acting element involved in the abscisic acid responsiveness |
| BoRR19.2 | TGACG-motif | TGACG | 5 | cis-acting regulatory element involved in the MeJA-responsiveness |
| BoRR19.2 | chs-CMA2a | TCACTTGA | 1 | part of a light responsive element |
| BoRR19.2 | GT1-motif | GGTTAAT | 3 | light responsive element |
| BoRR19.2 | CGTCA-motif | CGTCA | 5 | cis-acting regulatory element involved in the MeJA-responsiveness |
| BoRR19.2 | G-box | CACGTC | 3 | cis-acting regulatory element involved in light responsiveness |
| BoRR19.2 | LTR | CCGAAA | 1 | cis-acting element involved in low-temperature responsiveness |
| BoRR19.2 | TC-rich repeats | GTTTTCTTAC | 1 | cis-acting element involved in defense and stress responsiveness |
| BoRR20 | GCN4_motif | TGAGTCA | 1 | cis-regulatory element involved in endosperm expression |
| BoRR20 | AuxRR-core | GGTCCAT | 1 | cis-acting regulatory element involved in auxin responsiveness |
| BoRR20 | LTR | CCGAAA | 1 | cis-acting element involved in low-temperature responsiveness |
| BoRR20 | circadian | CAAAGATATC | 1 | cis-acting regulatory element involved in circadian control |
| BoRR20 | P-box | CCTTTTG | 1 | gibberellin-responsive element |
| BoRR20 | ABRE | ACGTG | 1 | cis-acting element involved in the abscisic acid responsiveness |
| BoRR20 | GARE-motif | TCTGTTG | 1 | gibberellin-responsive element |
| BoRR20 | CGTCA-motif | CGTCA | 1 | cis-acting regulatory element involved in the MeJA-responsiveness |
| BoRR20 | TGACG-motif | TGACG | 1 | cis-acting regulatory element involved in the MeJA-responsiveness |
| BoRR20 | ARE | AAACCA | 3 | cis-acting regulatory element essential for the anaerobic induction |
| BoRR20 | GT1-motif | GGTTAA | 1 | light responsive element |
| BoRR20 | TC-rich repeats | GTTTTCTTAC | 1 | cis-acting element involved in defense and stress responsiveness |
| BoRR20 | G-box | TACGTG | 1 | cis-acting regulatory element involved in light responsiveness |
| BoRR20 | ACE | CTAACGTATT | 1 | cis-acting element involved in light responsiveness |
| BoRR20 | TCA-element | TCAGAAGAGG | 2 | cis-acting element involved in salicylic acid responsiveness |
| BoRR20 | chs-CMA2a | TCACTTGA | 1 | part of a light responsive element |
| BoRR21 | AE-box | AGAAACAA | 1 | part of a module for light response |
| BoRR21 | GCN4_motif | TGAGTCA | 1 | cis-regulatory element involved in endosperm expression |
| BoRR21 | GARE-motif | TCTGTTG | 1 | gibberellin-responsive element |
| BoRR21 | G-box | TACGTG | 5 | cis-acting regulatory element involved in light responsiveness |
| BoRR21 | MBS | CAACTG | 1 | MYB binding site involved in drought-inducibility |
| BoRR21 | ARE | AAACCA | 3 | cis-acting regulatory element essential for the anaerobic induction |
| BoRR21 | P-box | CCTTTTG | 1 | gibberellin-responsive element |
| BoRR21 | Box 4 | ATTAAT | 3 | part of a conserved DNA module involved in light responsiveness |
| BoRR21 | Unnamed_1 | GGATTTTACAGT | 1 | cis-acting element involved in phytochrome down-regulation expression |
| BoRR21 | CGTCA-motif | CGTCA | 1 | cis-acting regulatory element involved in the MeJA-responsiveness |
| BoRR21 | CAAT-box | CAAAT | 1 | common cis-acting element in promoter and enhancer regions |
| BoRR21 | LAMP-element | CTTTATCA | 1 | part of a light responsive element |
| BoRR21 | TC-rich repeats | GTTTTCTTAC | 1 | cis-acting element involved in defense and stress responsiveness |
| BoRR21 | TATC-box | TATCCCA | 2 | cis-acting element involved in gibberellin-responsiveness |
| BoRR21 | TCT-motif | TCTTAC | 3 | part of a light responsive element |
| BoRR21 | ABRE | ACGTG | 4 | cis-acting element involved in the abscisic acid responsiveness |
| BoRR21 | TGACG-motif | TGACG | 1 | cis-acting regulatory element involved in the MeJA-responsiveness |
| BoRR22.1 | LAMP-element | CTTTATCA | 1 | part of a light responsive element |
| BoRR22.1 | O2-site | GTTGACGTGA | 1 | cis-acting regulatory element involved in zein metabolism regulation |
| BoRR22.1 | TGACG-motif | TGACG | 2 | cis-acting regulatory element involved in the MeJA-responsiveness |
| BoRR22.1 | TCT-motif | TCTTAC | 1 | part of a light responsive element |
| BoRR22.1 | MBS | CAACTG | 1 | MYB binding site involved in drought-inducibility |
| BoRR22.1 | G-box | CACGTC | 1 | cis-acting regulatory element involved in light responsiveness |
| BoRR22.1 | LTR | CCGAAA | 1 | cis-acting element involved in low-temperature responsiveness |
| BoRR22.1 | GT1-motif | GGTTAAT | 3 | light responsive element |
| BoRR22.1 | I-box | gGATAAGGTG | 1 | part of a light responsive element |
| BoRR22.1 | ARE | AAACCA | 1 | cis-acting regulatory element essential for the anaerobic induction |
| BoRR22.1 | TCA-element | CCATCTTTTT | 2 | cis-acting element involved in salicylic acid responsiveness |
| BoRR22.1 | CGTCA-motif | CGTCA | 2 | cis-acting regulatory element involved in the MeJA-responsiveness |
| BoRR22.1 | ATC-motif | AGTAATCT | 1 | part of a conserved DNA module involved in light responsiveness |
| BoRR22.1 | Box 4 | ATTAAT | 6 | part of a conserved DNA module involved in light responsiveness |
| BoRR22.1 | ABRE | ACGTG | 3 | cis-acting element involved in the abscisic acid responsiveness |
| BoRR22.1 | G-Box | CACGTT | 1 | cis-acting regulatory element involved in light responsiveness |
| BoRR22.1 | GA-motif | ATAGATAA | 1 | part of a light responsive element |
| BoRR22.1 | ACE | CTAACGTATT | 1 | cis-acting element involved in light responsiveness |
| BoRR22.2 | TGA-element | AACGAC | 1 | auxin-responsive element |
| BoRR22.2 | TC-rich repeats | GTTTTCTTAC | 1 | cis-acting element involved in defense and stress responsiveness |
| BoRR22.2 | CGTCA-motif | CGTCA | 4 | cis-acting regulatory element involved in the MeJA-responsiveness |
| BoRR22.2 | GATA-motif | GATAGGA | 1 | part of a light responsive element |
| BoRR22.2 | ARE | AAACCA | 7 | cis-acting regulatory element essential for the anaerobic induction |
| BoRR22.2 | GARE-motif | TCTGTTG | 1 | gibberellin-responsive element |
| BoRR22.2 | ABRE | ACGTG | 2 | cis-acting element involved in the abscisic acid responsiveness |
| BoRR22.2 | LAMP-element | CTTTATCA | 1 | part of a light responsive element |
| BoRR22.2 | TCA-element | TCAGAAGAGG | 1 | cis-acting element involved in salicylic acid responsiveness |
| BoRR22.2 | TGACG-motif | TGACG | 4 | cis-acting regulatory element involved in the MeJA-responsiveness |
| BoRR22.2 | G-box | CACGTC | 1 | cis-acting regulatory element involved in light responsiveness |
| BoRR22.2 | GT1-motif | GGTTAA | 1 | light responsive element |
| BoRR23 | GATA-motif | AAGATAAGATT | 1 | part of a light responsive element |
| BoRR23 | ARE | AAACCA | 5 | cis-acting regulatory element essential for the anaerobic induction |
| BoRR23 | ABRE | CACGTG | 2 | cis-acting element involved in the abscisic acid responsiveness |
| BoRR23 | TCA-element | CCATCTTTTT | 1 | cis-acting element involved in salicylic acid responsiveness |
| BoRR23 | G-Box | CACGTG | 1 | cis-acting regulatory element involved in light responsiveness |
| BoRR23 | MBSI | TTTTTACGGTTA | 1 | MYB binding site involved in flavonoid biosynthetic genes regulation |
| BoRR23 | P-box | CCTTTTG | 1 | gibberellin-responsive element |
| BoRR23 | Box 4 | ATTAAT | 6 | part of a conserved DNA module involved in light responsiveness |
| BoRR23 | AE-box | AGAAACAA | 2 | part of a module for light response |
| BoRR23 | CAT-box | GCCACT | 1 | cis-acting regulatory element related to meristem expression |
| BoRR23 | TCT-motif | TCTTAC | 5 | part of a light responsive element |
| BoRR23 | GT1-motif | GGTTAAT | 1 | light responsive element |
| BoRR23 | G-box | CACGTG | 2 | cis-acting regulatory element involved in light responsiveness |
| BoRR24.1 | TC-rich repeats | GTTTTCTTAC | 1 | cis-acting element involved in defense and stress responsiveness |
| BoRR24.1 | TGACG-motif | TGACG | 3 | cis-acting regulatory element involved in the MeJA-responsiveness |
| BoRR24.1 | CGTCA-motif | CGTCA | 3 | cis-acting regulatory element involved in the MeJA-responsiveness |
| BoRR24.1 | ABRE | ACGTG | 4 | cis-acting element involved in the abscisic acid responsiveness |
| BoRR24.1 | G-Box | CACGTT | 5 | cis-acting regulatory element involved in light responsiveness |
| BoRR24.1 | AE-box | AGAAACAA | 1 | part of a module for light response |
| BoRR24.1 | chs-CMA2a | TCACTTGA | 1 | part of a light responsive element |
| BoRR24.1 | TCT-motif | TCTTAC | 2 | part of a light responsive element |
| BoRR24.1 | Box 4 | ATTAAT | 2 | part of a conserved DNA module involved in light responsiveness |
| BoRR24.1 | LTR | CCGAAA | 1 | cis-acting element involved in low-temperature responsiveness |
| BoRR24.1 | GATA-motif | AAGATAAGATT | 1 | part of a light responsive element |
| BoRR24.1 | ARE | AAACCA | 3 | cis-acting regulatory element essential for the anaerobic induction |
| BoRR24.1 | Gap-box | CAAATGAA(A/G)A | 1 | part of a light responsive element |
| BoRR24.1 | GT1-motif | GGTTAA | 2 | light responsive element |
| BoRR24.1 | MBS | CAACTG | 1 | MYB binding site involved in drought-inducibility |
| BoRR24.2 | CGTCA-motif | CGTCA | 2 | cis-acting regulatory element involved in the MeJA-responsiveness |
| BoRR24.2 | GA-motif | ATAGATAA | 1 | part of a light responsive element |
| BoRR24.2 | LTR | CCGAAA | 1 | cis-acting element involved in low-temperature responsiveness |
| BoRR24.2 | G-box | ACACGTGT | 9 | cis-acting regulatory element involved in light responsiveness |
| BoRR24.2 | O2-site | GATGACATGG | 2 | cis-acting regulatory element involved in zein metabolism regulation |
| BoRR24.2 | TCCC-motif | TCTCCCT | 1 | part of a light responsive element |
| BoRR24.2 | MRE | AACCTAA | 1 | MYB binding site involved in light responsiveness |
| BoRR24.2 | TGACG-motif | TGACG | 2 | cis-acting regulatory element involved in the MeJA-responsiveness |
| BoRR24.2 | GARE-motif | TCTGTTG | 1 | gibberellin-responsive element |
| BoRR24.2 | TATC-box | TATCCCA | 1 | cis-acting element involved in gibberellin-responsiveness |
| BoRR24.2 | ABRE | CACGTG | 9 | cis-acting element involved in the abscisic acid responsiveness |
| BoRR24.2 | ARE | AAACCA | 5 | cis-acting regulatory element essential for the anaerobic induction |
| BoRR24.2 | AE-box | AGAAACTT | 2 | part of a module for light response |
| BoRR24.2 | TGA-element | AACGAC | 1 | auxin-responsive element |
| BoRR24.2 | TC-rich repeats | ATTCTCTAAC | 2 | cis-acting element involved in defense and stress responsiveness |
| BoRR24.2 | TCT-motif | TCTTAC | 1 | part of a light responsive element |
| BoRR24.2 | TCA-element | CCATCTTTTT | 1 | cis-acting element involved in salicylic acid responsiveness |
| BoRR24.3 | LAMP-element | CTTTATCA | 1 | part of a light responsive element |
| BoRR24.3 | Box 4 | ATTAAT | 2 | part of a conserved DNA module involved in light responsiveness |
| BoRR24.3 | ARE | AAACCA | 7 | cis-acting regulatory element essential for the anaerobic induction |
| BoRR24.3 | LTR | CCGAAA | 7 | cis-acting element involved in low-temperature responsiveness |
| BoRR24.3 | TCA-element | CCATCTTTTT | 1 | cis-acting element involved in salicylic acid responsiveness |
| BoRR24.3 | GT1-motif | GGTTAA | 4 | light responsive element |
| BoRR24.3 | MBS | CAACTG | 3 | MYB binding site involved in drought-inducibility |
| BoRR24.3 | TC-rich repeats | GTTTTCTTAC | 1 | cis-acting element involved in defense and stress responsiveness |
| BoRR24.3 | GATA-motif | AAGATAAGATT | 1 | part of a light responsive element |
| BoRR24.3 | MBSI | aaaAaaC(G/C)GTTA | 1 | MYB binding site involved in flavonoid biosynthetic genes regulation |
| BoRR24.3 | P-box | CCTTTTG | 1 | gibberellin-responsive element |
| BoRR24.3 | circadian | CAAAGATATC | 1 | cis-acting regulatory element involved in circadian control |
| BoRR24.3 | AE-box | AGAAACAA | 1 | part of a module for light response |
| BoRR24.3 | ABRE | ACGTG | 1 | cis-acting element involved in the abscisic acid responsiveness |
| BoRR24.3 | G-Box | CACGTT | 2 | cis-acting regulatory element involved in light responsiveness |
| BoRR24.3 | ATCT-motif | AATCTAATCC | 2 | part of a conserved DNA module involved in light responsiveness |
| BoPRR1 | ABRE | ACGTG | 1 | cis-acting element involved in the abscisic acid responsiveness |
| BoPRR1 | Sp1 | GGGCGG | 3 | light responsive element |
| BoPRR1 | Sp1 | GGGCGG |  | light responsive element |
| BoPRR1 | Sp1 | GGGCGG |  | light responsive element |
| BoPRR1 | ARE | AAACCA | 1 | cis-acting regulatory element essential for the anaerobic induction |
| BoPRR1 | CAT-box | GCCACT | 1 | cis-acting regulatory element related to meristem expression |
| BoPRR1 | GC-motif | CCCCCG | 4 | enhancer-like element involved in anoxic specific inducibility |
| BoPRR1 | G-box | TACGTG | 4 | cis-acting regulatory element involved in light responsiveness |
| BoPRR2.1 | TC-rich repeats | ATTCTCTAAC | 1 | cis-acting element involved in defense and stress responsiveness |
| BoPRR2.1 | ARE | AAACCA | 4 | cis-acting regulatory element essential for the anaerobic induction |
| BoPRR2.1 | WUN-motif | AAATTTCCT | 1 | wound-responsive element |
| BoPRR2.1 | Box 4 | ATTAAT | 4 | part of a conserved DNA module involved in light responsiveness |
| BoPRR2.1 | AE-box | AGAAACAA | 2 | part of a module for light response |
| BoPRR2.1 | TCA-element | CCATCTTTTT | 1 | cis-acting element involved in salicylic acid responsiveness |
| BoPRR2.1 | CAT-box | GCCACT | 1 | cis-acting regulatory element related to meristem expression |
| BoPRR2.1 | AAAC-motif | CAATCAAAACCT | 1 | light responsive element |
| BoPRR2.2 | TGA-element | AACGAC | 1 | auxin-responsive element |
| BoPRR2.2 | LTR | CCGAAA | 3 | cis-acting element involved in low-temperature responsiveness |
| BoPRR2.2 | G-Box | CACGTG | 2 | cis-acting regulatory element involved in light responsiveness |
| BoPRR2.2 | TGACG-motif | TGACG | 1 | cis-acting regulatory element involved in the MeJA-responsiveness |
| BoPRR2.2 | CGTCA-motif | CGTCA | 1 | cis-acting regulatory element involved in the MeJA-responsiveness |
| BoPRR2.2 | TCCC-motif | TCTCCCT | 1 | part of a light responsive element |
| BoPRR2.2 | GT1-motif | GGTTAA | 1 | light responsive element |
| BoPRR2.2 | Box 4 | ATTAAT | 6 | part of a conserved DNA module involved in light responsiveness |
| BoPRR2.2 | MRE | AACCTAA | 2 | MYB binding site involved in light responsiveness |
| BoPRR2.2 | G-box | ACACGTGGC | 3 | cis-acting regulatory element involved in light responsiveness |
| BoPRR2.2 | GARE-motif | TCTGTTG | 1 | gibberellin-responsive element |
| BoPRR2.2 | TCA-element | CCATCTTTTT | 1 | cis-acting element involved in salicylic acid responsiveness |
| BoPRR2.2 | circadian | CAAAGATATC | 1 | cis-acting regulatory element involved in circadian control |
| BoPRR2.2 | ABRE | GACACGTGGC | 5 | cis-acting element involved in the abscisic acid responsiveness |
| BoPRR2.2 | MBS | CAACTG | 1 | MYB binding site involved in drought-inducibility |
| BoPRR3 | ABRE | ACGTG | 1 | cis-acting element involved in the abscisic acid responsiveness |
| BoPRR3 | G-box | TACGTG | 1 | cis-acting regulatory element involved in light responsiveness |
| BoPRR3 | CAT-box | GCCACT | 3 | cis-acting regulatory element related to meristem expression |
| BoPRR3 | TGA-element | AACGAC | 1 | auxin-responsive element |
| BoPRR3 | TGACG-motif | TGACG | 3 | cis-acting regulatory element involved in the MeJA-responsiveness |
| BoPRR3 | TCT-motif | TCTTAC | 2 | part of a light responsive element |
| BoPRR3 | GT1-motif | GGTTAA | 2 | light responsive element |
| BoPRR3 | P-box | CCTTTTG | 1 | gibberellin-responsive element |
| BoPRR3 | MBS | CAACTG | 1 | MYB binding site involved in drought-inducibility |
| BoPRR3 | LTR | CCGAAA | 1 | cis-acting element involved in low-temperature responsiveness |
| BoPRR3 | CGTCA-motif | CGTCA | 3 | cis-acting regulatory element involved in the MeJA-responsiveness |
| BoPRR3 | MRE | AACCTAA | 1 | MYB binding site involved in light responsiveness |
| BoPRR4 | ARE | AAACCA | 3 | cis-acting regulatory element essential for the anaerobic induction |
| BoPRR4 | LTR | CCGAAA | 1 | cis-acting element involved in low-temperature responsiveness |
| BoPRR4 | Box 4 | ATTAAT | 2 | part of a conserved DNA module involved in light responsiveness |
| BoPRR4 | MBS | CAACTG | 2 | MYB binding site involved in drought-inducibility |
| BoPRR4 | TCA-element | CCATCTTTTT | 1 | cis-acting element involved in salicylic acid responsiveness |
| BoPRR4 | P-box | CCTTTTG | 1 | gibberellin-responsive element |
| BoPRR4 | TCT-motif | TCTTAC | 1 | part of a light responsive element |
| BoPRR4 | TGACG-motif | TGACG | 1 | cis-acting regulatory element involved in the MeJA-responsiveness |
| BoPRR4 | TATC-box | TATCCCA | 1 | cis-acting element involved in gibberellin-responsiveness |
| BoPRR4 | CGTCA-motif | CGTCA | 1 | cis-acting regulatory element involved in the MeJA-responsiveness |
| BoPRR4 | AuxRR-core | GGTCCAT | 1 | cis-acting regulatory element involved in auxin responsiveness |
| BoPRR5 | TGACG-motif | TGACG | 2 | cis-acting regulatory element involved in the MeJA-responsiveness |
| BoPRR5 | ARE | AAACCA | 3 | cis-acting regulatory element essential for the anaerobic induction |
| BoPRR5 | I-box | ccttatcct | 1 | part of a light responsive element |
| BoPRR5 | TC-rich repeats | ATTCTCTAAC | 1 | cis-acting element involved in defense and stress responsiveness |
| BoPRR5 | G-box | ACACGTGT | 5 | cis-acting regulatory element involved in light responsiveness |
| BoPRR5 | GT1-motif | GGTTAAT | 3 | light responsive element |
| BoPRR5 | Box 4 | ATTAAT | 2 | part of a conserved DNA module involved in light responsiveness |
| BoPRR5 | CAT-box | GCCACT | 2 | cis-acting regulatory element related to meristem expression |
| BoPRR5 | circadian | CAAAGATATC | 2 | cis-acting regulatory element involved in circadian control |
| BoPRR5 | CGTCA-motif | CGTCA | 2 | cis-acting regulatory element involved in the MeJA-responsiveness |
| BoPRR5 | MSA-like | TCCAACGGT | 3 | cis-acting element involved in cell cycle regulation |
| BoPRR5 | ABRE | CACGTG | 9 | cis-acting element involved in the abscisic acid responsiveness |
| BoPRR5 | G-Box | CACGTG | 5 | cis-acting regulatory element involved in light responsiveness |
| BoPRR5 | O2-site | GATGATGTGG | 1 | cis-acting regulatory element involved in zein metabolism regulation |
| BoPRR5 | LTR | CCGAAA | 1 | cis-acting element involved in low-temperature responsiveness |
| BoPRR5 | AuxRR-core | GGTCCAT | 1 | cis-acting regulatory element involved in auxin responsiveness |
| BoPRR5 | TATC-box | TATCCCA | 1 | cis-acting element involved in gibberellin-responsiveness |
| BoPRR5 | GATA-motif | AAGGATAAGG | 1 | part of a light responsive element |
| BoPRR6.1 | AT1-motif | AATTATTTTTTATT | 1 | part of a light responsive module |
| BoPRR6.1 | Box 4 | ATTAAT | 2 | part of a conserved DNA module involved in light responsiveness |
| BoPRR6.1 | AuxRR-core | GGTCCAT | 1 | cis-acting regulatory element involved in auxin responsiveness |
| BoPRR6.1 | TCA-element | CCATCTTTTT | 2 | cis-acting element involved in salicylic acid responsiveness |
| BoPRR6.1 | GT1-motif | GGTTAA | 2 | light responsive element |
| BoPRR6.1 | Gap-box | CAAATGAA(A/G)A | 1 | part of a light responsive element |
| BoPRR6.1 | O2-site | GATGA(C/T)(A/G)TG(A/G) | 1 | cis-acting regulatory element involved in zein metabolism regulation |
| BoPRR6.1 | TCT-motif | TCTTAC | 2 | part of a light responsive element |
| BoPRR6.1 | G-box | CACGAC | 1 | cis-acting regulatory element involved in light responsiveness |
| BoPRR6.2 | chs-CMA1a | TTACTTAA | 1 | part of a light responsive element |
| BoPRR6.2 | GARE-motif | TCTGTTG | 1 | gibberellin-responsive element |
| BoPRR6.2 | MBSI | aaaAaaC(G/C)GTTA | 1 | MYB binding site involved in flavonoid biosynthetic genes regulation |
| BoPRR6.2 | AE-box | AGAAACAA | 1 | part of a module for light response |
| BoPRR6.2 | CGTCA-motif | CGTCA | 1 | cis-acting regulatory element involved in the MeJA-responsiveness |
| BoPRR6.2 | G-box | CCACGTAA | 3 | cis-acting regulatory element involved in light responsiveness |
| BoPRR6.2 | TGACG-motif | TGACG | 1 | cis-acting regulatory element involved in the MeJA-responsiveness |
| BoPRR6.2 | ABRE | ACGTG | 2 | cis-acting element involved in the abscisic acid responsiveness |
| BoPRR6.2 | GA-motif | ATAGATAA | 1 | part of a light responsive element |
| BoPRR6.2 | LTR | CCGAAA | 1 | cis-acting element involved in low-temperature responsiveness |
| BoPRR6.2 | ARE | AAACCA | 1 | cis-acting regulatory element essential for the anaerobic induction |
| BoPRR6.2 | TC-rich repeats | ATTCTCTAAC | 2 | cis-acting element involved in defense and stress responsiveness |
| BoPRR6.2 | MBS | CAACTG | 2 | MYB binding site involved in drought-inducibility |
| BoPRR6.2 | TCT-motif | TCTTAC | 2 | part of a light responsive element |
| BoPRR6.2 | Box 4 | ATTAAT | 3 | part of a conserved DNA module involved in light responsiveness |
| BoPRR6.2 | AuxRR-core | GGTCCAT | 1 | cis-acting regulatory element involved in auxin responsiveness |
| BoPRR7.1 | CGTCA-motif | CGTCA | 9 | cis-acting regulatory element involved in the MeJA-responsiveness |
| BoPRR7.1 | TCA-element | CCATCTTTTT | 1 | cis-acting element involved in salicylic acid responsiveness |
| BoPRR7.1 | LAMP-element | CCTTATCCA | 1 | part of a light responsive element |
| BoPRR7.1 | ABRE | ACGTG | 8 | cis-acting element involved in the abscisic acid responsiveness |
| BoPRR7.1 | CAT-box | GCCACT | 2 | cis-acting regulatory element related to meristem expression |
| BoPRR7.1 | ARE | AAACCA | 1 | cis-acting regulatory element essential for the anaerobic induction |
| BoPRR7.1 | TCCC-motif | TCTCCCT | 3 | part of a light responsive element |
| BoPRR7.1 | G-box | CACGAC | 7 | cis-acting regulatory element involved in light responsiveness |
| BoPRR7.1 | GT1-motif | GGTTAA | 3 | light responsive element |
| BoPRR7.1 | P-box | CCTTTTG | 1 | gibberellin-responsive element |
| BoPRR7.1 | AE-box | AGAAACTT | 2 | part of a module for light response |
| BoPRR7.1 | TC-rich repeats | ATTCTCTAAC | 1 | cis-acting element involved in defense and stress responsiveness |
| BoPRR7.1 | TGACG-motif | TGACG | 9 | cis-acting regulatory element involved in the MeJA-responsiveness |
| BoPRR7.1 | TCT-motif | TCTTAC | 1 | part of a light responsive element |
| BoPRR7.2 | AE-box | AGAAACTT | 1 | part of a module for light response |
| BoPRR7.2 | TCA-element | TCAGAAGAGG | 1 | cis-acting element involved in salicylic acid responsiveness |
| BoPRR7.2 | LTR | CCGAAA | 1 | cis-acting element involved in low-temperature responsiveness |
| BoPRR7.2 | GARE-motif | TCTGTTG | 1 | gibberellin-responsive element |
| BoPRR7.2 | CAT-box | GCCACT | 2 | cis-acting regulatory element related to meristem expression |
| BoPRR7.2 | chs-CMA2a | TCACTTGA | 1 | part of a light responsive element |
| BoPRR7.2 | ATCT-motif | AATCTAATCC | 1 | part of a conserved DNA module involved in light responsiveness |
| BoPRR7.2 | TGACG-motif | TGACG | 2 | cis-acting regulatory element involved in the MeJA-responsiveness |
| BoPRR7.2 | P-box | CCTTTTG | 1 | gibberellin-responsive element |
| BoPRR7.2 | GT1-motif | GGTTAA | 1 | light responsive element |
| BoPRR7.2 | CGTCA-motif | CGTCA | 2 | cis-acting regulatory element involved in the MeJA-responsiveness |
| BoPRR7.2 | ATC-motif | AGTAATCT | 2 | part of a conserved DNA module involved in light responsiveness |
| BoPRR7.2 | ABRE | ACGTG | 1 | cis-acting element involved in the abscisic acid responsiveness |
| BoPRR7.2 | Box 4 | ATTAAT | 2 | part of a conserved DNA module involved in light responsiveness |
| BoPRR7.2 | G-box | CACGAC | 1 | cis-acting regulatory element involved in light responsiveness |
| BoPRR7.2 | ARE | AAACCA | 6 | cis-acting regulatory element essential for the anaerobic induction |
| BoPRR9.1 | G-box | GCCACGTGGA | 2 | cis-acting regulatory element involved in light responsiveness |
| BoPRR9.1 | GT1-motif | GGTTAA | 2 | light responsive element |
| BoPRR9.1 | ABRE | AACCCGG | 5 | cis-acting element involved in the abscisic acid responsiveness |
| BoPRR9.1 | AE-box | AGAAACTT | 1 | part of a module for light response |
| BoPRR9.1 | ARE | AAACCA | 3 | cis-acting regulatory element essential for the anaerobic induction |
| BoPRR9.1 | Box 4 | ATTAAT | 1 | part of a conserved DNA module involved in light responsiveness |
| BoPRR9.1 | CGTCA-motif | CGTCA | 2 | cis-acting regulatory element involved in the MeJA-responsiveness |
| BoPRR9.1 | G-Box | CACGTT | 2 | cis-acting regulatory element involved in light responsiveness |
| BoPRR9.1 | ATCT-motif | AATCTAATCC | 1 | part of a conserved DNA module involved in light responsiveness |
| BoPRR9.1 | TCT-motif | TCTTAC | 2 | part of a light responsive element |
| BoPRR9.1 | ACE | GACACGTATG | 1 | cis-acting element involved in light responsiveness |
| BoPRR9.1 | TGACG-motif | TGACG | 2 | cis-acting regulatory element involved in the MeJA-responsiveness |
| BoPRR9.1 | chs-CMA1a | TTACTTAA | 1 | part of a light responsive element |
| BoPRR9.2 | Box 4 | ATTAAT | 3 | part of a conserved DNA module involved in light responsiveness |
| BoPRR9.2 | CGTCA-motif | CGTCA | 1 | cis-acting regulatory element involved in the MeJA-responsiveness |
| BoPRR9.2 | GT1-motif | GGTTAA | 1 | light responsive element |
| BoPRR9.2 | TCA-element | CCATCTTTTT | 2 | cis-acting element involved in salicylic acid responsiveness |
| BoPRR9.2 | TC-rich repeats | ATTCTCTAAC | 1 | cis-acting element involved in defense and stress responsiveness |
| BoPRR9.2 | ABRE | CGCACGTGTC | 6 | cis-acting element involved in the abscisic acid responsiveness |
| BoPRR9.2 | TGACG-motif | TGACG | 1 | cis-acting regulatory element involved in the MeJA-responsiveness |
| BoPRR9.2 | MRE | AACCTAA | 1 | MYB binding site involved in light responsiveness |
| BoPRR9.2 | ARE | AAACCA | 5 | cis-acting regulatory element essential for the anaerobic induction |
| BoPRR9.2 | AuxRR-core | GGTCCAT | 1 | cis-acting regulatory element involved in auxin responsiveness |
| BoPRR9.2 | AE-box | AGAAACAA | 1 | part of a module for light response |
| BoPRR9.2 | CAG-motif | GAAAGGCAGAC | 1 | part of a light response element |
| BoPRR9.2 | O2-site | GATGA(C/T)(A/G)TG(A/G) | 1 | cis-acting regulatory element involved in zein metabolism regulation |
| BoPRR9.2 | G-box | CACGTG | 6 | cis-acting regulatory element involved in light responsiveness |
| BoPRR9.2 | TGA-element | AACGAC | 1 | auxin-responsive element |
