# Supplementary material for: Genome-wide identification and expression profiling of two-component system (TCS) genes in Brassica oleracea in response to shade stress
Source: Front Genet. 2023 May 30;14:1142544. doi: 10.3389/fgene.2023.1142544 (PMC10267837; doi:10.3389/fgene.2023.1142544)
Supplement: Supplementary file 9 [file Table3.DOCX]

**Table S3:** Prediction of conserved domains by using various databases.

| **Family** | **Gene Name** | **Gene ID** | **Pfam** | **SMART** | **CDD** |
| --- | --- | --- | --- | --- | --- |
| **Histidine Kinases (HKs)** | BoHK1 | LOC106301461 | HATPase_c, Response_reg, HisKA | HisKA, HATPase_c, REC | PRK11107 super family |
|  | BoHK2.1 | LOC106342170 | CHASE, HATPase_c, Response_reg, HisKA | CHASE, HisKA, HATPase_c, REC | PRK11107 super family, CHASE |
|  | BoHK2.2 | LOC106342170 | CHASE, HATPase_c, Response_reg, HisKA | CHASE, HisKA, HATPase_c, REC | PRK11107 super family, CHASE |
|  | BoHK3 | LOC106306128 | CHASE, HATPase_c, Response_reg, HisKA | CHASE, HisKA, HATPase_c, REC | PRK11107 super family, CHASE |
|  | BoHK4 | LOC106306308 | HATPase_c, Response_reg, HisKA | HisKA, HATPase_c, REC | PRK11107 super family, CHASE |
|  | BoHK5.1 | BOLC3T13054H | HATPase_c, Response_reg, HisKA | HisKA, HATPase_c, REC | PRK11107 super family, Smc super family |
|  | BoHK5.2 | LOC106327247 | HATPase_c, Response_reg, HisKA | HisKA, HATPase_c, REC | PRK11107 super family, Smc super family |
|  | BoCKI1.1 | LOC106341550 | HATPase_c, Response_reg, HisKA | HisKA, HATPase_c, REC | PRK11107 super family, BaeS |
|  | BoCKI1.2 | LOC106340803 | HATPase_c, Response_reg, HisKA | HisKA, HATPase_c, REC | REC_hyHK_CKI1_RcsC-like, BaeS |
|  | BoERS1 | LOC106342958 | GAF, HATPase_c, HisKA | GAF, HisKA, HATPase_c | PRK11107 super family, GAF |
|  | BoERS2.1 | LOC106307314 | GAF | GAF, HisKA | HATPase super family, GAF, PRK11466 super family |
|  | BoERS2.2 | LOC106343671 | GAF | GAF, HisKA | HATPase super family, GAF, PRK11466 super family |
|  | BoETR1 | LOC106299922 | GAF, HATPase_c, HisKA, Response_reg | GAF, HATPase_c, HisKA, REC | PRK11107 super family, GAF |
|  | BoETR2 | LOC106316002 | GAF,  Response_reg | GAF, HisKA, HATPase_c, REC | REC, HATPase_c, GAF, HisKA |
|  | BoEIN4 | LOC106334407 | GAF, HisKA, Response_reg | GAF, HisKA, HATPase_c, REC | REC, HATPase_c, GAF, HisKA |
| **Phytochromes** | BoPHYAa | LOC106294344 | PAS_2, GAF, PHY, PAS, PAS, HATPase_c | PHY, PAS, PAS, PAS_2, GAF, HATPase_c, HisKA | COG4251 super family, HATPase_Phy-like, PAS, BaeS, PAS |
|  | BoPHYAb | LOC106311538 | PHY, PAS, PAS, PAS_2, GAF, HATPase_c, HisKA | PHY, PAS, PAS, PAS_2, GAF, HATPase_c, HisKA | COG4251 super family, HATPase_Phy-like, PAS, BaeS, PAS |
|  | BoPHYB | BOLC3T17135H | PHY, PAS, PAS, PAS_2, GAF, HATPase_c, HisKA | PHY, PAS, PAS, PAS_2, GAF, HATPase_c | COG4251 super family, HATPase_Phy-like, PAS, PAS, PRK09959 super family |
|  | BoPHYC | LOC106300333 | PAS_2, GAF, PHY, PAS, PAS, HATPase_c | PHY, PAS, PAS, PAS_2, GAF, HATPase_c | COG4251 super family, HATPase_Phy-like, PAS, PAS, PRK09959 super family |
|  | BoPHYD | LOC106294529 | PHY, PAS, PAS, PAS_2, GAF, HATPase_c, HisKA | PHY, PAS, PAS, PAS_2, GAF, HATPase_c, HisKA | COG4251 super family, HATPase_Phy-like, PAS, BaeS, PAS |
|  | BoPHYE | LOC106305038 | PHY, PAS, PAS, PAS_2, GAF, HATPase_c, HisKA | PHY, PAS, PAS, PAS_2, GAF, HATPase_c, HisKA | COG4251 super family, HATPase_Phy-like, PAS, BaeS, PAS |
| **Phosphotransfer Proteins (HPs)** | BoHP1 | LOC106296168 | Hpt | Hpt | Hpt |
|  | BoHP2.1 | LOC106316247 | Hpt | HPT | Hpt |
|  | BoHP2.2 | LOC106305910 | Hpt | Hpt | Hpt |
|  | BoHP3 | LOC106339576 | Hpt | Hpt | Hpt |
|  | BoHP4.1 | LOC106293908 | Hpt | Hpt | Hpt |
|  | BoHP4.2 | LOC106333468 | Hpt | Hpt | Hpt |
|  | BoHP5 | LOC106293046 | Hpt | Hpt | Hpt |
|  | BoHP6 | LOC106299699 | Hpt | Hpt | Hpt |
| **Type-A Response Regulators (RRs)** | BoRR3 | LOC106316622 | Hpt | REC | REC_typeA_ARR |
|  | BoRR4.1 | LOC106308087 | Response_reg | REC | REC_typeA_ARR |
|  | BoRR4.2 | LOC106294617 | Response_reg | REC | REC_typeA_ARR |
|  | BoRR5.1 | LOC106308235 | Response_reg | REC | REC_typeA_ARR |
|  | BoRR5.2 | LOC106325353 | Response_reg | REC | REC |
|  | BoRR6.1 | LOC103874727 | Response_reg | REC | REC |
|  | BoRR6.2 | LOC106328967 | Response_reg | REC | REC |
|  | BoRR7.1 | LOC106308136 | Response_reg | REC | REC_typeA_ARR |
|  | BoRR7.2 | LOC106296156 | Response_reg | REC | REC_typeA_ARR |
|  | BoRR8.1 | LOC106339904 | Response_reg | REC | PLN03029 super family |
|  | BoRR8.2 | LOC106339761 | Response_reg | REC | PLN03029 super family |
|  | BoRR9.1 | LOC106307836 | Response_reg | REC | PLN03029 super family |
|  | BoRR9.2 | LOC106341996 | Response_reg | REC | PLN03029 super family |
|  | BoRR15.1 | LOC106298524 | Response_reg | REC | REC_typeA_ARR |
|  | BoRR15.2 | LOC106298210 | Response_reg | REC | REC_typeA_ARR |
|  | BoRR16 | LOC106331794 | Response_reg | REC | REC_typeA_ARR |
|  | BoRR17.1 | LOC106307456 | Response_reg | REC | REC_typeA_ARR |
|  | BoRR17.2 | LOC106341075 | Response_reg | REC | REC_typeA_ARR |
| **Type-B Response Regulators (RRs)** | BoRR1.1 | LOC106295322 | Response_reg, Myb_DNA-binding | REC, Myb_DNA-binding | REC_typeB_ARR-like, myb_SHAQKYF |
|  | BoRR1.2 | LOC106335935 | Response_reg, Myb_DNA-binding | REC, Myb_DNA-binding | REC_typeB_ARR-like, myb_SHAQKYF, PLN03162 super family |
|  | BoRR2.1 | LOC106306398 | Response_reg, Myb_DNA-binding | REC, Myb_DNA-binding | REC_typeB_ARR-like, myb_SHAQKYF |
|  | BoRR2.2 | LOC106309040 | Response_reg, Myb_DNA-binding | Response_reg, Myb_DNA-binding | REC_typeB_ARR-like, myb_SHAQKYF, PLN03162 super family |
|  | BoRR10.1 | LOC106307094 | Response_reg, Myb_DNA-binding | REC, Myb_DNA-binding | REC_typeB_ARR-like, myb_SHAQKYF |
|  | BoRR10.2 | LOC106327785 | Response_reg | REC | REC_typeB_ARR-like, myb_SHAQKYF |
|  | BoRR11 | LOC106300628 | Response_reg, Myb_DNA-binding | REC, Myb_DNA-binding | REC_typeB_ARR-like, myb_SHAQKYF, PLN03162 super family |
|  | BoRR12 | LOC106336761 | Response_reg, Myb_DNA-binding | REC, Myb_DNA-binding | REC_typeB_ARR-like, myb_SHAQKYF |
|  | BoRR13 | LOC106333471 | Response_reg | REC | REC, myb_SHAQKYF |
|  | BoRR14 | LOC106322664 | Response_reg, Myb_DNA-binding | REC, Myb_DNA-binding | REC_typeB_ARR-like, myb_SHAQKYF |
|  | BoRR18 | LOC106325869 | Response_reg, Myb_DNA-binding | REC, Myb_DNA-binding | REC_typeB_ARR-like, myb_SHAQKYF |
|  | BoRR19.1 | LOC106319855 | Response_reg | REC, Myb_DNA-binding | REC_typeB_ARR-like, myb_SHAQKYF |
|  | BoRR19.2 | LOC106320395 | Response_reg | REC | REC , myb-like DNA-binding domain, PLN03162 super family |
|  | BoRR20 | LOC106310773 | Response_reg, Myb_DNA-binding | REC, Myb_DNA-binding | REC_typeB_ARR-like, myb_SHAQKYF |
|  | BoRR21 | LOC106317158 | Response_reg, Myb_DNA-binding | REC, Myb_DNA-binding | REC_typeB_ARR-like, myb_SHAQKYF, PLN03162 super family |
|  | BoRR23 | LOC106330569 | Response_reg | REC | REC super family, DUF5401 super family |
| **Type-c Response Regulators (RRs)** | BoRR22.1 | LOC106294222 | Response_reg | REC | REC_hyHK_CKI1_RcsC-like |
|  | BoRR22.2 | LOC106333911 | Response_reg | REC | REC_hyHK_CKI1_RcsC-like |
|  | BoRR24.1 | LOC106315485 | Response_reg | REC | REC_hyHK_CKI1_RcsC-like |
|  | BoRR24.2 | LOC106327368 | Response_reg | REC | REC_hyHK_CKI1_RcsC-like |
|  | BoRR24.3 | LOC106326552 | Response_reg | REC | REC_hyHK_CKI1_RcsC-like |
| **Pseudo-Response Regulators (PRRs)** | BoPRR1 | LOC106312920 | Response_reg, CCT | REC | REC super family, CCT |
|  | BoPRR2.1 | LOC106301155 | Myb_DNA-binding | REC, CCT | PLN03162 super family, REC super family, CCT, KLF_1_2_4_N super family |
|  | BoPRR2.2 | LOC106324983 | Response_reg, Myb_DNA-binding | REC, Myb_DNA-binding | PLN03162 super family, REC |
|  | BoPRR3 | LOC106319487 | Response_reg, CCT | REC, CCT | PsREC_PRR, CCT |
|  | BoPRR4 | LOC106305193 | Response_reg | REC | REC_typeB_ARR-like, myb_SHAQKYF |
|  | BoPRR5 | LOC106306444 | Response_reg, CCT | REC, CCT | psREC_PRR, CCT |
|  | BoPRR6.1 | LOC106298038 | Response_reg | REC | REC super family, PLN03029 super family |
|  | BoPRR6.2 | LOC106299620 | Response_reg | REC | REC super family, PLN03029 super family |
|  | BoPRR7.1 | LOC106317211 | Response_reg, CCT | REC, CCT | PsREC_PRR, CCT |
|  | BoPRR7.2 | LOC106327018 | Response_reg, CCT | REC, CCT | PsREC_PRR, CCT |
|  | BoPRR9.1 | LOC106341950 | Response_reg, CCT | REC, CCT | PsREC_PRR, CCT |
|  | BoPRR9.2 | LOC106337167 | Response_reg, CCT | REC, CCT | PsREC_PRR, CCT |
