# Supplementary material for: Genome-wide identification and expression profiling of two-component system (TCS) genes in Brassica oleracea in response to shade stress
Source: Front Genet. 2023 May 30;14:1142544. doi: 10.3389/fgene.2023.1142544 (PMC10267837; doi:10.3389/fgene.2023.1142544)
Supplement: Supplementary file 10 [file Table4.DOCX]

**Table S4:** Physiochemical characteristics of BoTCSs.

| **Accession no** | **Final name** | **Chr** | **Start Site** | **End Site** | **Exon** | **Protein Length (aa)** | **Molecular weight (MW)** | **Isoelectric point (PI)** | **Instability index (II)** | **Aliphatic index** | **Grand average of hydropathicity (GRAVY)** | **Subcellular localization** |
| --- | --- | --- | --- | --- | --- | --- | --- | --- | --- | --- | --- | --- |
| **Histidine kinases (HKs)** | |  |  |  |  |  |  |  |  |  |  |  |
| XP_013593316.1 | BoHK1 | C7 | 4530668 | 4536510 | 13 | 1192 | 133699.23 | 6.33 | 39.48 | 94.53 | -0.217 | Plasma Membrane |
| XP_013636483.1 | BoHK2.1 | C4 | 34817137 | 34822488 | 14 | 1151 | 128383.22 | 6.3 | 42.36-unst | 91.03 | -0.197 | Plasma Membrane, Cytoplasmic, Nuclear |
| VDC94463.1 | BoHK2.2 | C3 | 31726378 | 31732135 | 11 | 1124 | 125392.33 | 5.62 | 45.99-unst | 87.66 | -0.205 | Plasma Membrane, Nuclear, Cytoplasmic |
| XP_013598058.1 | BoHK3 | C7 | 18294871 | 18299099 | 11 | 1019 | 114206.18 | 6.46 | 43.19-unst | 88.26 | -0.161 | Plasma Membrane |
| XP_013598332.1 | BoHK4 | C7 | 30271801 | 30277214 | 9 | 1052 | 116373.97 | 6.38 | 39.86 | 86.11 | -0.195 | Cytoplasmic, Plasma Membrane |
| VDC85847.1 | BoHK5.1 | C3 | 2336256 | 2342628 | 11 | 992 | 111969.71 | 5.34 | 51.73-unst | 85.69 | -0.421 | Nuclear |
| XP_013620805.1 | BoHK5.2 | C2 | 2239232 | 2243790 | 9 | 918 | 103431.5 | 5.03 | 48.98-unst | 84.3 | -0.488 | Nuclear |
| XP_013635744.1 | BoCKI1.1 | C4 | 33152532 | 33156723 | 7 | 1030 | 113914.38 | 8.7 | 38.29 | 83.89 | -0.251 | Plasma Membrane, Cytoplasmic |
| XP_013635095.1 | BoCKI1.2 | C4 | 331567 | 335865 | 6 | 1100 | 121493.65 | 6.47 | 42.26-unst | 88.09 | -0.205 | Plasma Membrane |
| XP_013637485.1 | BoERS1 | C4 | 104923 | 107738 | 6 | 613 | 68145.48 | 6.1 | 42.63-unst | 106.61 | 0.174 | Plasma Membrane |
| XP_013599690.1 | BoERS2.1 | C8 | 555481 | 558226 | 3 | 620 | 68257.26 | 8.12 | 34.08 | 98.65 | 0.131 | Plasma Membrane |
| XP_013638399.1 | BoERS2.2 | C5 | 1252444 | 1255057 | 3 | 618 | 68266.22 | 8.57 | 34.35 | 98.04 | 0.104 | Plasma Membrane |
| XP_013591391.1 | BoETR1 | C6 | 31832788 | 31836335 | 7 | 736 | 82261.24 | 7.34 | 38.2 | 109.62 | 0.138 | Plasma Membrane |
| XP_013609348.1 | BoETR2 | C1 | 31187087 | 31190249 | 3 | 780 | 86450.36 | 6.52 | 42.35-unst | 98.78 | 0.082 | Plasma Membrane |
| XP_013628174.1 | BoEIN4 | C1 | 42648119 | 42651583 | 2 | 766 | 86216.73 | 8.54 | 45.13-unst | 102.28 | 0.029 | Plasma Membrane |
| XP_013585349.1 | BoPHYAa | C5 | 3716341 | 3721601 | 7 | 1124 | 124401.7 | 5.96 | 43.76-unst | 91.68 | -0.148 | Cytoplasmic |
| XP_013604186.1 | BoPHYAb | C8 | 39591764 | 39597097 | 4 | 1123 | 124367.79 | 5.83 | 44.10-unst | 92.02 | -0.134 | Cytoplasmic |
| VDC93793.1 | BoPHYB | C3 | 27062400 | 27067169 | 5 | 1155 | 127166.83 | 5.79 | 47.86-unst | 91.6 | -0.08 | Plasma Membrane, Cytoplasmic |
| XP_013591904.1 | BoPHYC | C6 | 15216913 | 15220635 | 3 | 1117 | 123775.08 | 5.62 | 52.95-unst | 91.35 | -0.116 | Nuclear |
| XP_013585553.1 | BoPHYD | C5 | 37926834 | 37931297 | 4 | 1168 | 128913.46 | 5.58 | 53.12-unst | 88.99 | -0.122 | Plasma Membrane, Cytoplasmic |
| XP_013596874.1 | BoPHYE | C1 | 6622027 | 6625813 | 3 | 1116 | 122675.29 | 6.29 | 43.21-unst | 89.72 | -0.166 | Cytoplasmic |
| **Phosphotransfer Proteins (HPs)** | | |  |  |  |  |  |  |  |  |  |  |
| XP_013587700.1 | BoHP1 | C1 | 32757055 | 32758618 | 6 | 154 | 17592.08 | 5.11 | 44.30-unst | 93.51 | -0.228 | Nuclear |
| XP_013609576.1 | BoHP2.1 | C9 | 2282342 | 2283778 | 6 | 156 | 17435.93 | 4.95 | 22.62 | 91.79 | -0.151 | Cytoplasmic |
| XP_013597790.1 | BoHP2.2 | C7 | 33919326 | 33920753 | 6 | 156 | 17463.88 | 4.84 | 27.45 | 87.44 | -0.208 | Cytoplasmic |
| XP_013633864.1 | BoHP3 | C4 | 36662236 | 36664143 | 7 | 155 | 17424.8 | 4.88 | 36.22 | 88.58 | -0.202 | Nuclear |
| XP_013584994.1 | BoHP4.1 | C5 | 38680137 | 38681464 | 6 | 145 | 16946.17 | 7.71 | 48.22-unst | 64.62 | -0.741 | Nuclear |
| XP_013627365.1 | BoHP4.2 | C3 | 26859213 | 26860653 | 6 | 145 | 16883.12 | 8.41 | 51.02-unst | 68 | -0.739 | Nuclear |
| XP_013584171.1 | BoHP5 | C5 | 953193 | 955019 | 7 | 156 | 17824.22 | 5.19 | 48.91-unst | 85.45 | -0.378 | Nuclear |
| XP_013591199.1 | BoHP6 | C6 | 24321653 | 24322712 | 5 | 154 | 17871.56 | 6.2 | 56.23-unst | 105.65 | -0.198 | Nuclear, Mitochondrial, Extracellular |
| **Response Regulators (RRs)** | |  |  |  |  |  |  |  |  |  |  |  |
| **Type-A RRs** |  |  |  |  |  |  |  |  |  |  |  |  |
| XP_013609959.1 | BoRR3 | C9 | 14852267 | 14853579 | 5 | 238 | 26327.35 | 4.92 | 67.16-unst | 97.73 | -0.183 | Nuclear |
| XP_013600664.1 | BoRR4.1 | C8 | 19392830 | 19394200 | 4 | 247 | 26848.73 | 4.67 | 69.62-unst | 91.82 | -0.22 | Nuclear |
| XP_013585674.1 | BoRR4.2 | C5 | 4187484 | 4188858 | 5 | 253 | 27483.17 | 4.65 | 81.76-unst | 91.98 | -0.309 | Nuclear |
| XP_013600831.1 | BoRR5.1 | C8 | 24688970 | 24691140 | 5 | 183 | 20745.08 | 5.83 | 56.4-unst | 107.05 | -0.154 | Nuclear, Cytoplasmic |
| XP_013618856.1 | BoRR5.2 | C1 | 21737346 | 21739468 | 5 | 183 | 20761.14 | 5.55 | 52.36-unst | 107.05 | -0.152 | Nuclear, Cytoplasmic |
| XP_009151412.1 | BoRR6.1 | C6 | 19625327 | 19627081 | 5 | 190 | 21550.2 | 6.18 | 46.15-unst | 102.47 | -0.176 | Nuclear, Cytoplasmic |
| VDC96237.1 | BoRR6.2 | C3 | 40671512 | 40674312 | 5 | 229 | 26154.69 | 8.66 | 50.35-unst | 107.69 | -0.038 | Nuclear |
| XP_013600719.1 | BoRR7.1 | C8 | 22323165 | 22325063 | 5 | 212 | 23333.63 | 5.65 | 55.02-unst | 92.78 | -0.304 | Nuclear |
| XP_013587680.1 | BoRR7.2 | C5 | 9309580 | 9311141 | 5 | 212 | 23291.75 | 5.52 | 52.49-unst | 94.25 | -0.268 | Nuclear |
| XP_013634228.1 | BoRR8.1 | C4 | 51438438 | 51439972 | 5 | 222 | 25070.77 | 5.53 | 69.04-unst | 85.14 | -0.515 | Nuclear |
| XP_013634086.1 | BoRR8.2 | C4 | 1653527 | 1654874 | 4 | 214 | 24209.91 | 5.32 | 51.45-unst | 86.07 | -0.473 | Nuclear |
| XP_013600356.1 | BoRR9.1 | C8 | 30936737 | 30938561 | 6 | 235 | 26236.37 | 5.46 | 54.45-unst | 93.36 | -0.354 | Cytoplasmic |
| XP_013636217.1 | BoRR9.2 | C4 | 28546033 | 28547577 | 5 | 232 | 25762.51 | 5.03 | 65.62-unst | 88.66 | -0.361 | Nuclear |
| XP_013590114.1 | BoRR15.1 | C6 | 27138165 | 27139582 | 5 | 223 | 24818.12 | 5 | 54.47-unst | 92.15 | -0.243 | Nuclear |
| XP_013589743.1 | BoRR15.2 | C6 | 37234605 | 37236149 | 5 | 185 | 20614.16 | 5.94 | 45.65-unst | 116.92 | 0.198 | Plasma Membrane, Chloroplast |
| XP_013625661.1 | BoRR16 | C3 | 13548696 | 13550604 | 5 | 158 | 17693.6 | 7.11 | 29.34 | 96.2 | -0.197 | Nuclear, Extracellular, Cytoplasmic |
| XP_013599874.1 | BoRR17.1 | C8 | 30530566 | 30531774 | 5 | 141 | 15728.41 | 6.12 | 41.35-unst | 109.15 | -0.082 | Nuclear |
| XP_013635343.1 | BoRR17.2 | C4 | 28935491 | 28936488 | 5 | 141 | 15689.37 | 6.12 | 35.57 | 108.44 | -0.065 | Nuclear, Cytoplasmic |
| **Type-B RRs** |  |  |  |  |  |  |  |  |  |  |  |  |
| XP_013586644.1 | BoRR1.1 | C5 | 38044111 | 38047958 | 7 | 680 | 74098.84 | 5.94 | 54.89-unst | 72.81 | -0.496 | Nuclear |
| VDC93784.1 | BoRR1.2 | C3 | 27031860 | 27035142 | 6 | 711 | 78743.48 | 6.95 | 53.72-unst | 73.19 | -0.49 | Nuclear |
| XP_013598455.1 | BoRR2.1 | C7 | 40035054 | 40042623 | 6 | 600 | 65834.76 | 6.21 | 51.01-unst | 70.63 | -0.537 | Nuclear |
| XP_013601580.1 | BoRR2.2 | C1 | 16125005 | 16128095 | 6 | 631 | 68971.17 | 6.08 | 51.3-unst | 70.25 | -0.554 | Nuclear |
| XP_013599414.1 | BoRR10.1 | C7 | 46039174 | 46041938 | 6 | 588 | 65877.4 | 5.78 | 47.47-unst | 77.6 | -0.497 | Nuclear |
| XP_013621492.1 | BoRR10.2 | C1 | 3723913 | 3726517 | 7 | 506 | 56683.03 | 6.02 | 47.45-unst | 73.99 | -0.524 | Nuclear |
| XP_013592266.1 | BoRR11 | C6 | 31128457 | 31130827 | 5 | 507 | 57127.69 | 5.23 | 43.92-unst | 75.92 | -0.521 | Nuclear |
| XP_013631146.1 | BoRR12 | C4 | 42951682 | 42954647 | 6 | 577 | 63465.09 | 6.06 | 43.75-unst | 74.68 | -0.571 | Nuclear |
| XP_013627368.1 | BoRR13 | C3 | 1549839 | 1554334 | 9 | 762 | 85582.1 | 5.8 | 50.36-unst | 67.53 | -0.688 | Nuclear |
| XP_013616214.1 | BoRR14 | C2 | 42282388 | 42284511 | 5 | 376 | 41574.53 | 9.01 | 55.23-unst | 88.38 | -0.286 | Nuclear |
| XP_013619374.1 | BoRR18 | C2 | 8656579 | 8659419 | 6 | 626 | 69897.14 | 5.37 | 56.27-unst | 68.16 | -0.61 | Nuclear |
| XP_013613707.1 | BoRR19.1 | Un | 35756 | 37702 | 5 | 450 | 51202.95 | 5.41 | 61.08-unst | 76.22 | -0.601 | Nuclear |
| XP_013614206.1 | BoRR19.2 | Un | 46877 | 49015 | 5 | 550 | 62229.72 | 5.67 | 50.31-unst | 69.98 | -0.71 | Nuclear |
| XP_013603444.1 | BoRR20 | C8 | 33485627 | 33487575 | 5 | 419 | 48035.87 | 5.12 | 41.39-unst | 69.79 | -0.688 | Nuclear |
| XP_013610469.1 | BoRR21 | C9 | 51927425 | 51931491 | 9 | 772 | 85939.83 | 5.65 | 47.16-unst | 64.94 | -0.695 | Nuclear |
| XP_013624468.1 | BoRR23 | C3 | 38531948 | 38542559 | 13 | 1423 | 157324.21 | 7.8 | 45.27-unst | 71.69 | -0.623 | Nuclear |
| **Type-C RRs** |  |  |  |  |  |  |  |  |  |  |  |  |
| XP_013585250.1 | BoRR22.1 | C5 | 45659905 | 45660815 | 3 | 136 | 15055.57 | 5.41 | 27.93 | 104.71 | -0.207 | Cytoplasmic |
| XP_013627750.1 | BoRR22.2 | C3 | 22339618 | 22340621 | 3 | 141 | 15530.07 | 6.31 | 28.76 | 101.7 | -0.257 | Cytoplasmic |
| XP_013608681.1 | BoRR24.1 | C9 | 3386385 | 3387636 | 2 | 134 | 15061.28 | 4.99 | 30.62 | 97.46 | -0.232 | Cytoplasmic, Nuclear, Chloroplast |
| VDD26937.1 | BoRR24.2 | C2 | 49022011 | 49028900 | 19 | 1327 | 146733.58 | 5.55 | 39.13 | 91.45 | -0.242 | Cytoplasmic, Nuclear, Chloroplast |
| XP_013619955.1 | BoRR24.3 | C2 | 49029414 | 49030090 | 2 | 134 | 15030.16 | 4.82 | 36.63 | 98.13 | -0.211 | Cytoplasmic, Chloroplast |
| **Pseudo-RRs** |  |  |  |  |  |  |  |  |  |  |  |  |
| XP_013606067.1 | BoPRR1 | C9 | 4417977 | 4421016 | 6 | 523 | 59289.59 | 8.09 | 55.66-unst | 65.22 | -0.875 | Nuclear |
| XP_013592946.1 | BoPRR2.1 | C7 | 40830402 | 40834468 | 10 | 519 | 57739.94 | 5.76 | 54.69-unst | 74.14 | -0.66 | Nuclear |
| XP_013618779.1 | BoPRR2.2 | C1 | 6576121 | 6580053 | 10 | 505 | 56437.79 | 6.33 | 53.10-usnt | 74.26 | -0.632 | Nuclear |
| XP_013613286.1 | BoPRR3 | C9 | 42961948 | 42965082 | 11 | 471 | 51555.81 | 5.63 | 50.96-unst | 65.18 | -0.832 | Nuclear |
| XP_013597017.1 | BoPRR4 | C7 | 35358591 | 35367547 | 6 | 509 | 58938.81 | 5.71 | 61.64-unst | 72.57 | -0.86 | Nuclear |
| XP_013598521.1 | BoPRR5 | C7 | 37472556 | 37475295 | 7 | 558 | 62102.33 | 6.96 | 58.89-unst | 66.4 | -0.745 | Nuclear |
| XP_013589599.1 | BoPRR6.1 | C6 | 33089477 | 33091892 | 7 | 592 | 66640.65 | 5.04 | 50.50-unst | 80.56 | -0.585 | Nuclear |
| VDD63815.1 | BoPRR6.2 | C6 | 33228604 | 33229837 | 5 | 305 | 35151.21 | 9.2 | 46.76-unst | 87.9 | -0.537 | Nuclear |
| XP_013610513.1 | BoPRR7.1 | C9 | 54158055 | 54161843 | 10 | 701 | 76350.09 | 6.82 | 43.76-unst | 62.4 | -0.858 | Nuclear |
| XP_013620469.1 | BoPRR7.2 | C2 | 506590 | 510184 | 9 | 689 | 75273.07 | 7.13 | 45.55-unst | 61.38 | -0.88 | Nuclear |
| XP_013636162.1 | BoPRR9.1 | C4 | 693116 | 695039 | 4 | 413 | 46089.02 | 6.54 | 50.38-unst | 75.08 | -0.652 | Nuclear |
| XP_013631677.1 | BoPRR9.2 | C4 | 53379196 | 53381930 | 6 | 471 | 51829.1 | 6.23 | 48.45-unst | 69.75 | -0.67 | Nuclear |
